# Prevalence of Perinatal Depression in Low- and Middle-Income Countries: A Systematic Review and Meta-analysis

**DOI:** 10.1001/jamapsychiatry.2023.0069

**Published:** 2023-03-08

**Authors:** Alexandra Roddy Mitchell, Hannah Gordon, Anthea Lindquist, Susan P. Walker, Caroline S. E. Homer, Anna Middleton, Catherine A. Cluver, Stephen Tong, Roxanne Hastie

**Affiliations:** 1 Department of Obstetrics and Gynaecology, The University of Melbourne, Heidelberg, Victoria, Australia; 2 Mercy Perinatal, Mercy Hospital for Women, Heidelberg, Victoria, Australia; 3 Maternal, Child and Adolescent Health Program, Burnet Institute, Melbourne, Victoria, Australia; 4 Department of Obstetrics and Gynaecology, Stellenbosch University, Tygerberg Hospital, Cape Town, South Africa

## Abstract

**Importance:**

Women who experience depression during or within a year of pregnancy are at increased risk of morbidity and mortality. Although those living in low- and middle-income countries are thought to be at increased risk of perinatal depression, the true prevalence remains unclear.

**Objective:**

To determine the prevalence of depression among individuals living in low- and middle-income countries during pregnancy and up 1 year post partum.

**Data Sources:**

MEDLINE, Embase, PsycINFO, CINAHL, Web of Science, and the Cochrane Library were searched from database inception until April 15, 2021.

**Study Selection:**

Studies were included that reported the prevalence of depression using a validated method during pregnancy or up to 12 months post partum in countries defined by the World Bank as low, lower-middle, and upper-middle income.

**Data Extraction and Synthesis:**

This study followed Preferred Reporting Items for Systematic Reviews and Meta-analyses (PRISMA) reporting guideline. Two reviewers independently assessed study eligibility, extracted data, and assessed studies for bias. Prevalence estimates were calculated using a random-effects meta-analysis model. Subgroup analyses were performed among women who were considered at increased risk of developing perinatal depression.

**Main Outcomes and Measures:**

Point prevalence of perinatal depression was the main outcome measured as percentage point estimates with corresponding 95% CIs.

**Results:**

The search identified 8106 studies, of which data were extracted from 589 eligible studies reporting outcomes of 616 708 women from 51 countries. The pooled prevalence of perinatal depression across all studies was 24.7% (95% CI, 23.7%-25.6%). The prevalence of perinatal depression varied slightly by country income status. The highest prevalence was found in lower-middle–income countries, with a pooled prevalence of 25.5% (95% CI, 23.8%-27.1%; 197 studies from 23 countries including 212 103 individuals). In upper-middle–income countries, the pooled prevalence was 24.7% (95% CI, 23.6%-25.9%; 344 studies from 21 countries including 364 103 individuals) and in low-income countries, the pooled prevalence was 20.7% (95% CI, 18.4%-23.0%; 50 studies from 7 countries including 40 502 individuals). The East Asia and the Pacific region had the lowest prevalence of perinatal depression at 21.4% (95% CI, 19.8%-23.1%) and was significantly increased in the Middle East and North Africa at 31.5% (95% CI, 26.9%-36.2%; between-group comparison: *P* < .001). In subgroup analyses, the highest prevalence of perinatal depression was found among women who experienced intimate partner violence, at 38.9% (95% CI, 34.1%-43.6%). revalence of depression was also high among women with HIV (35.1% [95% CI, 29.6%-40.6%]) and those who had experienced a natural disaster (34.8% [95% CI, 29.4%-40.2%]).

**Conclusions and Relevance:**

This meta-analysis found that depression was common in low- and middle-income countries, affecting 1 in 4 perinatal women. Accurate estimates of the prevalence of perinatal depression in low- and middle-income countries are essential in informing policy, allocating scarce resources, and directing further research to improve outcomes for women, infants, and families.

## Key Points

**Question:**

What is the prevalence of depression among individuals in low- and middle-income countries during pregnancy and up to 1 year after birth?

**Findings:**

In this systematic review and meta-analysis of 589 studies and outcomes of 616 708 women from 51 countries, depression was reported for 1 in 4 women (24.7%). Intimate partner violence, having HIV, and the COVID-19 pandemic were all factors associated with increased prevalence of depression in pregnant and postpartum patients.

**Meaning:**

Depression among individuals during pregnancy and up to 1 year post partum in low- and middle-income countries is common and urgently requires action to improve health outcomes for women and infants.

## Teaser

This systematic review and meta-analysis assesses the prevalence of depression among individuals living in low- and middle-income countries during pregnancy and up 1 year post partum.

## Introduction

Depression is among the most common morbidities experienced by individuals during the perinatal period (from conception to 12 months post partum).^1^ While perinatal depression is thought to disproportionally impact individuals in low- and middle-income countries (LMICs), its prevalence in these settings is unclear.^2^

Perinatal depression has significant consequences for individuals and the wider community. It is associated with preterm birth, substance use, continued depression and anxiety, and suicide.^3,4,5^ Individuals who experience HIV, intimate partner violence, and war and conflict are at an increased risk of perinatal depression.^4^ The increased prevalence of these risk factors in LMICs, combined with limited treatment options, place pregnant individuals living in these countries at a higher risk of perinatal depression and the sequelae resulting from depression.^2^

Particularly within low-resource settings, perinatal mental health remains a drastically underemphasized, underresearched, and underresourced area.^3,6^ An accurate estimate of the global prevalence of perinatal depression in LMICs would be a critical step toward advocacy, providing awareness, informing health care policy, and directing scarce resources to reduce its prevalence and improve maternal and infant outcomes. Therefore, the aim of this study was to conduct a systematic review and meta-analysis to determine the prevalence of perinatal depression in LMICs.

## Methods

### Search Strategy and Selection Criteria

We performed a systematic review and meta-analysis to determine the prevalence of perinatal depression in low-, lower-middle–, and upper-middle–income countries. The primary search included terms related to perinatal, mental health disorders, and prevalence and was restricted to studies performed in LMICs as defined by the World Bank (eAppendix 1 in Supplement 1). We limited our study to the perinatal period, which we defined as any time during pregnancy, and up to 12 months post partum. We searched MEDLINE, Embase, PsycINFO, CINAHL, Web of Science, and Cochrane Library databases from database inception to April 15, 2021 (eAppendix 2 in Supplement 1). Studies were included if they measured the prevalence of mental health disorders in a perinatal population using a validated method. Only studies reporting the prevalence of perinatal depression were included. We included cohort studies, cross-sectional studies, baseline data from randomized clinical trials, and prevalence data from controls in case-control studies. Studies that were not written in English were excluded, as were case studies, editorials, guidelines, and review articles.

After removing duplicates, 2 reviewers (A.R.M. and H.G.) independently screened titles, reviewed full texts, and extracted data from eligible studies. Discrepancies were resolved by a third reviewer (R.H.). Covidence systematic review software^7^ was used for data extraction. The following data were extracted: author, year of publication, country of study, study design, study population, country income status, perinatal period (categorized as antenatal, postnatal, or perinatal [combined antenatal and postnatal]), method and tool used to assess depression, total number of study participants, number of patients with depression, the presence of any maternal characteristics associated with an increased risk of depression, adolescence, HIV-positive status, intimate partner violence, and whether patients were experiencing war or conflict or the COVID-19 pandemic.

### Data and Statistical Analysis

For studies measuring depression at multiple times, one antenatal and one postnatal measurement were included and the measurements taken closest to birth were used, excluding the first 2 weeks postnatally (to allow for the postpartum blues).^8^ Prevalence estimates were extracted as raw proportions. Pooled estimates were calculated using a random-effects menta-analysis with a Freeman-Tukey double arcsine transformation to stabilize variance. Point estimates of proportions and their 95% CIs were calculated and displayed in forest plots. For pooled estimates, tau^2^ was used to estimate the between-study variance and the *I*^2^ statistic was used to quantify heterogeneity. High-risk populations were determined a priori and examined in subgroup analyses. These included individuals who, during the perinatal period, experienced war or conflict, natural disaster, or the COVID-19 pandemic, and adolescent individuals, individuals with HIV, and individuals who experience intimate partner violence (subcategorized as physical, psychological, sexual, and not specified). Data were extracted as raw proportions from studies reporting perinatal depression prevalence in these subgroups. Potential sources of heterogeneity were investigated with subgroup and sensitivity analyses, including the exclusions of studies with prevalence estimates less than 5% and more than 60%, and those deemed to be at high risk of bias. Meta-regression was used to examine the differences in prevalence estimates between groups.

An adapted version of the Newcastle-Ottawa Scale^9^ (eAppendix 3 in Supplement 1) was used to assess studies for bias across the domains of selection of participants, comparability of groups, and ascertainment of outcome. Each study was independently assessed for bias by 2 reviewers (A.R.M. and H.G.) and given a score out of 7 (eTable 1 in Supplement 1). Discrepancies were discussed in consultation with a third reviewer (R.H.). Publication bias was examined with funnel plots and Egger test. Stata IC version 17 (StataCorp) was used for the analysis. The study was registered with PROSPERO (CRD42021242901) and reported according to Preferred Reporting Items for Systematic Reviews and Meta-analyses (PRISMA) reporting guideline^10^ (eAppendix 4 in Supplement 1). Two-sided *P* values were statistically significant at less than .05.

## Results

**Figure 1.**
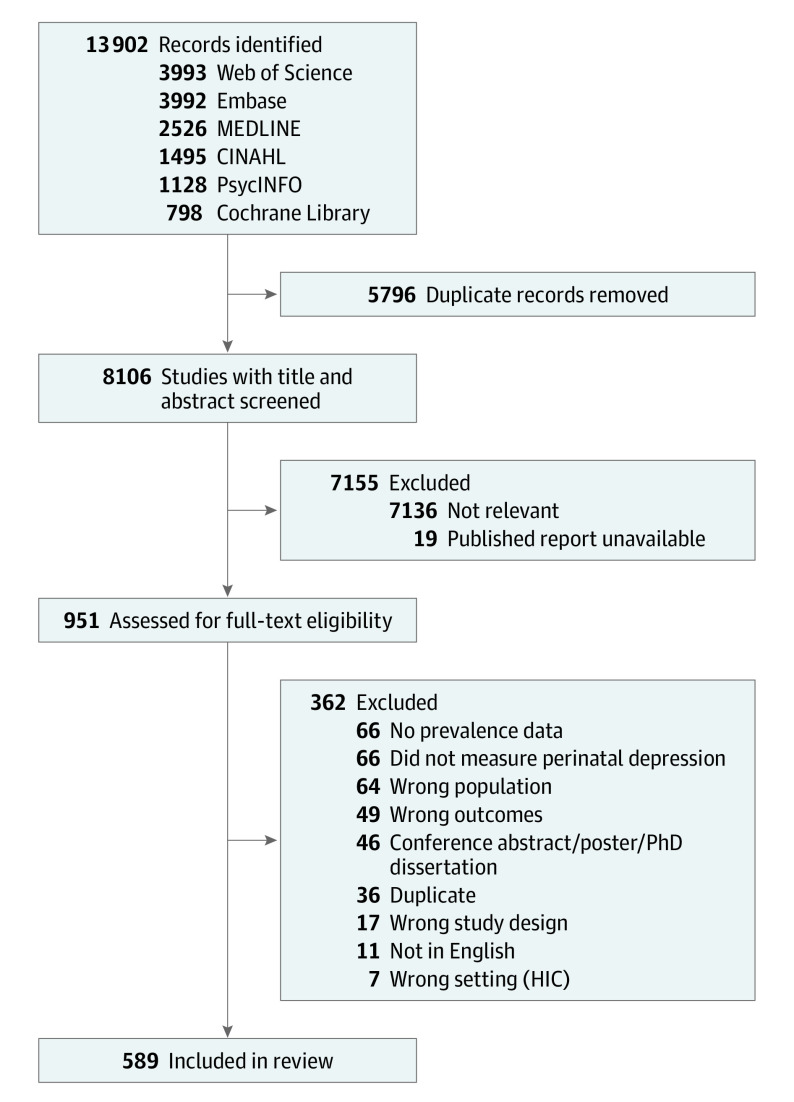
PRISMA Diagram HIC indicates high-income country.

Our search identified 8106 studies. After title and abstract screening, 951 full texts were assessed for eligibility. Of these, 589 met the inclusion criteria (Figure 1). They report outcomes of 616 708 women from 51 countries defined by the World Bank as low, lower-middle, and upper-middle income. Eligible studies were published between 1992 and 2021.

**Figure 2.**
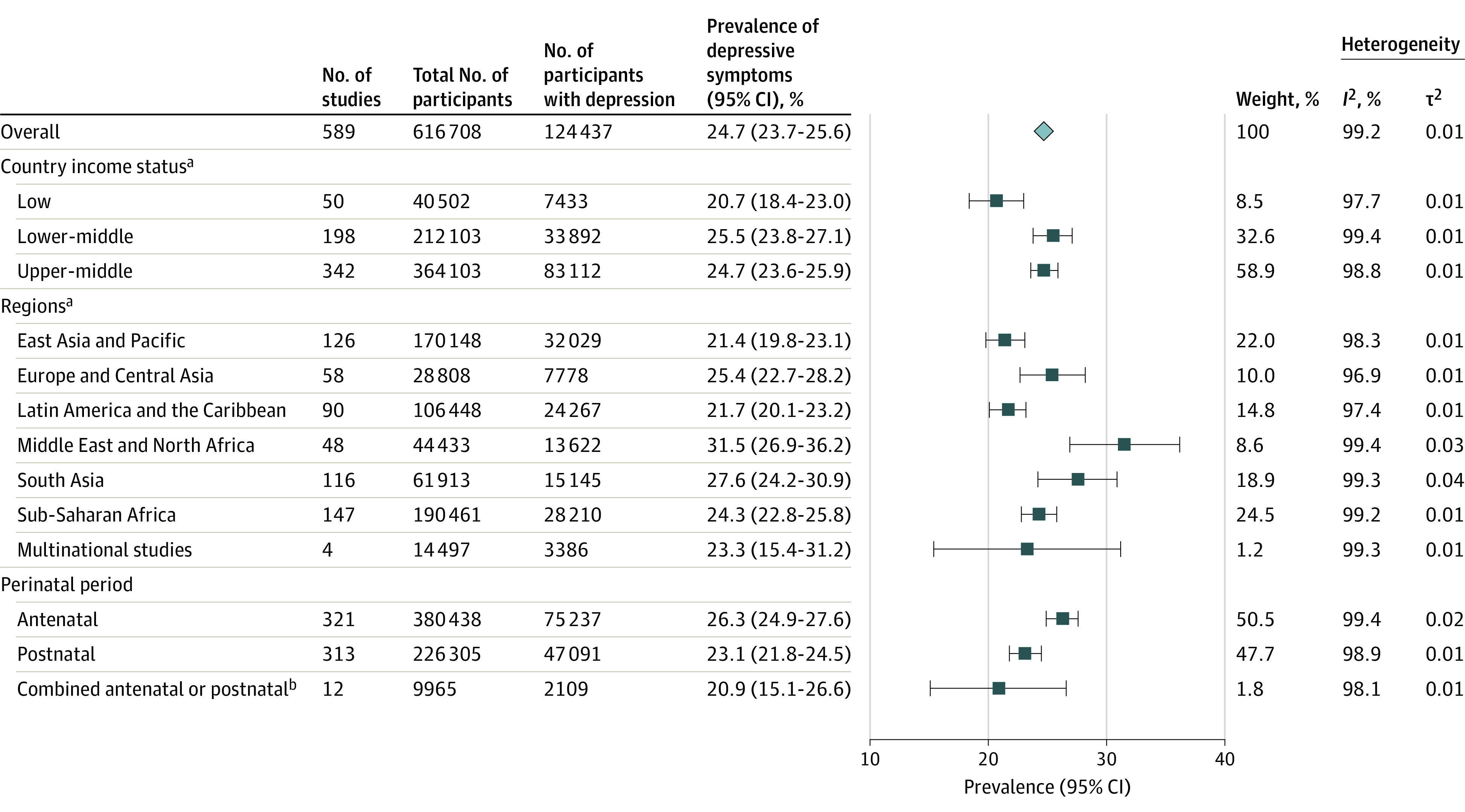
Prevalence of Perinatal Depression in Low-, Lower-Middle–, and Upper-Middle–Income Countries ^a^Defined by the World Bank. ^b^Twelve studies combined antenatal and postnatal women in study sample.

Across 589 included studies (eTable 2 in Supplement 1), the pooled prevalence of perinatal depression was 24.7% (95% CI, 23.7%-25.6%). Depression estimates varied significantly by country income status (*P* < .001). The prevalence was highest in lower-middle–income countries, with a pooled prevalence of 25.5% (95% CI, 23.8%-27.1%; 197 studies from 23 countries including 212 103 individuals). This was followed by upper-middle–income countries, with a pooled prevalence of 24.7% (95% CI, 23.6%-25.9%; 344 studies from 21 countries including 364 103 individuals). The lowest prevalence was found in low-income countries, at 20.7% (95% CI, 18.4%-23.0%; 50 studies from 7 countries including 40 502 individuals) (Figure 2). Among the 50 low-income studies, 48 (96%) were performed in sub-Saharan Africa and 39 (78%) used the Edinburgh Postnatal Depression Scale.

When assessing depression prevalence by region, we found the East Asia and the Pacific region had the lowest prevalence of perinatal depression at 21.4% (95% CI, 19.8%-23.1%; n = 170 148) and was significantly increased in the Middle East and North Africa at 31.5% (95% CI, 26.9%-36.2%; n = 44 433, between-group comparison: *P *< .001) (Figure 2). The Sub-Saharan African region was the most heavily represented, with 25.0% studies (n = 147) coming from this part of Africa.

The pooled prevalence of antenatal depression was 26.3% (95% CI, 24.9%-27.6%; 321 studies with 380 438 individuals), while postnatal depression was significantly lower at 23.1% (95% CI, 21.8%-24.5%; *P* = .001; 313 studies including 226 305 individuals). Twelve studies combined antenatal and postnatal women and reported a prevalence of depression of 20.9% (95% CI, 15.1%-26.6%; n = 9965) (Figure 2).

**Figure 3.**
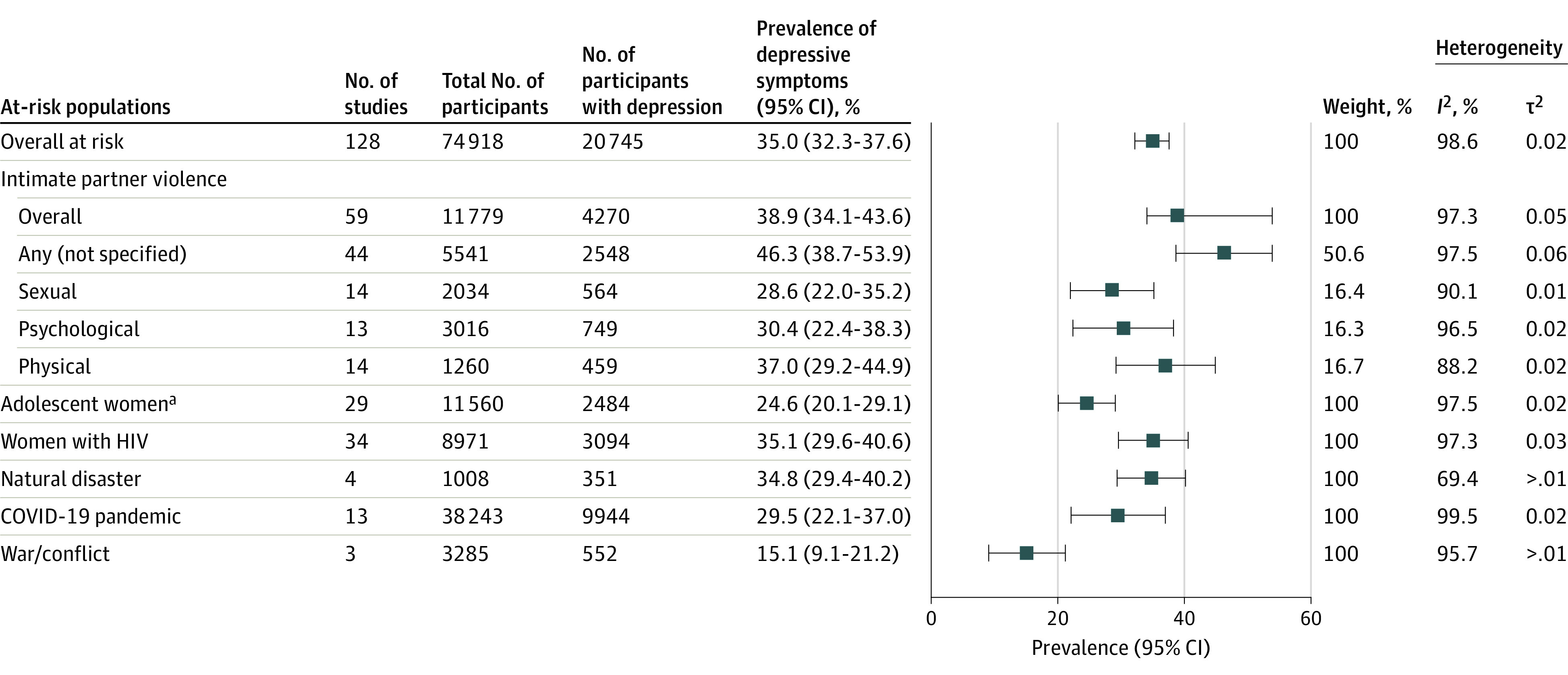
Prevalence of Perinatal Depression in High-Risk Populations in Low-, Lower-Middle–, and Upper-Middle–Income Countries ^a^Younger than 20 years.

We conducted subgroup analyses on populations identified a priori as at risk of developing depression, defined as women who were adolescent, had HIV, or reported intimate partner violence, as well as women who experienced a natural disaster, war and conflict, or the COVID-19 pandemic during the perinatal period. Overall, 128 studies presented data on such at-risk populations, representing 74 918 women (Figure 3). Excluding these women reduced the overall prevalence of depression (23.9% [95% CI, 23.0%-24.9%]). Conversely, women considered at risk had a higher overall prevalence of depression at 35.0% (95% CI, 32.3%-37.6%). The highest prevalence of perinatal depression was found among women who experienced intimate partner violence, at 38.9% (95% CI, 34.1%-43.6%; n = 11 779). Fourteen studies classified the type of intimate partner violence. Among these, the prevalence of depression was 37.0% (95% CI, 29.2%-44.9%) for women who experienced physical intimate partner violence and 28.6% (95% CI, 22.0%-35.2%) among women who experienced sexual intimate partner violence (Figure 3).

Thirteen studies measured the impact of the COVID-19 pandemic on the prevalence of perinatal depression. These studies were conducted in 4 countries (China, Iran, Pakistan, and Turkey), and the estimated prevalence of perinatal depression in this subgroup was 29.5% (95% CI, 22.1%-37.0%; n = 38 243). The prevalence of depression was also higher among women with HIV (35.1% [95% CI, 29.6%-40.6%]; n = 8971) and in women who had experienced a natural disaster (34.8% [95% CI, 29.4%-40.2%]; n = 1008). Adolescent women were found to have a similar prevalence of perinatal depression to the total population (24.6% [95% CI, 20.1%-29.1%]; n = 11 560) (Figure 3).

The prevalence of perinatal depression also varied by the setting. Of the 589 studies, 256 (43.5%) were undertaken in a community or primary health care setting, 245 (41.6%) in a hospital setting, 51 (8.7%) were performed across multiple settings, and 37 (6.3%) were national or statewide database (eTable 3 in Supplement 1). Prevalence estimates were highest among studies performed in teaching hospitals (26.4% [95% CI, 23.6%-29.2%]) and lowest among other hospital settings (22.5% [95% CI, 20.9%-24.2%]).

Prevalence estimates also varied by the tool used to measure depression. Fifty-one studies used structured diagnostic interviews, while 537 studies used a self-reported screening tool, and 1 study used a combination of both (eTables 3-5 in Supplement 1). Prevalence estimates were higher in studies that used self-report screening tools (25.3% [95% CI, 24.3%-26.3%]; n = 598 054) compared with diagnostic interview (17.8% [95% CI, 15.4%-20.1%]; *P *<* *.001; n = 18 123). In total, 28 different instruments were used to diagnose or screen for perinatal depression. The Edinburgh Postnatal Depression Scale was the predominant screening tool used, used in 65.2% (384 of 589) of all studies. The Mini International Neuropsychiatric Interview was the most used interview approach (21 of 589 [3.6%]). Additionally, 387 (65.7%) used a locally validated tool, mainly a local form of the Edinburgh Postnatal Depression Scale. Compared with studies that did not use a locally validated tool, those that did had a significantly lower point prevalence of perinatal depression (26.7% [95% CI, 24.7%-28.7%]) vs 23.7% [95% CI, 22.6%-24.7%]; *P = *.005; eTable 3 in Supplement 1).

Most studies were assessed as being of moderate methodological quality on an adapted version of the Newcastle Ottawa Scale. Studies varied considerably in study methodology, sampled populations, instruments, and cutoff values of the screening tools used. A total of 160 studies were assessed as being at high risk of bias, scoring less than 5 of 7 on the modified Newcastle Ottawa Scale. Representativeness of the exposed cohort, assessment of outcome, and adequacy of cohort follow-up were areas most frequently identified as being at higher risk of bias within included studies (eTable 1 in Supplement 1).

There was significant between-study heterogeneity. We investigated this by performing sensitivity analyses where we stratified by country income status, region, perinatal period, and method used to identify depression. In these sensitivity analyses, we excluded studies with a prevalence of either less than 5% or more than 60%. This removed 42 studies from the analysis but only had a marginal effect on the point estimate. The pooled prevalence estimate from the remaining studies was 23.4% (95% CI, 22.6%-24.2%). Furthermore, excluding 160 studies determined to be at higher risk of bias (scoring less than 5 of 7 on our modified version of the Newcastle Ottawa Scale) did not alter the overall pooled prevalence (eTable 3 in Supplement 1). We generated a funnel plot and used Egger test to examine publication and small study bias (eFigure 1 in Supplement 1). Visual inspection of the funnel plot suggested that small studies were missing and that some publication bias was present; this was confirmed by Egger test (*P* < .001). We explored this further by examining funnel plots by subgroup (eFigures 2-13 in Supplement 1). This demonstrated that publication bias was present in most subgroups.

## Discussion

This systematic review and meta-analysis measured the prevalence of perinatal depression in low-, lower-middle–, and upper-middle–income counties. We found that depression is common during pregnancy and up to 12 months after birth, as it affects 1 in 4 individuals who live in LMICs. There was variation in prevalence estimates between country income status levels and between regions, with the Middle East and North Africa found to have the highest prevalence of perinatal depression. These findings provide a clearer picture of the burden of perinatal depression among individuals in LMICs.

Perinatal depression has been the focus of considerable research in high-income countries, where its prevalence is estimated to be between 10 and 20%.^11,12,13^ In LMICs, however, perinatal depression has received substantially less attention. In these settings, previous prevalence estimates have ranged from 13% to 50%.^14,15,16^ Our study found similar results to an earlier meta-analysis^3^ that examined a small number of studies (representing 20 countries) and reported a prevalence of antenatal depression of 25.3%. Intriguingly, within our study, the prevalence of perinatal depression was lowest among studies performed in a low-income setting. Of these studies, most were from countries within sub-Saharan African and used the Edinburgh Postnatal Depression Scale. Previous studies have questioned the validity of the Edinburgh Postnatal Depression Scale within these countries, with reported sensitivities ranging from 56% to 88%.^17,18,19^ Thus, it is plausible that this low prevalence is due to modest local validity and potentially poor cultural applicability, even when translated,^20^ rather than a truly lower prevalence of perinatal depression.

The consequences of depression at any time can be devastating. During the perinatal period particularly, individuals are at increased risk of onset or relapse of depression.^4,21^ Perinatal depression has been reported to increase the risk of preterm birth, stillbirth, and suicide, which is the leading cause of maternal mortality in many high-income countries.^22,23^ The impact perinatal depression has on offspring can be significant. Studies from LMICs have linked depression with earlier cessation of breastfeeding, more frequent episodes of infant diarrhea, and stunting in the child.^3,24^

As noted across our findings, the prevalence of perinatal depression is not fixed. It varies across countries, regions, and settings. Importantly, it can be reduced. Identifying patients at risk followed by effective policy and targeted intervention has been shown to reduce local and national estimates of depression and its sequalae.^25,26,27^ Intimate partner violence, HIV, adolescence, and living in war or conflict zones are risk factors for depression.^3,4^ We examined such at-risk populations in subgroup analyses. Our findings were in concordance with previous research suggesting that individuals with HIV and those who experience intimate partner violence have a higher prevalence of perinatal depression. In contrast, we did not find a higher prevalence of perinatal depression among adolescent women or women living in war or conflict regions. Studies examining the impact of the COVID-19 pandemic were starting to emerge when our search was conducted. The 13 studies that measured perinatal depression during the pandemic found that rates of perinatal depression were potentially increased. If this finding is valid, then a harmful trend of increased rates of perinatal depression is likely to have persisted given the COVID-19 pandemic remains unabated.

Intimate partner violence is a significant public health problem. The World Health Organization^28^ found that the global lifetime prevalence of intimate partner violence among ever-married/partnered women is 26% and is considerably higher in the least developed regions. Previous studies have shown a strong link between intimate partner violence and perinatal depression.^5,8^ Expanding on this, our results demonstrate that in LMICs, all forms of intimate partner violence (physical, sexual, psychological, or not specified) are associated with increased prevalence of perinatal depression. Hence, recognizing perinatal depression as an important consequence of intimate partner violence is critical.

We found a higher proportion of perinatal women were classified as having depressive symptoms using a self-reported screening instrument compared with a diagnostic interview. Over 90% of the studies included in our systematic review and meta-analysis used a self-reported screening instrument to identify the presence of perinatal depression symptoms. The Edinburgh Postnatal Depression Scale was the predominant tool used both antenatally and postnatally. In resource-constrained settings, conducting diagnostic interviews, while the criterion standard for diagnosing depression, is often infeasible.^16^ Further, mental health training, health care system structure, and resource availability vary considerably between countries and settings.^29^ Hence, pragmatically, the availability and consistency that self-reported screening questionnaires offer makes them valuable tools across LMIC settings.

### Strengths and Limitations

The strength of our study lies in the comprehensive search strategy, which yielded a high number of studies measuring prevalence of perinatal depression in LMICs. By including 589 studies, this represents a very large meta-analysis. It is, to our knowledge, the largest number of LMICs represented in any systematic review assessing perinatal depression. Consequently, we have generated very tight confidence intervals, even for many of our subanalyses. Further, we examined prevalence by region and by World Bank–defined country income status to identify any differential associations on estimates.

There are limitations to our study. The main limitations arise from variation in the design and methodological rigor of included studies and the generalizability of sampled populations. Sensitivity analyses excluding studies reporting extremes of prevalence and those determined to be at highest risk of bias did not show a meaningful difference in prevalence estimates. Second, fewer than 50% of all LMICs are represented in our study. While it is possible that this is due, at least in part, to limiting our search to English-language reports, it is still likely to reflect a worrying lack of studies reporting the incidence of perinatal depression prevalence in many LMICs. This inequity in research between countries around the world is well documented.^30^ Notwithstanding this, we are confident that our study includes the best estimates of the burden of perinatal depression in LMICs across the globe.

Perinatal depression has an important impact on maternal and infant morbidity and mortality rates.^2^ Identification and treatment of individuals experiencing perinatal depression in LMICs is imperative to improve health outcomes in these regions. This is echoed in the Sustainable Development Goals that emphasize the need to reduce maternal mortality and to promote mental health.^31^ The lack of local evidence on the status of perinatal mental health in many LMICs has severe implications for practice and policy.^32^ The prevalence estimates of this study serve to provide evidence of the magnitude of the burden perinatal depression has on individuals in LMICs and to demonstrate the urgent need to recognize perinatal depression as a public health priority globally.

## Conclusions

In this meta-analysis, perinatal depression in LMICs was common. One quarter of individuals living in LMICs reported experiencing depression during and within a year following pregnancy. Perinatal depression is associated with a multitude of poor outcomes for women and their infants. Combating perinatal depression in LMICs will require a multifaceted approach. However, our estimates of its prevalence are an important first step in shining a light on perinatal depression in LMICs: to improve outcomes for women and their children and reduce the inequities that exist between LMICs and high-income countries.

## Notes

Supplement 1. eAppendix 1. World Bank Country Income Classification List 2020 eAppendix 2. Systematic Search Example eAppendix 3. Newcastle-Ottawa Scale (modified version) eAppendix 4. PRISMA 2020 Checklist eTable 1. Risk of bias assessment eTable 2. All included studies and characteristics eTable 3. Prevalence of perinatal depression by method of assessment, local validation, study design, setting and risk of bias score eTable 4. Prevalence of perinatal depression by the three most commonly used self-reported screening instruments eTable 5. Prevalence of perinatal depression with the three most commonly used diagnostic interviews eFigure 1. Funnel plot and Egger's test: all studies eFigure 2. Funnel plot and Egger's test: low-income countries eFigure 3. Funnel plot and Egger's test: lower-middle income countries eFigure 4. Funnel plot and Egger's test: upper-middle income countries eFigure 5. Funnel plot and Egger's test: East Asia and the Pacific region eFigure 6. Funnel plot and Egger's test: Europe and Central Asia region eFigure 7. Funnel plot and Egger's test: Latin America and the Caribbean region eFigure 8. Funnel plot and Egger's test: Middle-East and North Africa region eFigure 9. Funnel plot and Egger's test: South Asia region eFigure 10. Funnel plot and Egger's test: Sub-Saharan region eFigure 11. Funnel plot and Egger's test: antenatal period eFigure 12. Funnel plot and Egger's test: postnatal period eFigure 13. Funnel plot and Egger's test: perinatal (antenatal and postnatal combined) period eReferences

Supplement 2. Data sharing statement
