# Supplementary material for: Prevalence of Perinatal Depression in Low- and Middle-Income Countries: A Systematic Review and Meta-analysis
Source: JAMA Psychiatry. 2023 Mar 8;80(5):425–31. doi: 10.1001/jamapsychiatry.2023.0069 (PMC9996459; doi:10.1001/jamapsychiatry.2023.0069)
Supplement: Supplement 1. — eAppendix 1. World Bank Country Income Classification List 2020 eAppendix 2. Systematic Search Example eAppendix 3. Newcastle-Ottawa Scale (modified version) eAppendix 4. PRISMA 2020 Checklist eTable 1. Risk of bias assessment eTable 2. All included studies and characteristics eTable 3. Prevalence of perinatal depression by method of assessment, local validation, study design, setting and risk of bias score eTable 4. Prevalence of perinatal depression by the three most commonly used self-reported screening instruments eTable 5. Prevalence of perinatal depression with the three most commonly used diagnostic interviews eFigure 1. Funnel plot and Egger's test: all studies eFigure 2. Funnel plot and Egger's test: low-income countries eFigure 3. Funnel plot and Egger's test: lower-middle income countries eFigure 4. Funnel plot and Egger's test: upper-middle income countries eFigure 5. Funnel plot and Egger's test: East Asia and the Pacific region eFigure 6. Funnel plot and Egger's test: Europe and Central Asia region eFigure 7. Funnel plot and Egger's test: Latin America and the Caribbean region eFigure 8. Funnel plot and Egger's test: Middle-East and North Africa region eFigure 9. Funnel plot and Egger's test: South Asia region eFigure 10. Funnel plot and Egger's test: Sub-Saharan region eFigure 11. Funnel plot and Egger's test: antenatal period eFigure 12. Funnel plot and Egger's test: postnatal period eFigure 13. Funnel plot and Egger's test: perinatal (antenatal and postnatal combined) period eReferences [file jamapsychiatry-e230069-s001.pdf]

Supplemental Online Content

Roddy Mitchell A, Gordon H, Lindquist A, et al. Prevalence of perinatal depression in low- and middle-income countries: a systematic review and meta-analysis. *JAMA Psychiatry*. Published online March 8, 2023. doi:10.1001/jamapsychiatry.2023.0069

eAppendix 1. World Bank Country Income Classification List 2020
eAppendix 2. Systematic Search Example
eAppendix 3. Newcastle-Ottawa Scale (modified version)
eAppendix 4. PRISMA 2020 Checklist
eTable 1. Risk of bias assessment
eTable 2. All included studies and characteristics
eTable 3. Prevalence of perinatal depression by method of assessment, local validation, study design, setting and risk of bias score
eTable 4. Prevalence of perinatal depression by the three most commonly used self-reported screening instruments
eTable 5. Prevalence of perinatal depression with the three most commonly used diagnostic interviews
eFigure 1. Funnel plot and Egger's test: all studies
eFigure 2. Funnel plot and Egger's test: low-income countries
eFigure 3. Funnel plot and Egger's test: lower-middle income countries
eFigure 4. Funnel plot and Egger's test: upper-middle income countries
eFigure 5. Funnel plot and Egger's test: East Asia and the Pacific region
eFigure 6. Funnel plot and Egger's test: Europe and Central Asia region
eFigure 7. Funnel plot and Egger's test: Latin America and the Caribbean region
eFigure 8. Funnel plot and Egger's test: Middle-East and North Africa region
eFigure 9. Funnel plot and Egger's test: South Asia region
eFigure 10. Funnel plot and Egger's test: Sub-Saharan region
eFigure 11. Funnel plot and Egger's test: antenatal period
eFigure 12. Funnel plot and Egger's test: postnatal period
eFigure 13. Funnel plot and Egger's test: perinatal (antenatal and postnatal combined) period
eReferences

This supplemental material has been provided by the authors to give readers additional information about their work.

© 2023 Mitchell AR et al. *JAMA Psychiatry.*

eAppendix 1: World Bank Country Income Classification List 2020

Low-Income Countries

Afghanistan
Burkina Faso
Burundi
Central African Republic
Chad
Congo, Democratic Republic
Eritrea
Ethiopia
Gambia, The
Guinea
Guinea-Bissau
Haiti
Korea, Democratic People's Republic
Liberia
Madagascar
Malawi
Mali
Mozambique
Niger
Rwanda
Sierra Leone
Somalia
South Sudan
Sudan
Syrian Arab Republic
Tajikistan
Togo
Uganda
Yemen, Republic

Lower-Middle Income Countries

Angola
Algeria
Bangladesh
Benin
Bhutan
Bolivia
Cabo Verde
Cambodia
Cameroon
Comoros
Congo, Republic
Côte d'Ivoire
Djibouti
Egypt, Arab Republic
El Salvador
Eswatini
Ghana
Honduras
India
Kenya
Kiribati
Kyrgyz Republic
Lao PDR
Lesotho
Mauritania
Micronesia, Federated States
Moldova
Mongolia
Morocco
Myanmar
Nepal
Nicaragua
Nigeria
Pakistan
Papua New Guinea
Philippines
São Tomé and Principe
Senegal
Solomon Islands
Sri Lanka
Tanzania
Timor-Leste
Tunisia
Ukraine
Uzbekistan
Vanuatu
Vietnam
West Bank and Gaza
Zambia
Zimbabwe

© 2023 Mitchell AR et al. *JAMA Psychiatry.*

Upper-Middle Income Countries

Albania
American Samoa
Argentina
Armenia
Azerbaijan
Belarus
Belize
Bosnia and Herzegovina
Botswana
Brazil
Bulgaria
China
Colombia
Costa Rica
Cuba
Dominica
Dominican Republic
Equatorial Guinea
Ecuador
Fiji
Gabon
Georgia
Grenada
Guatemala
Guyana
Indonesia
Iran, Islamic Republic
Iraq
Jamaica
Jordan
Kazakhstan
Kosovo
Lebanon
Libya
Malaysia
Maldives
Marshall Islands
Mexico
Montenegro
Namibia
North Macedonia
Paraguay
Peru
Russian Federation
Samoa
Serbia
South Africa
St. Lucia
St. Vincent and the Grenadines
Suriname
Thailand
Tonga
Turkey
Turkmenistan
Tuvalu
Venezuela, Republic Bolivarian

https://datahelpdesk.worldbank.org/knowledgebase/articles/906519-world-bank-country-and-lending-groups

© 2023 Mitchell AR et al. *JAMA Psychiatry.*

eAppendix 2: Systematic Search Example

MEDLINE Ovid

Order	Search terms
1	Perinatal.mp.
2	(Postnat* OR postpartum).mp.
3	(Pregnan* OR antenat*).mp.
4	1 OR 2 OR 3

Order	Search terms
5	(Mental illness*).mp.
6	(Mental health).mp.
7	(Mental disorder*).mp
8	Psychiatr*.mp.
9	Depression.mp.
10	Anxiety.mp.
11	Bipolar.mp.
12	Schizophreni*.mp.
13	Psycho?i*.mp.
14	5 OR 6 OR 7 OR 8 OR 9 OR 10 OR 11 OR 12 OR 13

Order	Search terms
15	Factor*.mp
16	Determinant*.mp
17	Prevalence.mp
18	Predictor*.mp
19	Rate*.mp
20	Risk*.mp
21	Incidence*.mp
22	15 OR 16 OR 17 OR 18 OR 19 OR 20 OR 21

Order	Search terms
23	EPOC LMIC filter (2020 v.4)
24	4 AND 14 AND 22 AND 23
25	Limit 24 to English language

EPOC LMIC filters 2020 (v.4)
Based on the World Bank list of economies 2019:

© 2023 Mitchell AR et al. *JAMA Psychiatry.*

Filters are based on the World Bank list of countries (2019), classified as low-income, lower-middle-income or upper-middle-income economies:
https://datahelpdesk.worldbank.org/knowledgebase/articles/906519-world-bank-country-and-lending-groups

LMIC COUNTRY NAMES & GENERAL LMIC TERMS
MEDLINE (Ovid)

(**afghanistan** OR **albania** OR **algeria** OR **american samoa** OR **angola** OR **"antigua and barbuda"** OR antigua OR barbuda OR **argentina** OR **armenia** OR armenian OR **aruba** OR **azerbaijan** OR **bahrain** OR **bangladesh** OR **barbados** OR **republic of belarus** OR belarus OR byelarus OR belorussia OR byelorussian OR **belize** OR british honduras OR **benin** OR dahomey OR **bhutan** OR **bolivia** OR **"bosnia and herzegovina"** OR bosnia OR herzegovina OR **botswana** OR bechuanaland OR **brazil** OR brasil OR **bulgaria** OR **burkina faso** OR burkina fasso OR upper volta OR **burundi** OR urundi OR **cabo verde** OR cape verde OR **cambodia** OR kampuchea OR khmer republic OR **cameroon** OR cameron OR cameroun OR **central african republic** OR ubangi shari OR **chad** OR **chile** OR **china** OR **colombia** OR **comoros** OR comoro islands OR iles comores OR mayotte OR **democratic republic of the congo** OR democratic republic congo OR **congo** OR zaire OR **costa rica** OR **"cote d'ivoire"** OR "cote d' ivoire" OR cote divoire OR cote d ivoire OR ivory coast OR **croatia** OR **cuba** OR **cyprus** OR **czech republic** OR czechoslovakia OR **djibouti** OR french somaliland OR **dominica** OR **dominican republic** OR **ecuador** OR **egypt** OR united arab republic OR **el salvador** OR **equatorial guinea** OR spanish guinea OR **eritrea** OR **estonia** OR eswatini OR **swaziland** OR **ethiopia** OR **fiji** OR **gabon** OR gabonese republic OR **gambia** OR **"georgia (republic)"** OR georgian OR **ghana** OR gold coast OR **gibraltar** OR **greece** OR **grenada** OR **guam** OR **guatemala** OR **guinea** OR **guinea bissau** OR **guyana** OR british guiana OR **haiti** OR hispaniola OR **honduras** OR **hungary** OR **india** OR **indonesia** OR timor OR **iran** OR **iraq** OR isle of man OR **jamaica** OR **jordan** OR **kazakhstan** OR kazakh OR **kenya** OR "**democratic people's republic of korea"** OR **republic of korea** OR north korea OR south korea OR korea OR **kosovo** OR **kyrgyzstan** OR kirghizia OR kirgizstan OR kyrgyz republic OR kirghiz OR **laos** OR lao pdr OR "lao people's democratic republic" OR **latvia** OR **lebanon** OR lebanese republic OR **lesotho** OR basutoland OR **liberia** OR **libya** OR libyan arab jamahiriya OR **lithuania** OR **macau** OR macao OR **republic of north macedonia** OR macedonia OR **madagascar** OR malagasy republic OR **malawi** OR nyasaland OR **malaysia** OR malay federation OR malaya federation

© 2023 Mitchell AR et al. *JAMA Psychiatry.*

OR maldives OR **indian ocean islands** OR indian ocean OR **mali** OR **malta** OR **micronesia** OR federated states of micronesia OR kiribati OR marshall islands OR nauru OR northern mariana islands OR **palau** OR tuvalu OR **mauritania** OR **mauritius** OR **mexico** OR **moldova** OR moldovian OR **mongolia** OR **montenegro** OR **morocco** OR ifni OR **mozambique** OR portuguese east africa OR **myanmar** OR burma OR **namibia** OR **nepal** OR **netherlands antilles** OR **nicaragua** OR **niger** OR **nigeria** OR **oman** OR muscat OR **pakistan** OR **panama** OR **papua new guinea** OR new guinea OR **paraguay** OR **peru** OR **philippines** OR philipines OR phillipines OR phillippines OR **poland** OR "polish people's republic" OR **portugal** OR portuguese republic OR **puerto rico** OR **romania** OR **russia** OR russian federation OR ussr OR soviet union OR union of soviet socialist republics OR **rwanda** OR ruanda OR **samoa** OR pacific islands OR polynesia OR samoan islands OR navigator island OR navigator islands OR **"sao tome and principe"** OR **saudi arabia** OR **senegal** OR **serbia** OR **seychelles** OR **sierra leone** OR **slovakia** OR slovak republic OR **slovenia** OR **melanesia** OR solomon island OR solomon islands OR norfolk island OR norfolk islands OR **somalia** OR **south africa** OR **south sudan** OR **sri lanka** OR ceylon OR **"saint kitts and nevis"** OR "st. kitts and nevis" OR **saint lucia** OR "st. lucia" OR **"saint vincent and the grenadines"** OR saint vincent OR "st. vincent" OR grenadines OR **sudan** OR **suriname** OR surinam OR dutch guiana OR netherlands guiana OR **syria** OR syrian arab republic OR **tajikistan** OR tadjikistan OR tadzhikistan OR tadzhik OR **tanzania** OR tanganyika OR **thailand** OR siam OR **timor leste** OR east timor OR **togo** OR togolese republic OR **tonga** OR **"trinidad and tobago"** OR trinidad OR tobago OR **tunisia** OR **turkey** OR **turkmenistan** OR turkmen OR **uganda** OR **ukraine** OR **uruguay** OR **uzbekistan** OR uzbek OR **vanuatu** OR new hebrides OR **venezuela** OR **vietnam** OR viet nam OR **middle east** OR west bank OR gaza OR palestine OR **yemen** OR **yugoslavia** OR **zambia** OR **zimbabwe** OR northern rhodesia OR global south OR **africa south of the sahara** OR sub-saharan africa OR subsaharan africa OR **africa, central** OR central africa OR **africa, northern** OR north africa OR northern africa OR magreb OR maghrib OR sahara OR **africa, southern** OR southern africa OR **africa, eastern** OR east africa OR eastern africa OR **africa, western** OR west africa OR western africa OR **west indies** OR **indian ocean islands** OR **caribbean** OR **central america** OR **latin america** OR "south and central america" OR **south america** OR **asia, central** OR central asia OR **asia, northern** OR north asia OR northern asia OR **asia, southeastern** OR southeastern asia OR south eastern asia OR southeast asia OR south east asia OR **asia, western** OR western asia OR **europe, eastern** OR east europe OR eastern europe OR developing country OR **developing countries** OR

© 2023 Mitchell AR et al. *JAMA Psychiatry.*

developing nation? OR developing population? OR developing world OR less developed countr* OR less developed nation? OR less developed population? OR less developed world OR lesser developed countr* OR lesser developed nation? OR lesser developed population? OR lesser developed world OR under developed countr* OR under developed nation? OR under developed population? OR under developed world OR underdeveloped countr* OR underdeveloped nation? OR underdeveloped population? OR underdeveloped world OR middle income countr* OR middle income nation? OR middle income population? OR low income countr* OR low income nation? OR low income population? OR lower income countr* OR lower income nation? OR lower income population? OR underserved countr* OR underserved nation? OR underserved population? OR underserved world OR under served countr* OR under served nation? OR under served population? OR under served world OR deprived countr* OR deprived nation? OR deprived population? OR deprived world OR poor countr* OR poor nation? OR poor population? OR poor world OR poorer countr* OR poorer nation? OR poorer population? OR poorer world OR developing econom* OR less developed econom* OR lesser developed econom* OR under developed econom* OR underdeveloped econom* OR middle income econom* OR low income econom* OR lower income econom* OR low gdp OR low gnp OR low gross domestic OR low gross national OR lower gdp OR lower gnp OR lower gross domestic OR lower gross national OR lmic OR lmics OR third world OR lami countr* OR transitional countr* OR emerging economies OR emerging nation?)**.ti,ab,sh,kf.**

© 2023 Mitchell AR et al. *JAMA Psychiatry.*

eAppendix 3: Newcastle-Ottawa Scale (modified version)

Selection

- Representativeness of the exposed cohort
  1. Truly/somewhat representative of the average perinatal woman in the community
  0. Selected group of perinatal women/no description of the derivation of the cohort

- Selection of the non-exposed cohort
  1. Drawn from the same community as the expose cohort
  0. Drawn from a different source/no description of the derivation of the non-exposed cohort

- Ascertainment of the exposure
  1. Secure record
  0. Self-reported/no description

Comparability

- Comparability of the cohorts on the basis of the design or analysis
  1. Study controls for important factors so that exposed and non-exposed are comparable
  0. Exposed and non-exposed differ by important factor/no description of comparability

Outcome

- Ascertainment of outcome
  1. Diagnostic interview
  0. Self-reported/no description

- Was follow-up long enough for outcomes to occur
  1. Yes (antenatal ≥ 6 weeks' gestation, postnatal ≥ 2 weeks postpartum)
  0. No

- Adequacy of follow-up of cohort
  1. Complete follow-up of participants/≥80% follow-up of participants
  0. <80% follow-up of participants/no statement

TOTAL score out of 7

© 2023 Mitchell AR et al. *JAMA Psychiatry.*

eTable 1: Risk of bias assessment

NOS domain	Low risk of bias n	Low risk of bias %	High risk of bias n	High risk of bias %	Unclear n	Unclear %
Representativeness of exposed cohort	302	51.3	183	31.1	104	17.6
Selection of the non exposed cohort	571	96.9	5	0.8	13	2.2
Ascertainment of exposure	571	96.9	16	2.7	2	0.3
Comparability of cohorts on the basis of the design or analysis	549	93.2	12	2.0	28	4.8
Ascertainment of outcome	46	7.8	543	92.2	0	0
Was follow-up long enough for outcome to occur	435	73.9	56	9.5	98	16.6
Adequacy of follow-up of cohorts	461	78.3	112	19.0	16	2.7

© 2023 Mitchell AR et al. *JAMA Psychiatry*.

eAppendix 4:

PRISMA 2020 Checklist

Section and Topic	Item #	Checklist item	Location where item is reported
**TITLE**			
Title	1	Identify the report as a systematic review.	Page 1
**ABSTRACT**			
Abstract	2	See the PRISMA 2020 for Abstracts checklist.	Page 2
**INTRODUCTION**			
Rationale	3	Describe the rationale for the review in the context of existing knowledge.	Page 5
Objectives	4	Provide an explicit statement of the objective(s) or question(s) the review addresses.	Page 5
**METHODS**			
Eligibility criteria	5	Specify the inclusion and exclusion criteria for the review and how studies were grouped for the syntheses.	Page 6
Information sources	6	Specify all databases, registers, websites, organisations, reference lists and other sources searched or consulted to identify studies. Specify the date when each source was last searched or consulted.	Page 6
Search strategy	7	Present the full search strategies for all databases, registers and websites, including any filters and limits used.	Appendix 2
Selection process	8	Specify the methods used to decide whether a study met the inclusion criteria of the review, including how many reviewers screened each record and each report retrieved, whether they worked independently, and if applicable, details of automation tools used in the process.	Page 6
Data collection process	9	Specify the methods used to collect data from reports, including how many reviewers collected data from each report, whether they worked independently, any processes for obtaining or confirming data from study investigators, and if applicable, details of automation tools used in the process.	Page 6
Data items	10a	List and define all outcomes for which data were sought. Specify whether all results that were compatible with each outcome domain in each study were sought (e.g. for all measures, time points, analyses), and if not, the methods used to decide which results to collect.	Page 6
	10b	List and define all other variables for which data were sought (e.g. participant and intervention characteristics, funding sources). Describe any assumptions made about any missing or unclear information.	Pages 6 & 7
Study risk of	11	Specify the methods used to assess risk of bias in the included studies, including details of the tool(s) used, how many	Page 7 &

© 2023 Mitchell AR et al. *JAMA Psychiatry*.

Section and Topic	Item #	Checklist item	Location where item is reported
bias assessment		reviewers assessed each study and whether they worked independently, and if applicable, details of automation tools used in the process.	Appendix 3
Effect measures	12	Specify for each outcome the effect measure(s) (e.g. risk ratio, mean difference) used in the synthesis or presentation of results.	Page 7
Synthesis methods	13a	Describe the processes used to decide which studies were eligible for each synthesis (e.g. tabulating the study intervention characteristics and comparing against the planned groups for each synthesis (item #5)).	Page 6
	13b	Describe any methods required to prepare the data for presentation or synthesis, such as handling of missing summary statistics, or data conversions.	Page 6
	13c	Describe any methods used to tabulate or visually display results of individual studies and syntheses.	Appendix 6
	13d	Describe any methods used to synthesize results and provide a rationale for the choice(s). If meta-analysis was performed, describe the model(s), method(s) to identify the presence and extent of statistical heterogeneity, and software package(s) used.	Page 7
	13e	Describe any methods used to explore possible causes of heterogeneity among study results (e.g. subgroup analysis, meta-regression).	Page 7
	13f	Describe any sensitivity analyses conducted to assess robustness of the synthesized results.	Page 7
Reporting bias assessment	14	Describe any methods used to assess risk of bias due to missing results in a synthesis (arising from reporting biases).	Page 7 & supplementary
Certainty assessment	15	Describe any methods used to assess certainty (or confidence) in the body of evidence for an outcome.	Page 7
**RESULTS**			
Study selection	16a	Describe the results of the search and selection process, from the number of records identified in the search to the number of studies included in the review, ideally using a flow diagram.	Figure 1
	16b	Cite studies that might appear to meet the inclusion criteria, but which were excluded, and explain why they were excluded.	Figure 1 & supplementary
Study characteristics	17	Cite each included study and present its characteristics.	Supplementary
Risk of bias in studies	18	Present assessments of risk of bias for each included study.	Supplementary
Results of individual studies	19	For all outcomes, present, for each study: (a) summary statistics for each group (where appropriate) and (b) an effect estimate and its precision (e.g. confidence/credible interval), ideally using structured tables or plots.	Tables 1, 2 & supplementary
Results of	20a	For each synthesis, briefly summarise the characteristics and risk of bias among contributing studies.	Tables 1 & 2

© 2023 Mitchell AR et al. *JAMA Psychiatry*.

Section and Topic	Item #	Checklist item	Location where item is reported
syntheses	20b	Present results of all statistical syntheses conducted. If meta-analysis was done, present for each the summary estimate and its precision (e.g. confidence/credible interval) and measures of statistical heterogeneity. If comparing groups, describe the direction of the effect.	Tables 1 & 2
	20c	Present results of all investigations of possible causes of heterogeneity among study results.	Page 10 & supplementary
	20d	Present results of all sensitivity analyses conducted to assess the robustness of the synthesized results.	Page 10 & supplementary
Reporting biases	21	Present assessments of risk of bias due to missing results (arising from reporting biases) for each synthesis assessed.	Supplementary
Certainty of evidence	22	Present assessments of certainty (or confidence) in the body of evidence for each outcome assessed.	Pages 8 – 10
**DISCUSSION**			
Discussion	23a	Provide a general interpretation of the results in the context of other evidence.	Page 11
	23b	Discuss any limitations of the evidence included in the review.	Page 13
	23c	Discuss any limitations of the review processes used.	Page 13
	23d	Discuss implications of the results for practice, policy, and future research.	Pages 13 & 14
**OTHER INFORMATION**			
Registration and protocol	24a	Provide registration information for the review, including register name and registration number, or state that the review was not registered.	Page 7
	24b	Indicate where the review protocol can be accessed, or state that a protocol was not prepared.	Page 7
	24c	Describe and explain any amendments to information provided at registration or in the protocol.	NA
Support	25	Describe sources of financial or non-financial support for the review, and the role of the funders or sponsors in the review.	Page 7
Competing interests	26	Declare any competing interests of review authors.	NA
Availability of data, code and other materials	27	Report which of the following are publicly available and where they can be found: template data collection forms; data extracted from included studies; data used for all analyses; analytic code; any other materials used in the review.	Supplementary

From: Page MJ, McKenzie JE, Bossuyt PM, Boutron I, Hoffmann TC, Mulrow CD, et al. The PRISMA 2020 statement: an updated guideline for reporting systematic reviews. BMJ 2021;372:n71. doi: 10.1136/bmj.n71

For more information, visit: http://www.prisma-statement.org/

© 2023 Mitchell AR et al. *JAMA Psychiatry*.

eTable 2: All included studies and characteristics

Author	Year	Country	Title	Tool used	Cut off value of screening tool	Screening tool locally validated	NOS score
Albania							
Cela et al. [1]	2019	Albania	An evaluation of postpartum depression - a study in Albania 2015 - 2017 period	Edinburgh Postnatal Depression Scale	≥13	no	2
Mece [2]	2014	Albania	Relationship between postpartum depression, infant temperament and childcare stress in Tirana, Albania	Edinburgh Postnatal Depression Scale	Not specified	no	4
Argentina							
Cormick et al. [3]	2021	Argentina	Factors associated with depression during pregnancy in women from a low socioeconomic level: A hierarchical model approach	Edinburgh Postnatal Depression Scale	≥10	no	5
Pham et al. [4]	2018	Argentina	Factors associated with postpartum depression in women from low socioeconomic level in Argentina: A hierarchical model approach	Edinburgh Postnatal Depression Scale	≥10	yes	6
Mathisen et al. [5]	2013	Argentina	Prevalence and risk factors for postpartum depressive symptoms in Argentina: A cross-sectional study	Edinburgh Postnatal Depression Scale	≥10	no	5
Bangladesh							
Tasnim et al. [6]	2021	Bangladesh	Exposure to domestic violence and the risk of developing depression within 6 months postpartum in Bangladesh	Edinburgh Postnatal Depression Scale	≥10	yes	5
Valdes et al. [7]	2021	Bangladesh	Socioeconomic and psychological correlates of postpartum depression at 6 months in Dhaka, Bangladesh	Edinburgh Postnatal Depression Scale	≥10	yes	7
Begum et al. [8]	2020	Bangladesh	Changes in Depressive Symptoms During the Antenatal Period: A Cohort Study from Bangladesh	Edinburgh Postnatal Depression Scale	≥10	yes	6
Islam et al. [9]	2020	Bangladesh	Do Maternal Depression and Self-Esteem Moderate and Mediate the	Edinburgh Postnatal Depression Scale	≥9	no	6

© 2023 Mitchell AR et al. *JAMA Psychiatry*.

			Association Between Intimate Partner Violence After Childbirth and Postpartum Suicidal Ideation?				
Azad et al. [10]	2019	Bangladesh	Prevalence and risk factors of postpartum depression within one year after birth in urban slums of Dhaka, Bangladesh	Edinburgh Postnatal Depression Scale	≥10	yes	5
Sharmin et al. [11]	2019	Bangladesh	Postnatal depression and infant growth in an urban area of Bangladesh	Edinburgh Postnatal Depression Scale	not specified	yes	4
Nasreen et al. [12]	2013	Bangladesh	Impact of maternal depressive symptoms and infant temperament on early infant growth and motor development: results from a population based study in Bangladesh	Edinburgh Postnatal Depression Scale	≥10	yes	5
Edhborg et al. [13]	2011	Bangladesh	Impact of postpartum depressive and anxiety symptoms on mothers' emotional tie to their infants 2-3 months postpartum: A population-based study from rural Bangladesh	Edinburgh Postnatal Depression Scale	≥10	yes	5
Nasreen et al. [14]	2011	Bangladesh	Prevalence and associated factors of depressive and anxiety symptoms during pregnancy: A population based study in rural Bangladesh	Edinburgh Postnatal Depression Scale	≥10	yes	6
Nasreen et al. [15]	2010	Bangladesh	Low birth weight in offspring of women with depressive and anxiety symptoms during pregnancy: results from a population based study in Bangladesh	Edinburgh Postnatal Depression Scale	≥10	yes	6
Gausia et al. [16]	2009	Bangladesh	Antenatal depression and suicidal ideation among rural Bangladeshi women: a community-based study	Edinburgh Postnatal Depression Scale	≥10	yes	5
Gausia et al. [17]	2009	Bangladesh	Magnitude and contributory factors of postnatal depression: a community-based cohort study from a rural subdistrict of Bangladesh	Edinburgh Postnatal Depression Scale	≥10	yes	6

© 2023 Mitchell AR et al. *JAMA Psychiatry*.

Brazil							
Santos et al. [18]	2021	Brazil	Post-partum depression: a cross-sectional study of women enrolled in a conditional cash transfer program in 30 Brazilian cities	Edinburgh Postnatal Depression Scale	≥10	yes	5
Soares et al. [19]	2021	Brazil	Suicide risk and prematurity: A study with pregnant adolescents	Mini International Neuropsychiatric Interview	≥11	yes	5
de Mello et al. [20]	2021	Brazil	Generalized Anxiety Disorder, Depressive Symptoms and the Occurrence of Stressors Events in a Probabilistic Sample of Pregnant Women	Beck Depression Inventory II	≥13	no	4
Ribeiro et al. [21]	2020	Brazil	Childhood trauma and depressive symptoms in pregnant adolescents in Southern Brazil	Beck Depression Inventory	≥13	no	5
Pabon et al. [22]	2020	Brazil	Overall Maternal Morbidity during Pregnancy Identified with the WHO-WOICE Instrument	Patient Health Questionnaire	≥10	yes	6
Netsi et al. [23]	2020	Brazil	Female infants are more susceptible to the effects of maternal antenatal depression; findings from the Pelotas (Brazil) Birth Cohort Study	Edinburgh Postnatal Depression Scale	≥13	yes	6
Avilla et al. [24]	2020	Brazil	Association between maternal satisfaction with breastfeeding and postpartum depression symptoms	Edinburgh Postnatal Depression Scale	≥11	yes	5
Leite et al. [25]	2020	Brazil	Disrespect and abuse towards women during childbirth and postpartum depression: findings from Birth in Brazil Study	Edinburgh Postnatal Depression Scale	≥13	yes	6
Farias-Antunez et al. [26]	2020	Brazil	Maternal mood symptoms in pregnancy and postpartum depression: association with exclusive breastfeeding in a population-based birth cohort	Edinburgh Postnatal Depression Scale	≥10	yes	6
Silveira et al. [27]	2019	Brazil	The association between disrespect and abuse of women during childbirth and postpartum	Edinburgh Postnatal Depression Scale	≥13	yes	5

© 2023 Mitchell AR et al. *JAMA Psychiatry*.

			depression: Findings from the 2015 Pelotas birth cohort study				
Silva et al. [28]	2019	Brazil	Postpartum depression: a case-control study	Edinburgh Postnatal Depression Scale	≥10	yes	4
Callo-Quinte et al. [29]	2019	Brazil	Maternal depression symptoms and use of child health-care services at The Pelotas 2004 Birth Cohort	Edinburgh Postnatal Depression Scale	≥10	yes	6
Araujo et al. [30]	2019	Brazil	Postpartum Depression: Epidemiological Clinical Profile of Patients Attended In a Reference Public Maternity in Salvador-BA	Edinburgh Postnatal Depression Scale	≥10	no	6
Costa et al. [31]	2018	Brazil	Mental disorders in pregnancy and newborn conditions: longitudinal study with pregnant women attended in primary care	Primary Care Evaluation of Mental Disorders	n/a	--	6
Pinto et al. [32]	2017	Brazil	Serum n-3 polyunsaturated fatty acids are inversely associated with longitudinal changes in depressive symptoms during pregnancy	Edinburgh Postnatal Depression Scale	≥11	yes	4
Paskulin et al. [33]	2017	Brazil	Association between dietary patterns and mental disorders in pregnant women in Southern Brazil	Patient Health Questionnaire	not specified	yes	6
Coll et al. [34]	2017	Brazil	Antenatal depressive symptoms among pregnant women: Evidence from a Southern Brazilian population-based cohort study	Edinburgh Postnatal Depression Scale	≥10	yes	5
Faisal-Cury et al. [35]	2017	Brazil	Unplanned pregnancy and risk of maternal depression: secondary data analysis from a prospective pregnancy cohort	Self Reporting Questionnaire	>7	yes	5
Moraes et al. [36]	2016	Brazil	Depressive Symptoms in Pregnancy: The Influence of Social, Psychological and Obstetric Aspects	Hospital Anxiety and Depression Scale	≥12	yes	5
Correa et al. [37]	2016	Brazil	Postpartum depression symptoms among Amazonian and Northeast Brazilian women	Edinburgh Postnatal Depression Scale	≥11	yes	5

© 2023 Mitchell AR et al. *JAMA Psychiatry*.

Hassan et al. [38]	2016	Brazil	Maternal mental health and nutritional status of six-month-old infants	General Health Questionnaire	≥9	yes	6
de Jesus Silva et al. [39]	2016	Brazil	Depression in pregnancy. Prevalence and associated factors	Hospital Anxiety and Depression Scale	≥9	yes	6
Castroe Couto et al. [40]	2016	Brazil	Antenatal depression: Prevalence and risk factor patterns across the gestational period	Mini International Neuropsychiatric Interview	n/a	--	5
de Figueiredo et al. [41]	2015	Brazil	Postpartum depression screening by telephone: a good alternative for public health and research	Edinburgh Postnatal Depression Scale	≥10	yes	3
Ferreira et al. [42]	2015	Brazil	Prevalence of anxiety symptoms and depression in the third gestational trimester	Centre of Epidemiological Studies-Depression	≥16	no	6
Vaz et al. [43]	2014	Brazil	Omega-6 fatty acids and greater likelihood of suicide risk and major depression in early pregnancy	Mini International Neuropsychiatric Interview	n/a	--	4
Teofilo et al. [44]	2014	Brazil	HDL-cholesterol concentration is inversely associated with depressive symptoms during pregnancy: A prospective cohort in Rio De Janeiro, Brazil	Edinburgh Postnatal Depression Scale	≥11	yes	4
Coelho et al. [45]	2014	Brazil	Parental bonding and suicidality in pregnant teenagers: a population-based study in southern Brazil	Mini International Neuropsychiatric Interview	n/a	--	7
Zaconeta et al. [46]	2013	Brazil	Depression with postpartum onset: A prospective cohort study in women undergoing elective cesarean section in Brasilia, Brazil	Edinburgh Postnatal Depression Scale	≥13	yes	5
Takahasi et al. [47]	2013	Brazil	Mental health and physical inactivity during pregnancy: a cross-sectional study nested in the BRISA cohort study	Centre of Epidemiological Studies-Depression	≥22	no	6
Farias et al. [48]	2013	Brazil	Prevalence of psychiatric disorders in the first trimester of pregnancy and factors associated with current suicide risk	Mini International Neuropsychiatric Interview	n/a	--	6

© 2023 Mitchell AR et al. *JAMA Psychiatry*.

Faisal-Cury et al. [49]	2013	Brazil	Temporal relationship between intimate partner violence and postpartum depression in a sample of low income women	Self Reporting Questionnaire	>7	yes	5
da Silva et al. [50]	2012	Brazil	Suicidality and associated factors in pregnant women in Brazil	Hospital Anxiety and Depression Scale	≥8	no	5
Tavares et al. [51]	2012	Brazil	Prevalence of suicide risk and comorbidities in postpartum women in Pelotas	Mini International Neuropsychiatric Interview	n/a	--	7
Silva et al. [52]	2012	Brazil	Sociodemographic risk factors of perinatal depression: a cohort study in the public health care system	Edinburgh Postnatal Depression Scale	≥13	yes	4
Pinheiro et al. [53]	2012	Brazil	Suicidal behavior in pregnant teenagers in southern Brazil: Social, obstetric and psychiatric correlates	Mini International Neuropsychiatric Interview	n/a	--	6
Manzolli et al. [54]	2012	Brazil	Abuse against women, depression, and infant morbidity: a primary care cohort study in Brazil	Primary Care Evaluation of Mental Disorders	n/a	yes	4
Faisal-Cury et al. [55]	2012	Brazil	Coping style and depressive symptomatology during pregnancy in a private setting sample	Beck Depression Inventory	>15	yes	5
da Rocha et al. [56]	2012	Brazil	High dietary ratio of omega-6 to omega-3 polyunsaturated acids during pregnancy and prevalence of post-partum depression	Edinburgh Postnatal Depression Scale	≥11	yes	5
Bottino et al. [57]	2012	Brazil	Reappraising the relationship between maternal age and postpartum depression according to the evolutionary theory: Empirical evidence from a survey in primary health services	Edinburgh Postnatal Depression Scale	>11	yes	5
Melo et al. [58]	2012	Brazil	The prevalence of perinatal depression and its associated factors in two different settings in Brazil	Edinburgh Postnatal Depression Scale	>11	yes	5
Lobato et al. [59]	2012	Brazil	Alcohol misuse among partners: a potential effect modifier in the relationship between physical	Edinburgh Postnatal Depression Scale	≥12	yes	6

© 2023 Mitchell AR et al. *JAMA Psychiatry*.

			intimate partner violence and postpartum depression				
Faisal-Cury et al. [60]	2012	Brazil	Antenatal depression strongly predicts postnatal depression in primary health care	Self Reporting Questionnaire	≥8	yes	6
Pinheiro et al. [61]	2011	Brazil	Chronicity and severity of maternal postpartum depression and infant sleep disorders: a population-based cohort study in southern Brazil	Edinburgh Postnatal Depression Scale	≥13	yes	3
Dias et al. [62]	2011	Brazil	Pregnancy is associated with psychiatric symptoms in a low-income countryside community of Brazil	Mini International Neuropsychiatric Interview	n/a	--	7
Silva et al. [63]	2010	Brazil	Relationship between religious practice, alcohol use, and psychiatric disorders among pregnant women.	Mini International Neuropsychiatric Interview	n/a	--	7
Manzolli et al. [64]	2010	Brazil	Violence and depressive symptoms during pregnancy: a primary care study in Brazil	Primary Care Evaluation of Mental Disorders	≥6	yes	4
Ludermir et al. [65]	2010	Brazil	Violence against women by their intimate partner during pregnancy and postnatal depression: a prospective cohort study	Edinburgh Postnatal Depression Scale	≥12	yes	6
Da Silva et al. [66]	2010	Brazil	Depression during pregnancy in the Brazilian public health care system	Edinburgh Postnatal Depression Scale	≥13	yes	5
Jansen et al. [67]	2010	Brazil	Tobacco smoking and depression during pregnancy	Edinburgh Postnatal Depression Scale	≥13	yes	5
Cantilino et al. [68]	2010	Brazil	Postpartum depression in Recife - Brazil: Prevalence and association with bio-socio-demographic factors	Structured Clinical Interview DSM-IV criteria	n/a	--	5
Zambaldi et al. [69]	2009	Brazil	Postpartum obsessive-compulsive disorder: prevalence and clinical characteristics	Structured Clinical Interview DSM-IV criteria	n/a	--	7
Pereira et al. [70]	2009	Brazil	Depression during pregnancy: prevalence and risk factors among women attending a public health clinic in Rio de Janeiro, Brazil	Composite International Diagnostic Interview	n/a	--	7

© 2023 Mitchell AR et al. *JAMA Psychiatry*.

Matijasevich et al. [71]	2009	Brazil	Differentials and income-related inequalities in maternal depression during the first two years after childbirth: birth cohort studies from Brazil and the UK	Edinburgh Postnatal Depression Scale	≥13	yes	4
Tannous et al. [72]	2008	Brazil	Postnatal depression in Southern Brazil: prevalence and its demographic and socioeconomic determinants	Edinburgh Postnatal Depression Scale	≥13	yes	6
Hasselmann et al. [73]	2008	Brazil	Symptoms of postpartum depression and early interruption of exclusive breastfeeding in the first two months of life	Edinburgh Postnatal Depression Scale	≥12	yes	4
Cantilino et al. [74]	2007	Brazil	Translation, validation and cultural aspects of postpartum depression screening scale in Brazilian Portuguese	Structured Clinical Interview DSM-IV criteria	n/a	--	5
Faisal-Cury et al. [75]	2007	Brazil	Prevalence of anxiety and depression during pregnancy in a private setting sample	Beck Depression Inventory	>15	yes	5
Pinheiro et al. [76]	2006	Brazil	Is paternal postpartum depression associated with maternal postpartum depression? Population-based study in Brazil	Beck Depression Inventory	>9	yes	6
Faisal-Cury et al. [77]	2004	Brazil	Postpartum depression: in relation to life events and patterns of coping	Beck Depression Inventory	>15	yes	5
Da-Silva et al. [78]	1998	Brazil	Prenatal and postnatal depression among low income Brazilian women	Edinburgh Postnatal Depression Scale	≥13	no	4
China							
Zhang et al. [79]	2021	China	Psychobehavioral Responses, Post-Traumatic Stress and Depression in Pregnancy During the Early Phase of COVID-19 Outbreak	Edinburgh Postnatal Depression Scale	≥13	no	4
Yang et al. [80]	2021	China	Social, Cognitive, and eHealth Mechanisms of COVID-19-Related Lockdown and Mandatory Quarantine That Potentially Affect	Patient Health Questionnaire	≥10	no	5

© 2023 Mitchell AR et al. *JAMA Psychiatry*.

			the Mental Health of Pregnant Women in China: Cross-Sectional Survey Study				
Xie et al. [81]	2021	China	Alteration in the psychologic status and family environment of pregnant women before and during the COVID-19 pandemic	Symptom Checklist 90 (Revised)	≥2	no	5
Wang et al. [82]	2021	China	Mental health and preventive behaviour of pregnant women in China during the early phase of the COVID-19 period	Patient Health Questionnaire	≥10	yes	3
Shi et al. [83]	2021	China	Risk factors for postpartum depression in Chinese women: A cross-sectional study at 6 weeks postpartum	Edinburgh Postnatal Depression Scale	≥10	yes	4
Peng et al. [84]	2021	China	Prevalence and risk factors of postpartum depression in China: A hospital-based cross-sectional study	Edinburgh Postnatal Depression Scale	≥10	yes	5
Lin et al. [85]	2021	China	Sleep Conditions Associate with Anxiety and Depression Symptoms among Pregnant Women during the Epidemic of COVID-19 in Shenzhen	Self Rating Depression Scale	≥5	yes	4
Jiang et al. [86]	2021	China	The mental health status and approaches of accessing antenatal care information among pregnant women during COVID-19 epidemic: a cross-sectional study in China	Edinburgh Postnatal Depression Scale	≥10	yes	2
Dong et al. [87]	2021	China	Investigation on the mental health status of pregnant women in China during the Pandemic of COVID-19	Self Rating Depression Scale	≥50	yes	1
Mo et al. [88]	2021	China	Association of Perceived Threat, Negative Emotions, and Self-Efficacy With Mental Health and Personal Protective Behavior Among Chinese Pregnant Women	Patient Health Questionnaire	≥5	yes	4

© 2023 Mitchell AR et al. *JAMA Psychiatry*.

			During the COVID-19 Pandemic: Cross-sectional Survey Study				
Li et al. [89]	2021	China	The prevalence and risk factors of depression in prenatal and postnatal women in China with the outbreak of Corona Virus Disease 2019	Patient Health Questionnaire	>5	yes	3
Huang et al. [90]	2021	China	Family function fully mediates the relationship between social support and perinatal depression in rural Southwest China	Edinburgh Postnatal Depression Scale	≥10	yes	4
Zhou et al. [91]	2020	China	The prevalence of psychiatric symptoms of pregnant and non-pregnant women during the COVID-19 epidemic	Patient Health Questionnaire	≥10	yes	1
Zhang et al. [92]	2020	China	Prevalence of Prenatal Depression Among Pregnant Women and the Importance of Resilience: A Multi-Site Questionnaire-Based Survey in Mainland China	Centre of Epidemiological Studies-Depression	≥16	yes	6
Zhang et al. [93]	2020	China	The Relationship Between Images Posted by New Mothers on WeChat Moments and Postpartum Depression: Cohort Study	Edinburgh Postnatal Depression Scale	≥10	yes	5
Zhang et al. [94]	2020	China	Suicide ideation among pregnant women: The role of different experiences of childhood abuse	Patient Health Questionnaire	≥10	yes	2
Zeng et al. [95]	2020	China	Mental Health Outcomes in Perinatal Women During the Remission Phase of COVID-19 in China	Edinburgh Postnatal Depression Scale	≥13	yes	4
Zheng et al. [96]	2020	China	Association between family functions and antenatal depression symptoms: A cross-sectional study among pregnant women in urban communities of Hengyang city, China	Patient Health Questionnaire	≥10	yes	5
Yu et al. [97]	2020	China	Coping styles in pregnancy, their demographic and psychological	Edinburgh Postnatal Depression Scale	≥10	yes	5

© 2023 Mitchell AR et al. *JAMA Psychiatry*.

			influences, and their association with postpartum depression: A longitudinal study of women in China				
Xiong et al. [98]	2020	China	Prevalence and associated factors of postpartum depression among immigrant women in Guangzhou, China	Edinburgh Postnatal Depression Scale	≥10	yes	5
Wu et al. [99]	2020	China	Perinatal depressive and anxiety symptoms of pregnant women during the coronavirus disease 2019 outbreak in China	Edinburgh Postnatal Depression Scale	≥10	yes	5
Sun et al. [100]	2020	China	Perinatal Depression of Exposed Maternal Women in the COVID-19 Pandemic in Wuhan, China	Edinburgh Postnatal Depression Scale	≥10	yes	4
Yu et al. [101]	2020	China	Prevalence of depression symptoms and its influencing factors among pregnant women in late pregnancy in urban areas of Hengyang City, Hunan Province, China: A cross-sectional study	Patient Health Questionnaire	≥10	no	5
Minglu et al. [102]	2020	China	Influencing factors and correlation of anxiety, psychological stress sources, and psychological capital among women pregnant with a second child in Guangdong and Shandong Province	Self Rating Depression Scale	≥50	yes	5
Min et al. [103]	2020	China	Associations between Maternal and Infant Illness and the Risk of Postpartum Depression in Rural China: A Cross-Sectional Observational Study	Depression Anxiety Stress Scale	≥10	yes	4
Liu et al. [104]	2020	China	Prevalence and Associated Factors of Postpartum Anxiety and Depression Symptoms Among Women in Shanghai, China	Edinburgh Postnatal Depression Scale	≥13	yes	5
Liang et al. [105]	2020	China	Prevalence and factors associated with postpartum depression during	Edinburgh Postnatal Depression Scale	≥10	yes	4

© 2023 Mitchell AR et al. *JAMA Psychiatry*.

			the COVID-19 pandemic among women in Guangzhou, China: a cross-sectional study				
Li et al. [106]	2020	China	The association between symptoms of depression during pregnancy and low birth weight: a prospective study	Edinburgh Postnatal Depression Scale	≥12	yes	5
Li et al. [107]	2020	China	Impact of some social and clinical factors on the development of postpartum depression in Chinese women	Edinburgh Postnatal Depression Scale	≥9	yes	5
Gong et al. [108]	2020	China	Comprehensive intervention during pregnancy based on short message service to prevent or alleviate depression in pregnant women: a quasi-experimental study	Edinburgh Postnatal Depression Scale	>9	yes	4
Guo et al. [109]	2020	China	Comparisons on perinatal depression between the first-child women and the second-child women in West China under the universal 2-child policy: A STROBE compliant prospective cohort study	Edinburgh Postnatal Depression Scale	≥10	yes	5
Bo et al. [110]	2020	China	Prevalence of depressive symptoms among Chinese pregnant and postpartum women during the COVID-19 pandemic	Patient Health Questionnaire	≥5	yes	2
Lu et al. [111]	2020	China	Mental health outcomes among Chinese prenatal and postpartum women after the implementation of universal two-child policy	Edinburgh Postnatal Depression Scale	≥13	yes	5
Tang et al. [112]	2019	China	Influencing factors for prenatal Stress, anxiety and depression in early pregnancy among women in Chongqing, China	Self Rating Depression Scale	≥0.5	yes	4
Sha et al. [113]	2019	China	A prospective study of maternal postnatal depressive symptoms with	Edinburgh Postnatal Depression Scale	≥10	yes	5

© 2023 Mitchell AR et al. *JAMA Psychiatry*.

			infant-feeding practices in a Chinese birth cohort				
Mak et al. [114]	2019	China	Physical activity during early pregnancy and antenatal depression: A prospective cohort study	Edinburgh Postnatal Depression Scale	≥13	yes	5
Ma et al. [115]	2019	China	The impact of resilience on prenatal anxiety and depression among pregnant women in Shanghai	Centre of Epidemiological Studies-Depression	≥16	yes	5
Gao et al. [116]	2019	China	Association of sleep quality during pregnancy with stress and depression: a prospective birth cohort study in China	Edinburgh Postnatal Depression Scale	≥9	yes	4
Hu et al. [117]	2019	China	Association between social and family support and antenatal depression: a hospital-based study in Chengdu, China	Edinburgh Postnatal Depression Scale	≥10	yes	6
Duan et al. [118]	2019	China	Relationship between trait neuroticism and suicidal ideation among postpartum women in China: Testing a mediation model	Edinburgh Postnatal Depression Scale	≥12	yes	5
Ding et al. [119]	2019	China	"Doing the month" and postpartum depression among Chinese women: A Shanghai prospective cohort study	Edinburgh Postnatal Depression Scale	≥10	yes	6
Zhou et al. [120]	2018	China	Associations between social capital and maternal depression: results from a follow-up study in China	Edinburgh Postnatal Depression Scale	≥9	yes	5
Zhang et al. [121]	2018	China	Prevalence and relevant factors of anxiety and depression among pregnant women in a cohort study from south-east China	Self Rating Depression Scale	≥53	yes	4
Zheng et al. [122]	2018	China	Changes in maternal self-efficacy, postnatal depression symptoms and social support among Chinese primiparous women during the initial postpartum period: A longitudinal study	Edinburgh Postnatal Depression Scale	≥13	yes	5

© 2023 Mitchell AR et al. *JAMA Psychiatry.*

Yu et al. [123]	2018	China	Association of intimate partner violence during pregnancy, prenatal depression, and adverse birth outcomes in Wuhan, China	Centre of Epidemiological Studies-Depression	≥20	yes	4
Xiong et al. [124]	2018	China	Prevalence and factors associated with postpartum depression in women from single-child families	Edinburgh Postnatal Depression Scale	≥10	yes	5
Song et al. [125]	2018	China	ABO blood types and postpartum depression among Chinese women: A prospective cohort study in Tianjin, China	Edinburgh Postnatal Depression Scale	not specified	yes	4
Shi et al. [126]	2018	China	Maternal depression and suicide at immediate prenatal and early postpartum periods and psychosocial risk factors	Edinburgh Postnatal Depression Scale	≥12	yes	3
Chen et al. [127]	2018	China	Incidence of and social-demographic and obstetric factors associated with postpartum depression: differences among ethnic Han and Kazak women of Northwestern China	Edinburgh Postnatal Depression Scale	≥13	yes	6
Zhou et al. [128]	2017	China	Social capital and antenatal depression among Chinese primiparas: A cross-sectional survey	Edinburgh Postnatal Depression Scale	≥9	yes	6
Zeng et al. [129]	2017	China	Retinoids, anxiety and peripartum depressive symptoms among Chinese women: a prospective cohort study	Edinburgh Postnatal Depression Scale	≥10	yes	3
Wang et al. [130]	2017	China	Living with parents or with parents-in-law and postpartum depression: A preliminary investigation in China	Edinburgh Postnatal Depression Scale	≥13	yes	6
Yu et al. [131]	2017	China	Sleep was associated with depression and anxiety status during pregnancy: a prospective longitudinal study	Self Rating Depression Scale	≥53	yes	4

© 2023 Mitchell AR et al. *JAMA Psychiatry*.

Liu et al. [132]	2017	China	Risk factors for postpartum depression among Chinese women: path model analysis	Edinburgh Postnatal Depression Scale	>10	yes	2
Li et al. [133]	2017	China	Maternal history of child maltreatment and maternal depression risk in the perinatal period: A longitudinal study	Edinburgh Postnatal Depression Scale	≥12	yes	3
Li et al. [134]	2017	China	Social support and depression across the perinatal period: A longitudinal study	Edinburgh Postnatal Depression Scale	≥12	yes	4
Huang et al. [135]	2017	China	The association between second-hand smoke exposure and depressive symptoms among pregnant women	Centre of Epidemiological Studies-Depression	≥16	yes	5
Wang et al. [136]	2016	China	Negative Life Events and Antenatal Depression among Pregnant Women in Rural China: The Role of Negative Automatic Thoughts	Edinburgh Postnatal Depression Scale	≥13	yes	5
Li et al. [137]	2016	China	Path model of antenatal stress and depressive symptoms among Chinese primipara in late pregnancy	Self Rating Depression Scale	≥50	yes	5
Zeng et al. [138]	2015	China	Prevalence and predictors of antenatal depressive symptoms among Chinese women in their third trimester: a cross-sectional survey	Self Rating Depression Scale	≥60	yes	6
Ren et al. [139]	2015	China	Depression, Social Support, and Coping Styles among Pregnant Women after the Lushan Earthquake in Ya'an, China	Edinburgh Postnatal Depression Scale	≥14	yes	6
Fu et al. [140]	2015	China	Association between serum 25-hydroxyvitamin D levels measured 24 hours after delivery and postpartum depression	Edinburgh Postnatal Depression Scale	≥12	yes	4
Deng et al. [141]	2014	China	Prevalence and risk factors of postpartum depression in a population-based sample of women in Tangxia Community, Guangzhou	Edinburgh Postnatal Depression Scale	≥13	yes	5

© 2023 Mitchell AR et al. *JAMA Psychiatry*.

Dong et al. [142]	2013	China	Depression and its risk factors among pregnant women in 2008 Sichuan earthquake area and non-earthquake struck area in China	Edinburgh Postnatal Depression Scale	≥10	yes	6
Lau [143]	2013	China	Risk factors associated with antenatal depressive symptomatology among Chengdu Chinese women	Edinburgh Postnatal Depression Scale	>9	yes	6
Zhang et al. [144]	2012	China	Relationship between domestic violence and postnatal depression among pregnant Chinese women	Edinburgh Postnatal Depression Scale	≥13	yes	5
Qu et al. [145]	2012	China	Posttraumatic stress disorder and depression among new mothers at 8 months later of the 2008 Sichuan earthquake in China	Centre of Epidemiological Studies-Depression	≥16	yes	4
Qu et al. [146]	2012	China	The impact of the catastrophic earthquake in China's Sichuan province on the mental health of pregnant women	Edinburgh Postnatal Depression Scale	≥10	yes	5
Li et al. [147]	2012	China	Health-related quality of life among pregnant women with and without depression in Hubei, China	Edinburgh Postnatal Depression Scale	≥10	yes	6
Mao et al. [148]	2011	China	A comparison of postnatal depression and related factors between Chinese new mothers and fathers	Edinburgh Postnatal Depression Scale	≥13	yes	5
Lau et al. [149]	2011	China	Severe antenatal depressive symptoms before and after the 2008 Wenchuan earthquake in Chengdu, China	Edinburgh Postnatal Depression Scale	≥15	yes	4
Xie et al. [150]	2010	China	Prenatal family support, postnatal family support and postpartum depression	Edinburgh Postnatal Depression Scale	≥13	yes	4
Lau et al. [151]	2010	China	Validation of the Mainland Chinese version of the Edinburgh Postnatal Depression Scale in Chengdu mothers	Edinburgh Postnatal Depression Scale	≥10	yes	5

© 2023 Mitchell AR et al. *JAMA Psychiatry*.

Xie et al. [152]	2009	China	Fetal sex, social support, and postpartum depression	Edinburgh Postnatal Depression Scale	≥13	yes	4
Wan et al. [153]	2009	China	Postpartum depression and traditional postpartum care in China: role of zuoyuezi	Edinburgh Postnatal Depression Scale	≥13	yes	6
Qiao et al. [154]	2009	China	The prevalence and related risk factors of anxiety and depression symptoms among Chinese pregnant women in Shanghai	Hospital Anxiety and Depression Scale	≥9	no	5
Gao et al. [155]	2009	China	Depression, perceived stress, and social support among first-time Chinese mothers and fathers in the postpartum period	Edinburgh Postnatal Depression Scale	≥13	yes	6
Xie et al. [156]	2007	China	Fetal gender and postpartum depression in a cohort of Chinese women	Edinburgh Postnatal Depression Scale	≥13	yes	5
Wang et al. [157]	2003	China	A comparative study of postnatal depression and its predictors in Taiwan and mainland China	Beck Depression Inventory	≥13	yes	6
Democratic Republic of Congo							
Yotebieng et al. [158]	2017	Democratic Republic of Congo	Depression, retention in care, and uptake of PMTCT service in Kinshasa, the Democratic Republic of Congo: a prospective cohort	Patient Health Questionnaire	≥15	no	5
Egypt							
Goweda et al. [159]	2020	Egypt	Prevalence and associated risk factors of postpartum depression: A cross sectional study	Edinburgh Postnatal Depression Scale	≥12	no	6
Wassif et al. [160]	2019	Egypt	Assessment of Postpartum Depression and Anxiety among Females Attending Primary Health Care Facilities in Qaliubeya Governorate, Egypt	Depression Anxiety Stress Scale	≥10	yes	5
Abdelhai et al. [161]	2015	Egypt	Screening for antepartum anxiety and depression and their association with domestic violence among Egyptian pregnant women	Hospital Anxiety and Depression Scale	>10	yes	5

© 2023 Mitchell AR et al. *JAMA Psychiatry*.

Mohamed et al. [162]	2014	Egypt	A social and Biological Approach for Postpartum Depression in Egypt	Edinburgh Postnatal Depression Scale	≥10	no	6
Eritrea							
Gebregziabher et al. [163]	2020	Eritrea	Prevalence and associated factors of postpartum depression among postpartum mothers in central region, Eritrea: a health facility based survey	Structured Clinical Interview DSM-IV criteria	n/a	--	7
Eswatini							
Dlamini et al. [164]	2019	Eswatini	Prevalence and factors associated with postpartum depression at a primary healthcare facility in Eswatini	Edinburgh Postnatal Depression Scale	≥13	no	6
Malqvist et al. [165]	2016	Eswatini	Screening for Antepartum Depression Through Community Health Outreach in Swaziland	Edinburgh Postnatal Depression Scale	≥13	no	5
Ethiopia							
Zewdu et al. [166]	2021	Ethiopia	Prevalence of suicidal ideation and associated factors among HIV positive perinatal women on follow-up at Gondar town health institutions, Northwest Ethiopia: a cross-sectional study	Edinburgh Postnatal Depression Scale	postnatal women ≥13<br>antenatal women ≥7	yes	5
Zelalem et al. [167]	2020	Ethiopia	Antenatal depression and its correlates on northwestern Ethiopian women: community-based cross-sectional study	Edinburgh Postnatal Depression Scale	≥13	yes	5
Tesfaye et al. [168]	2021	Ethiopia	Antenatal Depression and Associated Factors among Pregnant Women Attending Antenatal Care Service in Kochi Health Center, Jimma Town, Ethiopia	Patient Health Questionnaire	≥10	yes	5
Dadi et al. [169]	2021	Ethiopia	Effect of perinatal depression on risk of adverse infant health outcomes in mother-infant dyads in Gondar town: a causal analysis	Edinburgh Postnatal Depression Scale	≥12	yes	5
Bante et al. [170]	2021	Ethiopia	Comorbid anxiety and depression: Prevalence and associated factors	Patient Health Questionnaire	≥5	yes	6

© 2023 Mitchell AR et al. *JAMA Psychiatry*.

			among pregnant women in Arba Minch zuria district, Gamo zone, southern Ethiopia				
Ashenafi et al. [171]	2021	Ethiopia	The role of intimate partner violence victimization during pregnancy on maternal postpartum depression in Eastern Ethiopia	Edinburgh Postnatal Depression Scale	≥13	yes	6
Wubetu et al. [172]	2020	Ethiopia	Prevalence of postpartum depression and associated factors among postnatal care attendees in Debre Berhan, Ethiopia, 2018	Edinburgh Postnatal Depression Scale	≥13	unclear	5
Tiki et al. [173]	2020	Ethiopia	Prevalence and factors associated with depression among pregnant mothers in the West Shoa zone, Ethiopia: A community-based cross-sectional study	Patient Health Questionnaire	≥8	yes	6
Necho et al. [174]	2020	Ethiopia	The association of intimate partner violence with postpartum depression in women during their first month period of giving delivery in health centers at Dessie town, 2019	Edinburgh Postnatal Depression Scale	≥13	yes	5
Dadi et al. [175]	2020	Ethiopia	Antenatal depression and its potential causal mechanisms among pregnant mothers in Gondar town: application of structural equation model	Edinburgh Postnatal Depression Scale	≥12	yes	4
Chuma et al. [176]	2020	Ethiopia	Magnitude and predictors of antenatal depression among pregnant women attending antenatal care in Sodo town, Southern Ethiopia: Facility-based cross-sectional study	Edinburgh Postnatal Depression Scale	≥13	yes	6
Ayen et al. [177]	2020	Ethiopia	Antepartum Depression and Associated Factors Among Pregnant Women Attending ANC Clinics in Gurage Zone Public	Edinburgh Postnatal Depression Scale	≥13	yes	5

© 2023 Mitchell AR et al. *JAMA Psychiatry*.

			Health Institutions, SNNPR, Ethiopia, 2019				
Anato et al. [178]	2020	Ethiopia	Maternal depression is associated with child undernutrition: A cross-sectional study in Ethiopia	Edinburgh Postnatal Depression Scale	≥13	yes	5
Lodebo et al. [179]	2020	Ethiopia	Magnitude of antenatal depression and associated factors among pregnant women in West Badewacho Woreda, Hadiyya Zone, South Ethiopia: Community based cross sectional study	Edinburgh Postnatal Depression Scale	≥13	yes	6
Dadi et al. [180]	2020	Ethiopia	Causal mechanisms of postnatal depression among women in Gondar town, Ethiopia: application of a stress-process model with generalized structural equation modelling	Edinburgh Postnatal Depression Scale	≥6	yes	6
Shitu et al. [181]	2019	Ethiopia	Postpartum depression and associated factors among mothers who gave birth in the last twelve months in Ankesha district, Awi zone, North West Ethiopia	Edinburgh Postnatal Depression Scale	≥8	yes	6
Habtamu Belete et al. [182]	2019	Ethiopia	Prevalence of antenatal depression and associated factors among pregnant women in Aneded woreda, North West Ethiopia: a community based cross-sectional study	Beck Depression Inventory II	≥16	yes	4
Bitew et al. [183]	2019	Ethiopia	Antenatal predictors of incident and persistent postnatal depressive symptoms in rural Ethiopia: a population-based prospective study	Patient Health Questionnaire	≥5	yes	6
Belay et al. [184]	2019	Ethiopia	Intimate partner violence and maternal depression during pregnancy: A community-based cross-sectional study in Ethiopia	Edinburgh Postnatal Depression Scale	≥13	yes	6
Abebe et al. [185]	2019	Ethiopia	Postpartum depression and associated factors among mothers	Edinburgh Postnatal Depression Scale	≥13	yes	6

© 2023 Mitchell AR et al. *JAMA Psychiatry*.

			in Bahir Dar Town, Northwest Ethiopia				
Abadiga [186]	2019	Ethiopia	Magnitude and associated factors of postpartum depression among women in Nekemte town, East Wollega zone, west Ethiopia, 2019: A community-based study	Edinburgh Postnatal Depression Scale	≥10	unclear	6
Duko et al. [187]	2019	Ethiopia	Depression among pregnant women and associated factors in Hawassa city, Ethiopia: an institution-based cross-sectional study	Edinburgh Postnatal Depression Scale	≥13	yes	5
Woldetensay et al. [188]	2018	Ethiopia	The role of nutrition, intimate partner violence and social support in prenatal depressive symptoms in rural Ethiopia: community based birth cohort study	Patient Health Questionnaire	≥8	yes	6
Toru et al. [189]	2018	Ethiopia	Magnitude of postpartum depression and associated factors among women in Mizan Aman town, Bench Maji zone, Southwest Ethiopia 11 Medical and Health Sciences 1117 Public Health and Health Services	Edinburgh Postnatal Depression Scale	≥10	yes	4
Kerie et al. [190]	2018	Ethiopia	Prevalence and associated factors of postpartum depression in Southwest, Ethiopia, 2017: a cross-sectional study	Edinburgh Postnatal Depression Scale	≥10	unclear	5
Azale et al. [191]	2018	Ethiopia	Coping strategies of women with postpartum depression symptoms in rural Ethiopia: a cross-sectional community study	Patient Health Questionnaire	≥5	unclear	6
Adamu et al. [192]	2018	Ethiopia	Domestic violence as a risk factor for postpartum depression among Ethiopian women: Facility based study	Edinburgh Postnatal Depression Scale	≥13	yes	3
Fantahun et al. [193]	2018	Ethiopia	Prevalence and factors associated with postpartum depression among mothers attending public health	Edinburgh Postnatal Depression Scale	≥13	yes	6

© 2023 Mitchell AR et al. *JAMA Psychiatry*.

			centers of Addis Ababa, Ethiopia, 2016				
Belay et al. [194]	2018	Ethiopia	Prevalence of antenatal depression and associated factors among pregnant women attending antenatal care at Dubti Hospital: A case of pastoralist region in Northeast Ethiopia	Beck Depression Inventory II	≥17	yes	6
Mossie et al. [195]	2017	Ethiopia	Prevalence of Antenatal Depressive Symptoms and Associated Factors among Pregnant Women in Maichew, North Ethiopia: An Institution Based Study	Beck Depression Inventory	≥14	no	5
Bitew et al. [196]	2017	Ethiopia	Antenatal depressive symptoms and utilisation of delivery and postnatal care: a prospective study in rural Ethiopia	Patient Health Questionnaire	≥5	yes	6
Bisetegn et al. [197]	2016	Ethiopia	Prevalence and Predictors of Depression among Pregnant Women in Debretabor Town, Northwest Ethiopia	Edinburgh Postnatal Depression Scale	≥12	yes	5
Ayele et al. [198]	2016	Ethiopia	Prevalence and Associated Factors of Antenatal Depression among Women Attending Antenatal Care Service at Gondar University Hospital, Northwest Ethiopia	Beck Depression Inventory	≥16	unclear	4
Biratu et al. [199]	2015	Ethiopia	Prevalence of antenatal depression and associated factors among pregnant women in Addis Ababa, Ethiopia: a cross-sectional study	Edinburgh Postnatal Depression Scale	≥13	yes	6
Ghana							
Sefogah et al. [200]	2020	Ghana	Prevalence and key predictors of perinatal depression among postpartum women in Ghana	Patient Health Questionnaire	>5	no	4
Lillie et al. [201]	2020	Ghana	Prevalence and Correlates of Depression Among Pregnant Women Enrolled in a Maternal and Newborn Health Program in Rural	Patient Health Questionnaire	≥10	no	4

© 2023 Mitchell AR et al. *JAMA Psychiatry.*

			Northern Ghana: a Cross-sectional Survey				
Anokye et al. [202]	2018	Ghana	Prevalence of postpartum depression and interventions utilized for its management	Patient Health Questionnaire	>10	no	4
Weobong et al. [203]	2015	Ghana	Determinants of postnatal depression in rural Ghana: findings from the don population-based cohort study	Patient Health Questionnaire	≥2	yes	5
Weobong et al. [204]	2015	Ghana	Association between probable postnatal depression and increased infant mortality and morbidity: findings from the DON population-based cohort study in rural Ghana	Patient Health Questionnaire	≥2	yes	6
Weobong et al. [205]	2014	Ghana	Association of antenatal depression with adverse consequences for the mother and newborn in rural Ghana: findings from the DON population-based cohort study	Patient Health Questionnaire	≥2	yes	6
Weobong et al. [206]	2014	Ghana	Prevalence and determinants of antenatal depression among pregnant women in a predominantly rural population in Ghana: the DON population-based study	Patient Health Questionnaire	≥2	yes	6
India							
Raghavan et al. [207]	2021	India	Prevalence and risk factors of perinatal depression among women in rural Bihar: A community-based cross-sectional study	Edinburgh Postnatal Depression Scale	≥10	yes	3
Neelakanthi et al. [208]	2021	India	Prevalence and risk factors of depressive symptoms in the postpartum period: A cross-sectional study	Edinburgh Postnatal Depression Scale	≥10	unclear	5
Lanjewar et al. [209]	2021	India	Depressed Motherhood: prevalence and Covariates of Maternal Postpartum Depression among Urban Mothers in India	Edinburgh Postnatal Depression Scale	≥13	yes	4

© 2023 Mitchell AR et al. *JAMA Psychiatry*.

Basu et al. [210]	2021	India	Postpartum depression burden and associated factors in mothers of infants at an urban primary health center in Delhi, India	Edinburgh Postnatal Depression Scale	≥10	unclear	5
Ana et al. [211]	2021	India	Is physical activity in pregnancy associated with prenatal and postnatal depressive symptoms?: Results from MAASTHI cohort study in South India	Edinburgh Postnatal Depression Scale	>13	yes	5
Badiya et al. [212]	2020	India	Identification of clinical and psychosocial characteristics associated with perinatal depression in the south Indian population	Edinburgh Postnatal Depression Scale	antenatal women ≥13 postnatal women ≥10	yes	4
Murry et al. [213]	2020	India	Postpartum Depression and its Risk Factors among Indian Women	Edinburgh Postnatal Depression Scale	≥12	yes	6
Dahiya et al. [214]	2020	India	Prevalence and correlates of antenatal depression among women registered at antenatal clinic in North India	Edinburgh Postnatal Depression Scale	≥10	unclear	5
Amipara et al. [215]	2020	India	A Study on Postpartum Depression and its Association with Infant Feeding Practices and Infant Nutritional Status among Mothers Attending the Anganwadi Centers of Valsad District, Gujarat, India	Edinburgh Postnatal Depression Scale	>10.5	yes	6
Khatri et al. [216]	2020	India	Psychological status of pregnant women during COVID-19 pandemic: A cross-sectional study from Mumbai	Patient Health Questionnaire	≥5	no	5
Goyal et al. [217]	2020	India	Psychiatric Morbidity, Cultural Factors, and Health-Seeking Behaviour in Perinatal Women: A Cross-Sectional Study from a Tertiary Care Centre of North India	Mini International Neuropsychiatric Interview	n/a	--	5
Kantipudi et al. [218]	2020	India	Antenatal Depression and Generalized Anxiety Disorder in a Tertiary Hospital in South India	Patient Health Questionnaire	≥10	no	4

© 2023 Mitchell AR et al. *JAMA Psychiatry*.

Sheeba et al. [219]	2019	India	Prenatal Depression and Its Associated Risk Factors Among Pregnant Women in Bangalore: A Hospital Based Prevalence Study	Edinburgh Postnatal Depression Scale	≥13	unclear	4
Agarwala et al. [220]	2019	India	Prevalence and predictors of postpartum depression among mothers in the rural areas of Udupi Taluk, Karnataka, India: A cross-sectional study	Edinburgh Postnatal Depression Scale	≥10	yes	5
Joshi et al. [221]	2019	India	Maternal depression and its association with responsive feeding and nutritional status of infants: A cross-sectional study from a rural medical college in central India	Edinburgh Postnatal Depression Scale	≥10	yes	5
Jaya Salengia et al. [222]	2019	India	The Relationship between Maternal Confidence, Infant Temperament, and Postpartum Depression	Edinburgh Postnatal Depression Scale	≥10	yes	6
Fuhr et al. [223]	2019	India	Delivering the Thinking Healthy Programme for perinatal depression through peers: an individually randomised controlled trial in India	Patient Health Questionnaire	≥10	unclear	6
Kale et al. [224]	2019	India	Postpartum depression prevalence in a tertiary care hospital in Mumbai, Maharashtra, India	Edinburgh Postnatal Depression Scale	>13	yes	5
Jha et al. [225]	2018	India	Fear of childbirth and depressive symptoms among postnatal women: A cross-sectional survey from Chhattisgarh, India	Edinburgh Postnatal Depression Scale	≥10	yes	5
Rathod et al. [226]	2018	India	Characteristics of perinatal depression in rural central, India: A cross-sectional study 11 Medical and Health Sciences 1117 Public Health and Health Services	Edinburgh Postnatal Depression Scale	≥10	yes	4
Gonzalez-Mesa et al. [227]	2018	India	Cultural factors influencing antenatal depression: A cross-sectional study in a cohort of Turkish and Spanish women at the beginning of the pregnancy	Edinburgh Postnatal Depression Scale	≥11	yes	4

© 2023 Mitchell AR et al. *JAMA Psychiatry*.

Kishore et al. [228]	2018	India	Life events and depressive symptoms among pregnant women in India: Moderating role of resilience and social support	Edinburgh Postnatal Depression Scale	≥11	yes	5
Zaidi et al. [229]	2017	India	Postpartum Depression in Women: A Risk Factor Analysis	Edinburgh Postnatal Depression Scale	≥10	yes	4
Shidhaye et al. [230]	2017	India	Association of gender disadvantage factors and gender preference with antenatal depression in women: a cross-sectional study from rural Maharashtra	Edinburgh Postnatal Depression Scale	>12	no	6
Goyal et al. [231]	2017	India	Correlation of Infant Gender with Postpartum Maternal and Paternal Depression and Exclusive Breastfeeding Rates	Edinburgh Postnatal Depression Scale	≥11	yes	4
Boggaram et al. [232]	2017	India	An exploratory study of identification of psychiatric disorders during pregnancy	Mini International Neuropsychiatric Interview	n/a	--	6
George et al. [233]	2016	India	Antenatal depression in coastal South India: Prevalence and risk factors in the community	Edinburgh Postnatal Depression Scale	≥10	yes	5
Sheela et al. [234]	2016	India	Screening for Postnatal Depression in a Tertiary Care Hospital	Edinburgh Postnatal Depression Scale	≥13	yes	5
LakshmiBhuvana et al. [235]	2016	India	Prevalence of postpartum depression at an Indian tertiary care teaching hospital	Hamilton Depression Rating Scale	not specified	no	4
Srinivasan et al. [236]	2015	India	Assessment of burden of depression during pregnancy among pregnant women residing in rural setting of Chennai	Edinburgh Postnatal Depression Scale	≥13	unclear	5
Shivalli et al. [237]	2015	India	Postnatal depression among rural women in South India: do socio-demographic, obstetric and pregnancy outcome have a role to play?	Edinburgh Postnatal Depression Scale	≥13	yes	6
Patel et al. [238]	2015	India	Characteristics of Postpartum Depression in Anand District, Gujarat, India	Edinburgh Postnatal Depression Scale	≥11	yes	5

© 2023 Mitchell AR et al. *JAMA Psychiatry*.

Johnson et al. [239]	2015	India	Postnatal depression among women availing maternal health services in a rural hospital in South India	Edinburgh Postnatal Depression Scale	≥13	yes	5
Bodhare et al. [240]	2015	India	Postnatal quality of life, depressive symptoms, and social support among women in southern India	Edinburgh Postnatal Depression Scale	≥5	yes	5
Gupta et al. [241]	2013	India	Postpartum depression in North Indian women: Prevalence and risk factors	Primary Care Evaluation of Mental Disorders	n/a	--	5
Dubey et al. [242]	2012	India	Prevalence and associated risk factors for postpartum depression in women attending a tertiary hospital, Delhi, India	Edinburgh Postnatal Depression Scale	≥10	yes	5
Ghosh et al. [243]	2011	India	Evaluation of post partum depression in a tertiary hospital	Edinburgh Postnatal Depression Scale	≥13	no	4
Savarimuthu et al. [244]	2010	India	Post-partum depression in the community: a qualitative study from rural South India	Structured Clinical Interview DSM-IV criteria	n/a	--	5
Mariam et al. [245]	2009	India	Antenatal psychological distress and postnatal depression: A prospective study from an urban clinic	Edinburgh Postnatal Depression Scale	≥12	yes	5
Nagpal et al. [246]	2008	India	An exploratory study to evaluate the utility of an adapted Mother Generated Index (MGI) in assessment of postpartum quality of life in India	Edinburgh Postnatal Depression Scale	≥13	unclear	4
Patel et al. [247]	2003	India	Postnatal depression and infant growth and development in low income countries: a cohort study from Goa, India	Edinburgh Postnatal Depression Scale	≥12	no	6
Patel et al. [248]	2002	India	Gender, poverty, and postnatal depression: a study of mothers in Goa, India	Edinburgh Postnatal Depression Scale	≥12	yes	5
Chandran et al. [249]	2002	India	Post-partum depression in a cohort of women from a rural area of Tamil Nadu, India. Incidence and risk factors	Interview with ICD-10 criteria	n/a	--	7

© 2023 Mitchell AR et al. *JAMA Psychiatry*.

Indonesia							
Misrawati et al. [250]	2020	Indonesia	Antenatal depression and its associated factors among pregnant women in Jakarta, Indonesia	Depression Symptom Recognition Sheet	≥5	yes	5
Rahmadhani et al. [251]	2020	Indonesia	Gender of baby and postpartum depression among adolescent mothers in central Java, Indonesia	Edinburgh Postnatal Depression Scale	≥13	yes	4
Nurbaeti et al. [252]	2019	Indonesia	Association between psychosocial factors and postpartum depression in South Jakarta, Indonesia	Edinburgh Postnatal Depression Scale	≥12	yes	5
Nurbaeti et al. [253]	2018	Indonesia	Postpartum Depression in Indonesian Mothers: Its Changes and Predicting Factors	Edinburgh Postnatal Depression Scale	≥13	yes	4
Idaiani et al. [254]	2018	Indonesia	The determinants of perinatal depression (PND) in Tebet Merdeka, Jakarta and Sindangbarang, Bogor Indonesia	Edinburgh Postnatal Depression Scale	≥13	unclear	6
Edwards et al. [255]	2006	Indonesia	Postnatal Depression in Surabaya, Indonesia	Edinburgh Postnatal Depression Scale	≥10	unclear	4
Iran							
Najafi-Sharjabad et al. [256]	2021	Iran	Evaluation of Sociocultural, Obstetric, and Child Related Factors Associated with Postpartum Depression in Bushehr, Southwest of Iran	Edinburgh Postnatal Depression Scale	≥10	yes	5
Moradi et al. [257]	2020	Iran	Factors Related to Postpartum Depression in Mothers Referred to Kermanshah Health Centers, Iran	Edinburgh Postnatal Depression Scale	≥12	unclear	6
Khoshgoo et al. [258]	2020	Iran	The relationship between household food insecurity and depressive symptoms among pregnant women: A cross sectional study	Beck Depression Inventory II	≥14	yes	4
Effati-Daryani et al. [259]	2020	Iran	Depression, stress, anxiety and their predictors in Iranian pregnant women during the outbreak of COVID-19	Depression Anxiety Stress Scale	≥5	yes	2
Afshari et al. [260]	2020	Iran	Prevalence and related factors of postpartum depression among	Edinburgh Postnatal Depression Scale	>12	yes	6

© 2023 Mitchell AR et al. *JAMA Psychiatry*.

			reproductive aged women in Ahvaz, Iran				
Vaezi et al. [261]	2019	Iran	The association between social support and postpartum depression in women: A cross sectional study	Edinburgh Postnatal Depression Scale	≥13	yes	5
Parsa et al. [262]	2019	Iran	Prediction of postpartum depression based on women's quality of life	Edinburgh Postnatal Depression Scale	≥10	yes	5
Matinnia et al. [263]	2018	Iran	Psychological Risk Factors for Postnatal Depression: A Prospective Study of Iranian Low Income Primigravidae at Health Care Centres	Edinburgh Postnatal Depression Scale	≥13	yes	4
Alipour et al. [264]	2018	Iran	Psychological profiles of risk for antenatal depression and anxiety in Iranian sociocultural context	General Health Questionnaire	>6	yes	3
Abdollahi et al. [265]	2017	Iran	Stability of depressive symptoms over 3 months post-partum	Edinburgh Postnatal Depression Scale	≥12	yes	4
Rouhi et al. [266]	2017	Iran	Male child preference: Is it a risk factor for antenatal depression among Iranian women?	Edinburgh Postnatal Depression Scale	≥13	yes	5
Mahmoodi et al. [267]	2017	Iran	Mother-father differences in postnatal psychological distress and its determinants in Iran	General Health Questionnaire	≥23	yes	5
Jamali et al. [268]	2017	Iran	The relationship between intimate partner violence and antenatal depression: a cross-sectional study in Iran	Beck Depression Inventory	≥16	unclear	4
Iranpour et al. [269]	2017	Iran	Association between Caffeine Consumption during Pregnancy and Postpartum Depression: A Population-Based Study	Edinburgh Postnatal Depression Scale	≥13	unclear	4
Moshki et al. [270]	2016	Iran	Relationships among depression during pregnancy, social support and health locus of control among Iranian pregnant women	Edinburgh Postnatal Depression Scale	≥12	yes	5
Abdollahi et al. [271]	2016	Iran	Prediction of incidence and bio-psycho-socio-cultural risk factors of post-partum depression	Edinburgh Postnatal Depression Scale	>12	yes	5

© 2023 Mitchell AR et al. *JAMA Psychiatry*.

			immediately after birth in an Iranian population				
Abdollahi et al. [272]	2016	Iran	Postpartum mental health in relation to sociocultural practices	Edinburgh Postnatal Depression Scale	>12	yes	6
Habibzadeh et al. [273]	2016	Iran	Evaluation of effective factors and its prevalence on postpartum depression among women in the city of Qom, Iran	Edinburgh Postnatal Depression Scale	>12	yes	5
Jarahi et al. [274]	2015	Iran	Evaluation of Depression and the Contributing Factors in Pregnant Women Referring to Urban and Rural Health Care Centers of Sarakhs City, Iran	Edinburgh Postnatal Depression Scale	≥11	yes	5
Sadat et al. [275]	2014	Iran	Effect of mode of delivery on postpartum depression in Iranian women	Edinburgh Postnatal Depression Scale	≥13	yes	4
Abdollahi et al. [276]	2014	Iran	Predictors and incidence of post-partum depression: a longitudinal cohort study	Edinburgh Postnatal Depression Scale	>12	yes	5
Abdollahi et al. [277]	2014	Iran	Postpartum depression and psycho-socio-demographic predictors	Edinburgh Postnatal Depression Scale	>12	yes	6
Rouhi et al. [278]	2012	Iran	Ethnicity as a risk factor for postpartum depression	Edinburgh Postnatal Depression Scale	≥13	yes	5
Alipour et al. [279]	2012	Iran	Anxiety and fear of childbirth as predictors of postnatal depression in nulliparous women	Edinburgh Postnatal Depression Scale	≥13	unclear	5
Rouhi et al. [280]	2011	Iran	Postpartum morbidity and help-seeking behaviours in Iran	Edinburgh Postnatal Depression Scale	≥13	yes	5
Abbaszadeh et al.[281]	2011	Iran	Violence during pregnancy and postpartum depression	Edinburgh Postnatal Depression Scale	≥13	yes	6
Kheirabadi et al. [282]	2010	Iran	Perinatal depression in a cohort study on Iranian women	Edinburgh Postnatal Depression Scale	>12	yes	4
Tashakori et al. [283]	2009	Iran	Assessment of some potential risk factors of postpartum depression	Edinburgh Postnatal Depression Scale	≥12	yes	4
Kheirabadi et al. [284]	2009	Iran	Risk factors of postpartum depression in rural areas of Isfahan Province, Iran	Beck Depression Inventory II	≥10	yes	5

© 2023 Mitchell AR et al. *JAMA Psychiatry*.

Montazeri et al. [285]	2007	Iran	The Edinburgh Postnatal Depression Scale (EPDS): translation and validation study of the Iranian version	Edinburgh Postnatal Depression Scale	≥10	yes	5
Iraq							
Al-Hashimi et al. [286]	2020	Iraq	Screening for depression during pregnancy using the Kurdish version of the Edinburgh Postnatal Depression Scale in Erbil city	Edinburgh Postnatal Depression Scale	≥13	yes	5
Ahmed et al. [287]	2012	Iraq	Screening for postpartum depression using Kurdish version of Edinburgh postnatal depression scale	Edinburgh Postnatal Depression Scale	≥10	no	6
Jamaica							
Bernard et al. [288]	2018	Jamaica	Antenatal depressive symptoms in Jamaica associated with limited perceived partner and other social support: A cross-sectional study	Edinburgh Postnatal Depression Scale	≥13	no	6
Wissart et al. [289]	2005	Jamaica	Prevalence of pre- and postpartum depression in Jamaican women	Self Rating Depression Scale	≥50	no	4
Jordan							
Mohammad et al. [290]	2018	Jordan	Postpartum depression symptoms among Syrian refugee women living in Jordan	Edinburgh Postnatal Depression Scale	>12	yes	6
Safadi et al. [291]	2016	Jordan	Demographic, maternal, and infant health correlates of post-partum depression in Jordan	Patient Health Questionnaire	unclear	no	4
Abujilban et al. [292]	2014	Jordan	Predictors of antenatal depression among Jordanian pregnant women in their third trimester	Edinburgh Postnatal Depression Scale	>13	yes	6
Abuidhail et al. 293	2014	Jordan	Characteristics of Jordanian depressed pregnant women: a comparison study	Edinburgh Postnatal Depression Scale	≥13	yes	6
Mohammad et al. [294]	2011	Jordan	Prevalence and factors associated with the development of antenatal and postnatal depression among Jordanian women	Edinburgh Postnatal Depression Scale	≥13	unclear	5

© 2023 Mitchell AR et al. *JAMA Psychiatry*.

Kenya							
Tuthill et al. [295]	2021	Kenya	Persistent Food Insecurity, but not HIV, is Associated with Depressive Symptoms Among Perinatal Women in Kenya: A Longitudinal Perspective	Centre of Epidemiological Studies-Depression	>16	no	5
Samia et al. [296]	2020	Kenya	Adverse Childhood Experiences and Changing Levels of Psychosocial Distress Scores across Pregnancy in Kenyan Women	Edinburgh Postnatal Depression Scale	>13	yes	5
Kimbui et al. [297]	2018	Kenya	A cross-sectional study of depression with comorbid substance use dependency in pregnant adolescents from an informal settlement of Nairobi: drawing implications for treatment and prevention work	Edinburgh Postnatal Depression Scale	≥8	yes	4
Ongeri et al. [298]	2018	Kenya	Demographic, psychosocial and clinical factors associated with postpartum depression in Kenyan women	Edinburgh Postnatal Depression Scale	antenatal women ≥13<br>postnatal women ≥10	yes	6
Madeghe et al. [299]	2016	Kenya	Postpartum depression and infant feeding practices in a low income urban settlement in Nairobi-Kenya	Edinburgh Postnatal Depression Scale	≥13	yes	4
Turan et al. [300]	2015	Kenya	Linkage to HIV care, postpartum depression, and HIV-related stigma in newly diagnosed pregnant women living with HIV in Kenya: A longitudinal observational study	Edinburgh Postnatal Depression Scale	≥13	yes	4
Kosovo							
Zejnullahu et al. [301]	2021	Kosovo	Prevalence of postpartum depression at the clinic for obstetrics and gynecology in Kosovo teaching hospital: Demographic, obstetric and psychosocial risk factors	Edinburgh Postnatal Depression Scale	≥12	no	4

© 2023 Mitchell AR et al. *JAMA Psychiatry*.

Lao							
Inthaphatha et al. [302]	2020	Lao	Factors associated with postpartum depression among women in Vientiane Capital, Lao People's Democratic Republic: A cross-sectional study	Edinburgh Postnatal Depression Scale	≥10	yes	6
Lebanon							
Badr et al. [303]	2018	Lebanon	Is the Effect of Postpartum Depression on Mother-Infant Bonding Universal?	Edinburgh Postnatal Depression Scale	≥13	no	3
El-Hachem et al. [304]	2014	Lebanon	Early identification of women at risk of postpartum depression using the Edinburgh Postnatal Depression Scale (EPDS) in a sample of Lebanese women	Edinburgh Postnatal Depression Scale	≥9	no	4
Chaaya et al. [305]	2002	Lebanon	Postpartum depression: prevalence and determinants in Lebanon	Edinburgh Postnatal Depression Scale	≥13	no	4
Macedonia							
Pop-Jordanova et al. [306]	2013	Macedonia	The need for regular screening of postpartum depression	Beck Depression Inventory	≥10	no	4
Malawi							
Le Masters et al. [307]	2020	Malawi	"Pain in my heart": Understanding perinatal depression among women living with HIV in Malawi	Edinburgh Postnatal Depression Scale	≥10	yes	4
Chorwe-Sungani et al. [308]	2018	Malawi	A cross-sectional study of depression among women attending antenatal clinics in Blantyre district, Malawi	Edinburgh Postnatal Depression Scale	≥10	unclear	6
Dow et al. [309]	2014	Malawi	Postpartum depression and HIV infection among women in Malawi	Edinburgh Postnatal Depression Scale	>12	yes	5
Stewart et al. [310]	2014	Malawi	A cross-sectional study of antenatal depression and associated factors in Malawi	Self Reporting Questionnaire	≥5	yes	6
Malaysia							
Elias et al. [311]	2020	Malaysia	Antenatal depression; its prevalence of positive screen and the associating risk factors including labor and neonatal outcome	Edinburgh Postnatal Depression Scale	≥12	yes	5

© 2023 Mitchell AR et al. *JAMA Psychiatry*.

Nasreen et al. [312]	2018	Malaysia	Prevalence and determinants of antepartum depressive and anxiety symptoms in expectant mothers and fathers: results from a perinatal psychiatric morbidity cohort study in the east and west coasts of Malaysia	Edinburgh Postnatal Depression Scale	≥12	yes	6
Ahmad et al. [313]	2018	Malaysia	Postnatal depression and intimate partner violence: a nationwide clinic-based cross-sectional study in Malaysia	Edinburgh Postnatal Depression Scale	≥12 or positive for Q10	yes	6
Rashid et al. [314]	2017	Malaysia	Poor social support as a risk factor for antenatal depressive symptoms among women attending public antennal clinics in Penang, Malaysia	Edinburgh Postnatal Depression Scale	≥12	yes	5
Mohamad Yusuff et al. [315]	2016	Malaysia	Prevalence of antenatal depressive symptoms among women in Sabah, Malaysia	Edinburgh Postnatal Depression Scale	≥12	yes	6
Mohamad Yusuff et al. [316]	2015	Malaysia	Prevalence and risk factors for postnatal depression in Sabah, Malaysia: a cohort study	Edinburgh Postnatal Depression Scale	≥12	yes	5
Fadzil et al. [317]	2013	Malaysia	Risk factors for depression and anxiety among pregnant women in Hospital Tuanku Bainun, Ipoh, Malaysia	Mini International Neuropsychiatric Interview	n/a	--	5
Zainal et al. [318]	2012	Malaysia	Prevalence of postpartum depression in a hospital setting among Malaysian mothers	Mini International Neuropsychiatric Interview	n/a	--	6
Kadir et al. [319]	2009	Malaysia	Relationship between obstetric risk factors and postnatal depression in Malaysian women	Edinburgh Postnatal Depression Scale	≥12	yes	3
Azidah et al. [320]	2006	Malaysia	Postnatal depression and socio-cultural practices among postnatal mothers in Kota Bahru, Kelantan, Malaysia	Edinburgh Postnatal Depression Scale	>11	yes	6

© 2023 Mitchell AR et al. *JAMA Psychiatry*.

Kadir et al. [321]	2005	Malaysia	Postnatal depression in mothers attending primary care clinics in Kelantan, Malaysia	Edinburgh Postnatal Depression Scale	≥12	yes	5
Mahmud et al. [322]	2003	Malaysia	Revalidation of the Malay version of the Edinburgh postnatal depression scale (EPDS) among Malay postnatal women attending the Bakar Bata Health Center in Alor Setar, Kedah, North West of Peninsular Malaysia	Composite International Diagnostic Interview	n/a	--	6
Grace et al. [323]	2001	Malaysia	The relationship between post-natal depression, somatization and behaviour in Malaysian women	Edinburgh Postnatal Depression Scale	>12	no	6
Kit et al. [324]	1997	Malaysia	Incidence of postnatal depression in Malaysian women	Edinburgh Postnatal Depression Scale	≥13	no	6
Maldives							
Abdul Raheem et al. [325]	2019	Maldives	Maternal Depression and Breastfeeding Practices in the Maldives	Edinburgh Postnatal Depression Scale	>12	no	6
Abdul Raheem et al. [326]	2018	Maldives	Factors Associated with Maternal Depression in the Maldives: A Prospective Cohort Study	Edinburgh Postnatal Depression Scale	≥13	no	6
Mexico							
Marcos-Najera et al. [327]	2021	Mexico	A Cross-Cultural Analysis of the Prevalence and Risk Factors for Prenatal Depression in Spain and Mexico	Patient Health Questionnaire	≥10	yes	4
McRae et al. [328]	2020	Mexico	Blood manganese levels during pregnancy and postpartum depression: A cohort study among women in Mexico	Edinburgh Postnatal Depression Scale	≥13	yes	5
Alvarado-Esquivel et al. [329]	2016	Mexico	Unhappiness with the fetal gender is associated with depression in adult pregnant women attending prenatal care in a public hospital in Durango, Mexico	Edinburgh Postnatal Depression Scale	≥9	yes	6
Lara et al. [330]	2016	Mexico	Prenatal predictors of postpartum depression and postpartum	Patient Health Questionnaire	≥10	unclear	5

© 2023 Mitchell AR et al. *JAMA Psychiatry*.

			depressive symptoms in Mexican mothers: a longitudinal study				
de Castro et al. [331]	2015	Mexico	Risk profiles associated with postnatal depressive symptoms among women in a public sector hospital in Mexico: the role of sociodemographic and psychosocial factors	Edinburgh Postnatal Depression Scale	≥12	yes	4
Lara et al. [332]	2015	Mexico	Prevalence and incidence of perinatal depression and depressive symptoms among Mexican women	Structured Clinical Interview DSM-IV criteria	n/a	--	6
Lara et al. [333]	2014	Mexico	Intimate partner violence and depressive symptoms in pregnant Mexican women: national survey results	Centre of Epidemiological Studies-Depression	≥16	yes	3
deCastro et al. [334]	2011	Mexico	Risk and protective factors associated with postnatal depression in Mexican adolescents	Edinburgh Postnatal Depression Scale	≥13	yes	6
Mongolia							
Pollock et al. [335]	2009	Mongolia	Depression in Mongolian women over the first 2 months after childbirth: prevalence and risk factors	Self Reporting Questionnaire	≥9	yes	4
Morocco							
Alami et al. [336]	2006	Morocco	Prevalence and psychosocial correlates of depressed mood during pregnancy and after childbirth in a Moroccan sample	Mini International Neuropsychiatric Interview	n/a	--	4
Agoub et al. [337]	2005	Morocco	Prevalence of postpartum depression in a Moroccan sample	Mini International Neuropsychiatric Interview	n/a	--	5
Nepal							
Singh et al. [338]	2021	Nepal	Determining factors for the prevalence of depressive symptoms among postpartum mothers in lowland region in southern Nepal	Edinburgh Postnatal Depression Scale	≥13	yes	5
Pradhananga et al. [339]	2020	Nepal	Prevalence of postpartum depression in a tertiary health care	Edinburgh Postnatal Depression Scale	≥12	yes	4

© 2023 Mitchell AR et al. *JAMA Psychiatry*.

Maharjan et al. [340]	2019	Nepal	Prevalence and factors associated with depressive symptoms among post-partum mothers in Dhanusha district of Nepal	Edinburgh Postnatal Depression Scale	≥13	yes	5
Khadka et al. [341]	2019	Nepal	Prevalence and determinants of poor sleep quality and depression among postpartum women: A community-based study in Ramechhap district, Nepal	Patient Health Questionnaire	≥3	no	6
Chalise et al. [342]	2019	Nepal	Postpartum Depression and its Associated Factors: A Community-based Study in Nepal	Edinburgh Postnatal Depression Scale	≥13	yes	4
Joshi et al. [343]	2019	Nepal	Understanding the antepartum depressive symptoms and its risk factors among the pregnant women visiting public health facilities of Nepal	Edinburgh Postnatal Depression Scale	≥10	yes	5
Bhusal et al. [344]	2018	Nepal	Identifying the factors associated with depressive symptoms among postpartum mothers in Kathmandu, Nepal	Edinburgh Postnatal Depression Scale	≥12	yes	5
Aryal et al. [345]	2018	Nepal	Anxiety and Depression among Pregnant Women and Mothers of Children Under one Year in Sindupalchowk District	John Hopkins Symptoms Checklist	≥24.5	yes	5
Aihara et al. [346]	2016	Nepal	Household water insecurity, depression and quality of life among postnatal women living in urban Nepal	Edinburgh Postnatal Depression Scale	≥13	yes	6
Kumwar et al. [347]	2015	Nepal	Screening for Postpartum Depression and Associated Factors among Women who Deliver at a University Hospital, Nepal	Edinburgh Postnatal Depression Scale	>13	yes	4
Giri et al. [348]	2015	Nepal	Prevalence and factors associated with depressive symptoms among post-partum mothers in Nepal	Edinburgh Postnatal Depression Scale	≥10	unclear	5
Budhathoki et al. [349]	2012	Nepal	Violence against women by their husband and postpartum depression	Edinburgh Postnatal Depression Scale	≥13	yes	5

© 2023 Mitchell AR et al. *JAMA Psychiatry.*

Shakya et al. [350]	2008	Nepal	Depression during pregnancy in a tertiary care center of eastern Nepal	Hamilton Depression Rating Scale	≥7	no	6
Ho-Yen et al. [351]	2007	Nepal	Factors associated with depressive symptoms among postnatal women in Nepal	Edinburgh Postnatal Depression Scale	≥13	yes	6
Ho-Yen et al. [352]	2006	Nepal	The prevalence of depressive symptoms in the postnatal period in Lalitpur district, Nepal	Edinburgh Postnatal Depression Scale	>12	yes	6
Regmi et al. [353]	2002	Nepal	A controlled study of postpartum depression among Nepalese women: validation of the Edinburgh Postpartum Depression Scale in Kathmandu	Edinburgh Postnatal Depression Scale	≥13	yes	4
Nicaragua							
Verbeek et al. [354]	2015	Nicaragua	Anxiety and depression during pregnancy in Central America: A cross-sectional study among pregnant women in the developing country Nicaragua	Edinburgh Postnatal Depression Scale	≥12	no	5
Nigeria							
Okunola et al. [355]	2021	Nigeria	Predictors of postpartum depression among an obstetric population in South-Western Nigeria	Edinburgh Postnatal Depression Scale	≥13	yes	4
Adeyemo et al. [356]	2020	Nigeria	Prevalence and predictors of postpartum depression among postnatal women in Lagos, Nigeria	Edinburgh Postnatal Depression Scale	≥13	unclear	5
Oladeji et al. [357]	2019	Nigeria	Exploring differences between adolescents and adults with perinatal depression data from the expanding care for perinatal women with depression trial in Nigeria	Edinburgh Postnatal Depression Scale	≥12	yes	5
Oladeji et al. [358]	2019	Nigeria	Exploring differences between adolescents and adults with depression-data from the expanding care for perinatal women with depression trial in Nigeria	Edinburgh Postnatal Depression Scale	≥12	yes	6
Odinka et al. [359]	2019	Nigeria	Socio-demographic correlates of postpartum psychological distress	Hospital Anxiety and Depression Scale	≥11	yes	5

© 2023 Mitchell AR et al. *JAMA Psychiatry*.

			among apparently healthy mothers in two tertiary hospitals in Enugu, South-East Nigeria				
Agbaje et al. [360]	2019	Nigeria	Depressive and anxiety symptoms and associated factors among postnatal women in Enugu-North Senatorial District, South-East Nigeria: a cross-sectional study	Edinburgh Postnatal Depression Scale	≥13	unclear	5
Tungchama et al. [361]	2017	Nigeria	Independent socio-demographic and clinical correlates associated with the perception of quality of life of women with postpartum depression in North-central, Nigeria	Postnatal Depression Scale	≥13	yes	5
Thompson et al. [362]	2016	Nigeria	Prevalence of antenatal depression and associated risk factors among pregnant women attending antenatal clinics in Abeokuta North Local Government Area, Nigeria	Edinburgh Postnatal Depression Scale	>11	unclear	5
Sulyman et al. [363]	2016	Nigeria	Postnatal depression and its associated factors among Northeastern Nigerian women	Edinburgh Postnatal Depression Scale	≥13	yes	4
Ebeigbe et al. [364]	2008	Nigeria	Incidence and associated risk factors of postpartum depression in a tertiary hospital in Nigeria	Edinburgh Postnatal Depression Scale	≥9	yes	6
Adewuya et al. [365]	2006	Nigeria	Validation of the Edinburgh Postnatal Depression Scale as a screening tool for depression in late pregnancy among Nigerian women	Mini International Neuropsychiatric Interview	n/a	--	6
Owoeye et al. [366]	2006	Nigeria	Risk factors of postpartum depression and EPDS scores in a group of Nigerian women	Edinburgh Postnatal Depression Scale	≥12	no	5
Adewuya [367]	2006	Nigeria	Early postpartum mood as a risk factor for postnatal depression in Nigerian women	Schedule for Affective Disorders and Schizophrenia	n/a	--	7
Abiodun et al. [368]	2006	Nigeria	Postnatal depression in primary care populations in Nigeria	Edinburgh Postnatal Depression Scale	≥9	yes	5

© 2023 Mitchell AR et al. *JAMA Psychiatry.*

Adewuya et al. [369]	2005	Nigeria	Prevalence of postnatal depression in Western Nigerian women: A controlled study	Edinburgh Postnatal Depression Scale	≥10	yes	6
Adewuya et al. [370]	2005	Nigeria	Sociodemographic and obstetric risk factors for postpartum depressive symptoms in Nigerian women	Edinburgh Postnatal Depression Scale	≥9	no	6
Uwakwe et al. [371]	2003	Nigeria	Affective (depressive) morbidity in puerperal Nigerian women: Validation of the Edinburgh postnatal depression scale	Interview with ICD-10 criteria	n/a	--	5
Aderibigbe et al. [372]	1993	Nigeria	Postnatal emotional disorders in Nigerian women. A study of antecedents and associations	General Health Questionnaire	≥8	yes	2
Aderibigbe et al. [373]	1992	Nigeria	The validity of the 28-item General Health Questionnaire in a Nigerian antenatal clinic	Interview with DSM-IIIR criteria	n/a	--	3
Pakistan							
Premji et al. [374]	2020	Pakistan	Comorbid anxiety and depression among pregnant Pakistani women: Higher rates, different vulnerability characteristics, and the role of perceived stress	Edinburgh Postnatal Depression Scale	≥9	yes	5
Yadav et al. [375]	2020	Pakistan	Postpartum Depression: Prevalence and Associated Risk Factors Among Women in Sindh, Pakistan	Edinburgh Postnatal Depression Scale	≥10	no	4
Shahid et al. [376]	2020	Pakistan	Evaluation of psychological impact, depression, and anxiety among pregnant women during the COVID-19 pandemic in Lahore, Pakistan	Edinburgh Postnatal Depression Scale	≥10	unclear	5
Ishtiaque et al. [377]	2020	Pakistans	Prevalence of antenatal depression and associated risk factors among pregnant women attending antenatal clinics in Karachi, Pakistan	Patient Health Questionnaire	not specified	no	4

© 2023 Mitchell AR et al. *JAMA Psychiatry*.

Khan et al. [378]	2020	Pakistan	Major Depressive Disorder: An Alarming Stigma Of Pregnant Women	Patient Health Questionnaire	≥10	unclear	5
Habiba et al. [379]	2020	Pakistan	Prevalence and risk factors associated with prenatal depression among pregnant women in Faisalabad, Pakistan	Edinburgh Postnatal Depression Scale	≥10	unclear	5
Shehroz et al. [380]	2019	Pakistan	Depression and anxiety during pregnancy period	Interview with ICD-10 criteria	n/a	--	2
Sikander et al. [381]	2019	Pakistan	Delivering the Thinking Healthy Programme for perinatal depression through volunteer peers: a cluster randomised controlled trial in Pakistan	Patient Health Questionnaire	≥10	yes	6
Shagufta et al. [382]	2019	Pakistan	Prevalence, Differences, and Predictors of Anxiety and Depression among Pregnant and Non-Pregnant Women in Peshawar Khyber Pakhtunkhwa Pakistan	Hospital Anxiety and Depression Scale	not specified	no	1
Sabir et al. [383]	2019	Pakistan	Prevalence of antenatal depression among women receiving antenatal care during last trimester of pregnancy in a tertiary care private institute of Lahore	Golberg's Depression Scale	not specified	yes	5
Naseer et al. [384]	2019	Pakistan	Occurrence of psychiatric Disorders Among Pregnant Females	Present State Examination Schedule	n/a	--	5
Ayaz et al. [385]	2019	Pakistan	A descriptive analysis of factors of depression in pregnant women of Pakistan	Beck Depression Inventory	≥16	unclear	4
Anjum et al. [386]	2019	Pakistan	An analytical study of contributory factors of postpartum depression among women in Punjab, Pakistan	Edinburgh Postnatal Depression Scale	≥10	no	5
Maselko et al. [387]	2019	Pakistan	Father involvement in the first year of life: Associations with maternal mental health and child development outcomes in rural Pakistan	Patient Health Questionnaire	≥10	yes	4

© 2023 Mitchell AR et al. *JAMA Psychiatry*.

Gul et al. [388]	2019	Pakistan	Antenatal anxiety and depression among pregnant women attending tertiary care hospital, Mardan, Pakistan	Hamilton Depression Rating Scale	not specified	unclear	6
Zia et al. [389]	2018	Pakistan	Psychosocial factors causing depression during antenatal period and their association with progression of pregnancy	Beck Depression Inventory	>30	unclear	5
Sadiq et al. [390]	2018	Pakistan	Prospective study on prevalence and risk factors of post natal depression in Rawalpindi/Islamabad Pakistan	Edinburgh Postnatal Depression Scale	≥10	unclear	5
Ayyub et al. [391]	2018	Pakistan	Association Of Antenatal Depression And Household Food Insecurity Among Pregnant Women: A Cross sectional Study From Slums Of Lahore	Edinburgh Postnatal Depression Scale	≥12	no	5
Jamal et al. [392]	2018	Pakistan	Antenatal depression: Prevalence predictors and frequently employed coping strategies	Beck Depression Inventory	≥17	no	4
Shah et al. [393]	2017	Pakistan	Frequency of postpartum depression and its association with breastfeeding: A cross-sectional survey at immunization clinics in Islamabad, Pakistan	Edinburgh Postnatal Depression Scale	≥10	no	5
Saeed et al. [394]	2016	Pakistan	Effect of antenatal depression on maternal dietary intake and neonatal outcome: a prospective cohort	Edinburgh Postnatal Depression Scale	≥9	yes	4
Waqas et al. [395]	2015	Pakistan	Psychosocial factors of antenatal anxiety and depression in Pakistan: is social support a mediator?	Hospital Anxiety and Depression Scale	≥8	yes	4
Afridi et al. [396]	2014	Pakistan	Frequency of postnatal depression at a Tertiary care hospital	Edinburgh Postnatal Depression Scale	≥13	no	5
Husain et al. [397]	2014	Pakistan	Antenatal depression is not associated with low birth weight: A study from urban Pakistan	Edinburgh Postnatal Depression Scale	≥12	yes	4

© 2023 Mitchell AR et al. *JAMA Psychiatry*.

Safi et al. [398]	2013	Pakistan	Antenatal depression: Prevalence and risk factors for depression among pregnant women in Peshawar	Centre of Epidemiological Studies-Depression	≥15	no	5
Humayun et al. [399]	2013	Pakistan	Antenatal depression and its predictors in Lahore, Pakistan	Edinburgh Postnatal Depression Scale	≥10	no	6
Ali et al. [400]	2012	Pakistan	Frequency and associated factors for anxiety and depression in pregnant women: a hospital-based cross-sectional study	Hospital Anxiety and Depression Scale	≥8	yes	4
Zahidie et al. [401]	2011	Pakistan	Social environment and depression among pregnant women in rural areas of Sind, Pakistan	Centre of Epidemiological Studies-Depression	≥16	no	3
Shah et al. [402]	2011	Pakistan	Prevalence of antenatal depression: comparison between Pakistani and Canadian women	Edinburgh Postnatal Depression Scale	≥13	yes	5
Husain et al. [403]	2011	Pakistan	Prevalence and psychosocial correlates of perinatal depression: a cohort study from urban Pakistan	Edinburgh Postnatal Depression Scale	≥12	yes	5
Imran et al. [404]	2010	Pakistan	Screening of antenatal depression in Pakistan: Risk factors and effects on obstetric and neonatal outcomes	Edinburgh Postnatal Depression Scale	≥12	no	6
Muneer et al. [405]	2009	Pakistan	Frequency and associated factors for postnatal depression	Edinburgh Postnatal Depression Scale	≥12	unclear	5
Karmaliani et al. [406]	2009	Pakistan	Prevalence of anxiety, depression and associated factors among pregnant women of Hyderabad, Pakistan	Aga Khan Anxiety and Depression Scale	≥13	yes	6
Rahman et al. [407]	2007	Pakistan	Outcome of prenatal depression and risk factors associated with persistence in the first postnatal year: prospective study from Rawalpindi, Pakistan	Self Reporting Questionnaire	not specified	no	5
Kazi et al. [408]	2006	Pakistan	Social environment and depression among pregnant women in urban areas of Pakistan: importance of social relations	Centre of Epidemiological Studies-Depression	≥16	no	5

© 2023 Mitchell AR et al. *JAMA Psychiatry.*

Husain et al. [409]	2006	Pakistan	Prevalence and social correlates of postnatal depression in a low income country	Edinburgh Postnatal Depression Scale	≥12	no	5
Niaz et al. [410]	2004	Pakistan	Anxiety and depression in pregnant women presenting in the OPD of a teaching hospital	Hospital Anxiety and Depression Scale	>7	no	4
Rahman et al. [411]	2003	Pakistan	Life events, social support and depression in childbirth: perspectives from a rural community in the developing world	WHO Schedule for Clinical Assessment in Neuropsychiatry	n/a	--	4
Peru							
Carroll et al. [412]	2021	Peru	Resilience mediates the relationship between household dysfunction in childhood and postpartum depression in adolescent mothers in Peru	Patient Health Questionnaire	≥10	yes	5
Sanchez et al. [413]	2020	Peru	Association of stress-related sleep disturbance with psychiatric symptoms among pregnant women	Patient Health Questionnaire	≥10	unclear	5
Mitro et al. [414]	2020	Peru	Metabolomic markers of antepartum depression and suicidal ideation	Patient Health Questionnaire	≥10	unclear	6
Gelaye et al. [415]	2020	Peru	Association of antepartum depression, generalized anxiety, and posttraumatic stress disorder with infant birth weight and gestational age at delivery	Patient Health Questionnaire	≥10	yes	6
Friedman et al. [416]	2020	Peru	Association of social support and antepartum depression among pregnant women	Patient Health Questionnaire	≥10	yes	6
Gelaye et al. [417]	2019	Peru	Association of antepartum suicidal ideation during the third trimester with infant birth weight and gestational age at delivery	Patient Health Questionnaire	≥10	yes	4
Larrabure-Torrealva et al. [418]	2018	Peru	Prevalence and risk factors of gestational diabetes mellitus:	Patient Health Questionnaire	≥10	yes	6

© 2023 Mitchell AR et al. *JAMA Psychiatry*.

			findings from a universal screening feasibility program in Lima, Peru				
Friedman et al. [419]	2017	Peru	Migraine and the risk of post-traumatic stress disorder among a cohort of pregnant women	Patient Health Questionnaire	≥10	yes	6
Gelaye et al. [420]	2017	Peru	Trauma and traumatic stress in a sample of pregnant women	Patient Health Questionnaire	≥10	unclear	6
Gelaye et al. [421]	2017	Peru	Poor sleep quality, antepartum depression and suicidal ideation among pregnant women	Patient Health Questionnaire	≥10	unclear	6
Friedman et al. [422]	2016	Peru	Association of Migraine Headaches with Suicidal Ideation Among Pregnant Women in Lima, Peru	Patient Health Questionnaire	≥10	yes	6
Zhong et al. [423]	2016	Peru	Childhood abuse and suicidal ideation in a cohort of pregnant Peruvian women	Patient Health Questionnaire	≥10	yes	5
Yang et al. [424]	2016	Peru	Serum brain-derived neurotrophic factor (BDNF) concentrations in pregnant women with post-traumatic stress disorder and comorbid depression	Patient Health Questionnaire	≥10	yes	5
Fung et al. [425]	2015	Peru	Association of decreased serum brain-derived neurotrophic factor (BDNF) concentrations in early pregnancy with antepartum depression	Patient Health Questionnaire	≥10	yes	6
Cripe et al. [426]	2010	Peru	Depressive symptoms and migraine comorbidity among pregnant Peruvian women	Patient Health Questionnaire	≥10	yes	5
Philippines							
Labrague et al. [427]	2020	Philippines	Predictors of postpartum depression and the utilization of postpartum depression services in rural areas in the Philippines	Edinburgh Postnatal Depression Scale	>10	no	5
Russia							
Yakupova et al. [428]	2021	Russia	Postpartum Depression and Birth Experience in Russia	Edinburgh Postnatal Depression Scale	≥10	no	4

© 2023 Mitchell AR et al. *JAMA Psychiatry*.

Rwanda							
Umuziga et al. [429]	2020	Rwanda	A cross-sectional study of the prevalence and factors associated with symptoms of perinatal depression and anxiety in Rwanda	Edinburgh Postnatal Depression Scale	≥10	no	5
Niyonsenga et al. [430]	2019	Rwanda	Factors of postpartum depression among teen mothers in Rwanda: a cross- sectional study	Edinburgh Postnatal Depression Scale	≥13	no	5
Serbia							
Stojanov et al. [431]	2021	Serbia	The risk for nonpsychotic postpartum mood and anxiety disorders during the COVID-19 pandemic	Edinburgh Postnatal Depression Scale	≥10	yes	3
Odalovic et al. [432]	2015	Serbia	Translation and factor analysis of structural models of Edinburgh Postnatal Depression Scale in Serbian pregnant and postpartum women - Web-based study	Edinburgh Postnatal Depression Scale	≥13	yes	2
Dmitrovic et al. [433]	2014	Serbia	Frequency of perinatal depression in Serbia and associated risk factors	Edinburgh Postnatal Depression Scale	≥12	no	4
South Africa							
Mare et al. [434]	2021	South Africa	Perinatal suicidality: prevalence and correlates in a South African birth cohort	Edinburgh Postnatal Depression Scale	≥13	yes	6
Mal-Sarkar et al. [435]	2021	South Africa	The relationship between childhood trauma, socioeconomic status, and maternal depression among pregnant women in a South African birth cohort study	Beck Depression Inventory II	≥14	yes	5
Mokwena et al. [436]	2021	South Africa	Social and Demographic Factors Associated with Postnatal Depression Symptoms among HIV-Positive Women in Primary Healthcare Facilities, South Africa	Edinburgh Postnatal Depression Scale	≥12	yes	5
Phukuta et al. [437]	2020	South Africa	Prevalence and risk factors associated with postnatal depression in a South African primary care facility	Edinburgh Postnatal Depression Scale	>13	yes	5

© 2023 Mitchell AR et al. *JAMA Psychiatry*.

Redinger et al. [438]	2020	South Africa	Antenatal depression and anxiety across pregnancy in urban South Africa	Edinburgh Postnatal Depression Scale	≥13	yes	5
Modjadji et al. [439]	2020	South Africa	Postnatal depression screening among postpartum women attending postnatal care at selected community health centres situated in the Nkangala district of South Africa	Edinburgh Postnatal Depression Scale	≥13	yes	6
Mbatha et al. [440]	2020	South Africa	Clinical and Obstetric Risk Factors for Postnatal Depression in HIV Positive Women: A Cross Sectional Study in Health Facilities in Rural KwaZulu-Natal	Edinburgh Postnatal Depression Scale	≥12	yes	6
Mokwena et al.[441]	2020	South Africa	The Need for Universal Screening for Postnatal Depression in South Africa: Confirmation from a Sub-District in Pretoria, South Africa	Edinburgh Postnatal Depression Scale	≥13	no	6
Govender et al. [442]	2020	South Africa	Antenatal and postpartum depression: Prevalence and associated risk factors among adolescents' in KwaZulu-Natal, South Africa	Edinburgh Postnatal Depression Scale	≥13	yes	4
Duma et al. [443]	2020	South Africa	The prevalence of peripartum depression and its relationship to mode of delivery and other factors among mothers in Ixopo, Kwazulu-Natal, South Africa	Edinburgh Postnatal Depression Scale	≥10	yes	6
Pellowski et al. [444]	2019	South Africa	Perinatal depression among mothers in a South African birth cohort study: Trajectories from pregnancy to 18 months postpartum	Edinburgh Postnatal Depression Scale	≥14	yes	5
Christodoulou et al. [445]	2019	South Africa	Perinatal maternal depression in rural South Africa: Child outcomes over the first two years	Edinburgh Postnatal Depression Scale	>13	yes	5
Barnett et al. [446]	2019	South Africa	Food-insecure pregnant women in South Africa: a cross-sectional exploration of maternal depression	Edinburgh Postnatal Depression Scale	≥13	yes	5

© 2023 Mitchell AR et al. *JAMA Psychiatry*.

			as a mediator of violence and trauma risk factors				
Abrahams et al. [447]	2019	South Africa	Validation of a brief mental health screening tool for pregnant women in a low socio-economic setting	Edinburgh Postnatal Depression Scale	≥13	no	5
Mokhele et al. [448]	2019	South Africa	Prevalence and predictors of postpartum depression by HIV status and timing of HIV diagnosis in Gauteng, South Africa	Centre of Epidemiological Studies-Depression	≥5	yes	4
Peltzer et al. [449]	2018	South Africa	Prevalence of prenatal and postpartum depression and associated factors among HIV-infected women in public primary care in rural South Africa: a longitudinal study	Edinburgh Postnatal Depression Scale	≥13	yes	5
Abrahams et al. [450]	2018	South Africa	Factors associated with household food insecurity and depression in pregnant South African women from a low socio-economic setting: a cross-sectional study	Mini International Neuropsychiatric Interview	n/a	--	5
MacGinty et al. [451]	2018	South Africa	Associations between maternal mental health and early child wheezing in a South African birth cohort	Edinburgh Postnatal Depression Scale	≥13	yes	5
Van Heyningen et al. [452]	2017	South Africa	Prevalence and predictors of anxiety disorders amongst low-income pregnant women in urban South Africa: a cross-sectional study	Mini International Neuropsychiatric Interview	n/a	--	5
Tuthill et al. [453]	2017	South Africa	Perinatal Depression Among HIV-Infected Women in KwaZulu-Natal South Africa: prenatal Depression Predicts Lower Rates of Exclusive Breastfeeding	Patient Health Questionnaire	≥10	no	4
Pingo et al. [454]	2017	South Africa	Probable postpartum hypomania and depression in a South African cohort	Edinburgh Postnatal Depression Scale	≥13	yes	3

© 2023 Mitchell AR et al. *JAMA Psychiatry*.

Nydoo et al. [455]	2017	South Africa	Depressive scores in newly diagnosed HIV-infected and HIV-uninfected pregnant women	Edinburgh Postnatal Depression Scale	≥13	yes	5
Brittain et al. [456]	2017	South Africa	Social Support, Stigma and Antenatal Depression Among HIV-Infected Pregnant Women in South Africa	Edinburgh Postnatal Depression Scale	≥13	no	5
Koen et al. [457]	2017	South Africa	Maternal posttraumatic stress disorder and infant developmental outcomes in a South African birth cohort study	Edinburgh Postnatal Depression Scale	not specified	unclear	5
Tsai et al. [458]	2016	South Africa	Intimate Partner Violence and Depression Symptom Severity among South African Women during Pregnancy and Postpartum: Population-Based Prospective Cohort Study	Edinburgh Postnatal Depression Scale	≥13	yes	5
Peltzer et al. [459]	2016	South Africa	Prevalence of prenatal depression and associated factors among HIV-positive women in primary care in Mpumalanga province, South Africa	Edinburgh Postnatal Depression Scale	≥13	yes	5
Koen et al. [460]	2016	South Africa	Psychological trauma and posttraumatic stress disorder: Risk factors and associations with birth outcomes in the Drakenstein Child Health Study	Beck Depression Inventory	not specified	no	6
Heyningen et al. [461]	2016	South Africa	Antenatal depression and adversity in urban South Africa	Mini International Neuropsychiatric Interview	n/a	--	7
Tomlinson et al. [462]	2015	South Africa	Community health workers can improve child growth of antenatally-depressed, South African mothers: a cluster randomized controlled trial	Edinburgh Postnatal Depression Scale	>13	no	5
Stellenberg et al. [463]	2015	South Africa	Prevalence of and factors influencing postnatal depression in a rural community in South Africa	Edinburgh Postnatal Depression Scale	not specified	no	6

© 2023 Mitchell AR et al. *JAMA Psychiatry*.

Stein et al. [464]	2015	South Africa	Investigating the psychosocial determinants of child health in Africa: The Drakenstein Child Health Study	Beck Depression Inventory II	≥20	no	6
Choi et al. [465]	2015	South Africa	Maladaptive coping mediates the influence of childhood trauma on depression and PTSD among pregnant women in South Africa	Edinburgh Postnatal Depression Scale	≥13	yes	5
Brittain et al. [466]	2015	South Africa	Risk Factors for Antenatal Depression and Associations with Infant Birth Outcomes: Results from a South African Birth Cohort Study	Beck Depression Inventory II	≥20	unclear	5
Baron et al. [467]	2015	South Africa	Patterns of use of a maternal mental health service in a low-resource antenatal setting in South Africa	Edinburgh Postnatal Depression Scale	≥13	yes	6
Tomlinson et al. [468]	2014	South Africa	Multiple risk factors during pregnancy in South Africa: the need for a horizontal approach to perinatal care	Edinburgh Postnatal Depression Scale	>13	yes	6
Rochat et al. [469]	2013	South Africa	Detection of antenatal depression in rural HIV-affected populations with short and ultrashort versions of the Edinburgh Postnatal Depression Scale (EPDS)	Edinburgh Postnatal Depression Scale	≥13	yes	5
Dewing et al. [470]	2013	South Africa	Food insecurity and its association with co-occurring postnatal depression, hazardous drinking, and suicidality among women in peri-urban South Africa	Edinburgh Postnatal Depression Scale	≥13	yes	4
Vythilingum et al. [471]	2012	South Africa	Risk factors for substance use in pregnant women in South Africa	Edinburgh Postnatal Depression Scale	≥12	yes	4
Manikkam et al. [472]	2012	South Africa	Antenatal depression and its risk factors: an urban prevalence study in KwaZulu-Natal	Edinburgh Postnatal Depression Scale	≥13	yes	5
Rochat et al. [473]	2011	South Africa	The prevalence and clinical presentation of antenatal depression in rural South Africa	Structured Clinical Interview DSM-IV criteria	n/a	--	6

© 2023 Mitchell AR et al. *JAMA Psychiatry.*

Peltzer et al. [474]	2011	South Africa	Prevalence of postnatal depression and associated factors among HIV-positive women in primary care in Nkangala District, South Africa	Edinburgh Postnatal Depression Scale	≥14	yes	6
Hartley et al. [475]	2011	South Africa	Depressed mood in pregnancy: prevalence and correlates in two Cape Town peri-urban settlements	Edinburgh Postnatal Depression Scale	≥14	yes	6
Ramchandani et al. [476]	2009	South Africa	Predictors of postnatal depression in an urban South African cohort	Pitt Depression Questionnaire	≥20	yes	5
Cooper et al. [477]	1999	South Africa	Post-partum depression and the mother-infant relationship in a South African peri-urban settlement	Structured Clinical Interview DSM-IV criteria	n/a	--	5
Sri Lanka							
Patabendige et al. [478]	2020	Sri Lanka	Psychological impact of the COVID-19 pandemic among pregnant women in Sri Lanka	Hospital Anxiety and Depression Scale	not specified	yes	4
Fan et al. [479]	2020	Sri Lanka	Prevalence and risk factors for postpartum depression in Sri Lanka: A population-based study	Edinburgh Postnatal Depression Scale	>9	no	6
Arachchi et al. [480]	2019	Sri Lanka	Suicidal ideation and intentional self-harm in pregnancy as a neglected agenda in maternal health; an experience from rural Sri Lanka	Edinburgh Postnatal Depression Scale	>9	no	5
Herath et al. [481]	2017	Sri Lanka	Physical and psychological morbidities among selected antenatal females in Kegalle district of Sri Lanka: A cross sectional study	Edinburgh Postnatal Depression Scale	>9	yes	6
Agampodi et al. [482]	2013	Sri Lanka	Antenatal depression in Anuradhapura, Sri Lanka and the factor structure of the Sinhalese version of Edinburgh post partum depression scale among pregnant women	Edinburgh Postnatal Depression Scale	>9	yes	6
Sudan							
Khalifa et al. [483]	2018	Sudan	Course of depression symptoms between 3 and 8 months after	Edinburgh Postnatal Depression Scale	≥12	yes	4

© 2023 Mitchell AR et al. *JAMA Psychiatry*.

			delivery using two screening tools (EPDS and HSCL-10) on a sample of Sudanese women in Khartoum state				
Khalifa et al. [484]	2016	Sudan	Determinants of postnatal depression in Sudanese women at 3 months postpartum: a cross-sectional study	Edinburgh Postnatal Depression Scale	≥12	no	5
Khalifa et al. [485]	2015	Sudan	Postnatal depression among Sudanese women: Prevalence and validation of the Edinburgh Postnatal depression scale at 3 months postpartum	Edinburgh Postnatal Depression Scale	≥12	no	4
Syria							
Roumieh et al. [486]	2019	Syria	Prevalence and risk factors for postpartum depression among women seen at Primary Health Care Centres in Damascus	Edinburgh Postnatal Depression Scale	≥13	no	5
Tanzania							
Manongi et al. [487]	2020	Tanzania	The Association Between Intimate Partner Violence and Signs of Depression During Pregnancy in Kilimanjaro Region, Northern Tanzania	Edinburgh Postnatal Depression Scale	≥13	no	4
Herlosky et al. [488]	2020	Tanzania	Postpartum Maternal Mood Among Hadza Foragers of Tanzania: A Mixed Methods Approach	Edinburgh Postnatal Depression Scale	>12	no	5
Ngocho et al. [489]	2019	Tanzania	Depression and anxiety among pregnant women living with HIV in Kilimanjaro region, Tanzania	Edinburgh Postnatal Depression Scale	≥10	no	6
Holm-Larsen et al. [490]	2019	Tanzania	Postpartum depression and child growth in Tanzania: a cohort study	Edinburgh Postnatal Depression Scale	≥13	no	6
Mahenge et al. [491]	2018	Tanzania	Adverse childhood experiences and intimate partner violence during pregnancy and their association to postpartum depression	Patient Health Questionnaire	≥9	yes	6
Rogathi et al. [492]	2017	Tanzania	Postpartum depression among women who have experienced	Edinburgh Postnatal Depression Scale	≥13	yes	6

© 2023 Mitchell AR et al. *JAMA Psychiatry*.

			intimate partner violence: A prospective cohort study at Moshi, Tanzania				
Kaaya et al. [493]	2016	Tanzania	Association of maternal depression and infant nutritional status among women living with HIV in Tanzania	John Hopkins Symptoms Checklist	>1.06	yes	5
Rwakarema et al. [494]	2015	Tanzania	Antenatal depression is associated with pregnancy-related anxiety, partner relations, and wealth in women in Northern Tanzania: a cross-sectional study	Edinburgh Postnatal Depression Scale	≥13	no	5
Mahenge et al. [495]	2015	Tanzania	The prevalence of mental health morbidity and its associated factors among women attending a prenatal clinic in Tanzania	John Hopkins Symptoms Checklist	>1.06	yes	4
Mahenge et al. [496]	2013	Tanzania	Intimate partner violence during pregnancy and associated mental health symptoms among pregnant women in Tanzania: a cross-sectional study	John Hopkins Symptoms Checklist	≥18	yes	5
Kaaya et al. [497]	2010	Tanzania	Socio-economic and partner relationship factors associated with antenatal depressive morbidity among pregnant women in Dar es Salaam, Tanzania	John Hopkins Symptoms Checklist	>1.06	yes	5
Thailand							
Tuksanawes et al. [498]	2020	Thailand	Prevalence and associated factors of antenatal depressive symptoms in pregnant women living in an urban area of Thailand	Centre of Epidemiological Studies-Depression	≥19	yes	5
Fellmeth et al. [499]	2020	Thailand	Prevalence and determinants of perinatal depression among labour migrant and refugee women on the Thai-Myanmar border: a cohort study	Structured Clinical Interview DSM-IV criteria	n/a	--	6
Phoosuwan et al. [500]	2018	Thailand	Antenatal depressive symptoms during late pregnancy among	Edinburgh Postnatal Depression Scale	≥10	yes	5

© 2023 Mitchell AR et al. *JAMA Psychiatry*.

			women in a north-eastern province of Thailand: Prevalence and associated factors				
Hassert et al. [501]	2018	Thailand	Postpartum Depressive Symptoms: Risks for Czech and Thai Mothers	Edinburgh Postnatal Depression Scale	≥12	no	4
Roomruangwong et al. [502]	2016	Thailand	Antenatal and postnatal risk factors of postpartum depression symptoms in Thai women: A case-control study	Edinburgh Postnatal Depression Scale	≥11	yes	5
Uthaipaisanwong et al. [503]	2015	Thailand	Associated factors of prenatal depression among teenage pregnant women at King Chulalongkorn Memorial Hospital	Edinburgh Postnatal Depression Scale	≥11	yes	5
Panyayong [504]	2013	Thailand	Postpartum depression among Thai women: a national survey	Edinburgh Postnatal Depression Scale	≥13	yes	5
Ross et al. [505]	2011	Thailand	Depressive symptoms among HIV-positive postpartum women in Thailand	Centre of Epidemiological Studies-Depression	≥16	yes	4
Ross et al. [506]	2009	Thailand	Depressive symptoms among HIV-positive pregnant women in Thailand	Centre of Epidemiological Studies-Depression	≥16	yes	5
Liabsuetrakul et al. [507]	2007	Thailand	Clinical applications of anxiety, social support, stressors, and self-esteem measured during pregnancy and postpartum for screening postpartum depression in Thai women	Psychiatrist interview with DSM-IV criteria	n/a	--	5
Limlomwongse et al. [508]	2006	Thailand	Cohort study of depressive moods in Thai women during late pregnancy and 6-8 weeks of postpartum using the Edinburgh Postnatal Depression Scale (EPDS)	Edinburgh Postnatal Depression Scale	≥10	unclear	5
Timor-Leste							
Rees et al. [509]	2016	Timor-Leste	A high-risk group of pregnant women with elevated levels of conflict-related trauma, intimate partner violence, symptoms of depression and other forms of	Edinburgh Postnatal Depression Scale	≥13	no	6

© 2023 Mitchell AR et al. *JAMA Psychiatry*.

			mental distress in post-conflict Timor-Leste				
Silove et al. [510]	2015	Timor-Leste	Pathways to perinatal depressive symptoms after mass conflict in Timor-Leste: a modelling analysis using cross-sectional data	Edinburgh Postnatal Depression Scale	≥13	no	6
Turkey							
Guvenc et al. [511]	2021	Turkey	Anxiety, depression, and knowledge level in postpartum women during the COVID-19 pandemic	Edinburgh Postnatal Depression Scale	≥13	yes	4
Boran et al. [512]	2020	Turkey	Screening of postpartum depression among new mothers in Istanbul: a psychometric evaluation of the Turkish Edinburgh Postnatal Depression Scale	Edinburgh Postnatal Depression Scale	not specified	yes	5
Sut et al. [513]	2020	Turkey	Anxiety, depression, and related factors in pregnant women during the COVID-19 pandemic in Turkey: A web-based cross-sectional study	Hospital Anxiety and Depression Scale	≥8	yes	3
Oskovi-Kaplan et al. [514]	2020	Turkey	The Effect of COVID-19 Pandemic and Social Restrictions on Depression Rates and Maternal Attachment in Immediate Postpartum Women: a Preliminary Study	Edinburgh Postnatal Depression Scale	≥13	yes	4
Kızılırmak et al. [515]	2020	Turkey	Correlation between postpartum depression and spousal support and factors affecting postpartum depression	Edinburgh Postnatal Depression Scale	≥12	yes	4
Cankaya [516]	2020	Turkey	The effect of psychosocial risk factors on postpartum depression in antenatal period: A prospective study	Edinburgh Postnatal Depression Scale	≥13	yes	5
Aydemir et al. [517]	2020	Turkey	The Relationship Between Maternal Self-confidence and Postpartum	Edinburgh Postnatal Depression Scale	≥13	unclear	6

© 2023 Mitchell AR et al. *JAMA Psychiatry*.

			Depression in Primipara Mothers: A Follow-Up Study				
Anık et al. [518]	2020	Turkey	The Relationship between psychosocial health status and risk of depression among pregnant women in Turkey	Edinburgh Postnatal Depression Scale	≥13	yes	5
Topatan et al. [519]	2019	Turkey	Frequency of Depression and Risk Factors among Adolescent Mothers in Turkey within the First Year of the Postnatal Period	Edinburgh Postnatal Depression Scale	≥12	yes	4
Sahin et al. [520]	2019	Turkey	Depressive symptoms during pregnancy and postpartum: a prospective cohort study	Beck Depression Inventory	>17	yes	5
Oztora et al. [521]	2019	Turkey	Postpartum depression and affecting factors in primary care	Edinburgh Postnatal Depression Scale	≥12	yes	5
Nacar et al. [522]	2019	Turkey	Relationship between sleep characteristics and depressive symptoms in last trimester of pregnancy	Beck Depression Inventory	≥17	yes	5
Unsal Atan et al. [523]	2018	Turkey	Relation between mothers' types of labor, birth interventions, birth experiences and postpartum depression: A multicentre follow-up study	Edinburgh Postnatal Depression Scale	≥13	yes	5
Duman et al. [524]	2018	Turkey	Prospective associations between recalled parental bonding and perinatal depression: a cohort study in urban and rural Turkey	Edinburgh Postnatal Depression Scale	≥13	yes	2
Capik et al. [525]	2018	Turkey	Fear of Childbirth, Postpartum Depression, and Birth-Related Variables as Predictors of Posttraumatic Stress Disorder After Childbirth	Edinburgh Postnatal Depression Scale	≥13	yes	5
Yildiz et al. [526]	2017	Turkey	Serum serotonin, leptin, and adiponectin changes in women with postpartum depression: controlled study	Edinburgh Postnatal Depression Scale	≥13	yes	4

© 2023 Mitchell AR et al. *JAMA Psychiatry*.

Dikmen-Yildiz et al. [527]	2017	Turkey	Depression, anxiety, PTSD and comorbidity in perinatal women in Turkey: A longitudinal population-based study	Edinburgh Postnatal Depression Scale	≥14	yes	6
Cankorur et al. [528]	2017	Turkey	Gender preference and perinatal depression in Turkey: A cohort study	Edinburgh Postnatal Depression Scale	≥13	yes	4
Celik et al. [529]	2016	Turkey	Screening mixed depression and bipolarity in the postpartum period at a primary health care center	Edinburgh Postnatal Depression Scale	≥13	yes	4
Bolak Boratav et al. [530]	2016	Turkey	Postpartum depression and its psychosocial correlates: A longitudinal study among a group of women in Turkey	Edinburgh Postnatal Depression Scale	>12	yes	5
Turkcapar et al. [531]	2015	Turkey	Sociodemographic and clinical features of postpartum depression among Turkish women: a prospective study	Edinburgh Postnatal Depression Scale	≥13	yes	4
Aktas et al. [532]	2015	Turkey	Factors affecting depression during pregnancy and the correlation between social support and pregnancy depression	Beck Depression Inventory	≥17	yes	5
Kirkan et al. [533]	2015	Turkey	The depression in women in pregnancy and postpartum period: A follow-up study	Structured Clinical Interview DSM-IV criteria	n/a	--	5
AkcaliAslan et al. [534]	2014	Turkey	Prevalence of depressive disorders and related factors in women in the first trimester of their pregnancies in Erzurum, Turkey	Edinburgh Postnatal Depression Scale	>12	yes	4
Pocan et al. [535]	2013	Turkey	The incidence of and risk factors for postpartum depression at an urban maternity clinic in Turkey	Edinburgh Postnatal Depression Scale	≥13	yes	6
Orun et al. [536]	2013	Turkey	Relations of maternal psychopathologies, social-obstetrical factors and mother-infant bonding at 2-month postpartum: a sample of Turkish mothers	Edinburgh Postnatal Depression Scale	>12	yes	5

© 2023 Mitchell AR et al. *JAMA Psychiatry*.

Yanikkerem et al. [537]	2013	Turkey	Antenatal depression: prevalence and risk factors in a hospital based Turkish sample	Beck Depression Inventory	≥17	yes	5
Serhan et al. [538]	2013	Turkey	Prevalence of postpartum depression in mothers and fathers and its correlates	Edinburgh Postnatal Depression Scale	≥13	unclear	4
Senturk et al. [539]	2011	Turkey	Social support and antenatal depression in extended and nuclear family environments in Turkey: A cross-sectional survey	Edinburgh Postnatal Depression Scale	≥13	yes	6
Ozbasaran et al. [540]	2011	Turkey	Prevalence and risk factors concerning postpartum depression among women within early postnatal periods in Turkey	Edinburgh Postnatal Depression Scale	≥13	yes	6
Bodur et al. [541]	2010	Turkey	Risk factors for postpartum depression in a group of teenage mothers	Edinburgh Postnatal Depression Scale	≥13	yes	5
Yagmur et al. [542]	2010	Turkey	Social support and postpartum depression in low-socioeconomic level postpartum women in Eastern Turkey	Edinburgh Postnatal Depression Scale	≥13	yes	5
Kirpinar et al. [543]	2010	Turkey	Prospective study of postpartum depression in eastern Turkey prevalence, socio-demographic and obstetric correlates, prenatal anxiety and early awareness	Edinburgh Postnatal Depression Scale	≥13	yes	4
Golbasi et al. [544]	2010	Turkey	Prevalence and correlates of depression in pregnancy among Turkish women	Edinburgh Postnatal Depression Scale	≥13	yes	3
Karacam et al. [545]	2009	Turkey	Depression, anxiety and influencing factors in pregnancy: a study in a Turkish population	Beck Depression Inventory	≥17	yes	6
Ege et al. [546]	2008	Turkey	Social support and symptoms of postpartum depression among new mothers in Eastern Turkey	Edinburgh Postnatal Depression Scale	≥13	yes	5
Kara et al. [547]	2008	Turkey	Is there a role for the family and close community to help reduce the risk of postpartum depression in	Beck Depression Inventory	≥17	yes	6

© 2023 Mitchell AR et al. *JAMA Psychiatry*.

			new mothers? A cross-sectional study of Turkish women				
Caliskan et al. [548]	2007	Turkey	Depression scores and associated factors in pregnant and non-pregnant women: a community-based study in Turkey	Beck Depression Inventory	≥18	yes	4
Akman et al. [549]	2007	Turkey	Postpartum-onset major depression is associated with personality disorders	Structured Clinical Interview DSM-IV criteria	n/a	--	5
Dindar et al. [550]	2007	Turkey	Screening of Turkish women for postpartum depression within the first postpartum year: the risk profile of a community sample	Edinburgh Postnatal Depression Scale	≥9	yes	5
Gulseren et al. [551]	2006	Turkey	From antepartum to postpartum: a prospective study on the prevalence of peripartum depression in a semiurban Turkish community	Edinburgh Postnatal Depression Scale	≥10	yes	5
Ozdemir et al. [552]	2005	Turkey	Postnatal depressive mood in Turkish women	Self Rating Depression Scale	≥50	no	5
Aydin et al. [553]	2005	Turkey	Depression and associated factors among women within their first postnatal year in Erzurum province in eastern Turkey	Edinburgh Postnatal Depression Scale	≥12	yes	5
Inandi et al. [554]	2005	Turkey	Risk factors for depression in the first postnatal year: a Turkish study	Edinburgh Postnatal Depression Scale	≥13	yes	5
Bugdayci et al. [555]	2004	Turkey	A cross-sectional prevalence study of depression at various times after delivery in Mersin province in Turkey	Edinburgh Postnatal Depression Scale	>12	yes	6
Aydin et al. [556]	2004	Turkey	Validation of the Turkish version of the Edinburgh Postnatal Depression Scale among women within their first postpartum year	Structured Clinical Interview DSM-IV criteria	n/a	--	5
Ekuklu et al. [557]	2004	Turkey	Prevalence of postpartum depression in Edirne, Turkey, and related factors	Edinburgh Postnatal Depression Scale	≥12	no	2

© 2023 Mitchell AR et al. *JAMA Psychiatry*.

Inandi et al. [558]	2002	Turkey	Risk factors for depression in postnatal first year, in eastern Turkey	Edinburgh Postnatal Depression Scale	≥13	yes	5
Danaci et al. [559]	2002	Turkey	Postnatal depression in turkey: epidemiological and cultural aspects	Edinburgh Postnatal Depression Scale	≥13	yes	5
Uganda							
Arach et al. [560]	2020	Uganda	Perinatal death triples the prevalence of postpartum depression among women in Northern Uganda: A community-based cross-sectional study	Edinburgh Postnatal Depression Scale	≥14	no	6
Nampijja et al. [561]	2019	Uganda	The burden and risk factors for postnatal depression and depressive symptomatology among women in Kampala	Mini International Neuropsychiatric Interview	n/a	--	7
Natamba et al. [562]	2014	Uganda	Reliability and validity of the center for epidemiologic studies-depression scale in screening for depression among HIV-infected and -uninfected pregnant women attending antenatal services in northern Uganda: a cross-sectional study	Mini International Neuropsychiatric Interview	n/a	--	6
Kakyo et al. [563]	2012	Uganda	Factors associated with depressive symptoms among postpartum mothers in a rural district in Uganda	Edinburgh Postnatal Depression Scale	≥10	no	5
Ukraine							
Bailey et al.[564]	2016	Ukraine	Prevalence of depressive symptoms in pregnant and postnatal HIV-positive women in Ukraine: A cross-sectional survey	Patient Health Questionnaire	≥1	no	3
Vietnam							
Luong-Thanh et al. [565]	2021	Vietnam	Depression and its associated factors among pregnant women in central Vietnam	Patient Health Questionnaire	≥10	no	6
Hue et al. [566]	2020	Vietnam	Factors associated with antenatal depression among pregnant women	Edinburgh Postnatal Depression Scale	≥10	unclear	5

© 2023 Mitchell AR et al. *JAMA Psychiatry*.

			in Vietnam: A multisite cross-sectional survey				
Tho Nhi et al. [567]	2019	Vietnam	Intimate Partner Violence among Pregnant Women and Postpartum Depression in Vietnam: A Longitudinal Study	Edinburgh Postnatal Depression Scale	≥10	yes	5
Van Ngo et al. [568]	2018	Vietnam	Antenatal depressive symptoms and adverse birth outcomes in Hanoi, Vietnam	Edinburgh Postnatal Depression Scale	≥10	no	4
Tho Tran et al. [569]	2018	Vietnam	Emotional violence exerted by intimate partners and postnatal depressive symptoms among women in Vietnam: A prospective cohort study	Edinburgh Postnatal Depression Scale	≥10	yes	6
Vo et al. [570]	2017	Vietnam	Postpartum Depressive Symptoms and Associated Factors in Married Women: A Cross-sectional Study in Danang City, Vietnam	Edinburgh Postnatal Depression Scale	≥13	no	5
Murray et al. [571]	2015	Vietnam	Postnatal depressive symptoms amongst women in Central Vietnam: a cross-sectional study investigating prevalence and associations with social, cultural and infant factors	Edinburgh Postnatal Depression Scale	≥13	yes	6
Suzuki et al. [572]	2011	Vietnam	Postnatal depression and associated parenting indicators among Vietnamese women	Whooley Questions	≥1	no	5
Fisher et al. [573]	2010	Vietnam	Common perinatal mental disorders in northern Viet Nam: community prevalence and health care use	Structured Clinical Interview DSM-IV criteria	n/a	--	7
Fisher et al. [574]	2007	Vietnam	Relative socioeconomic advantage and mood during advanced pregnancy in women in Vietnam	Edinburgh Postnatal Depression Scale	>9	yes	5
Fisher et al. [575]	2004	Vietnam	Prevalence, nature, severity and correlates of postpartum depressive symptoms in Vietnam	Edinburgh Postnatal Depression Scale	≥13	no	6

© 2023 Mitchell AR et al. *JAMA Psychiatry*.

West Bank							
Qandil et al. [576]	2016	West Bank	Postpartum depression in the Occupied Palestinian Territory: a longitudinal study in Bethlehem	Edinburgh Postnatal Depression Scale	≥11	no	6
Zambia							
Ndokera et al. [577]	2011	Zambia	The relationship between maternal depression and adverse infant health outcomes in Zambia: a cross-sectional feasibility study	Self Reporting Questionnaire	≥8	no	6
Zimbabwe							
Kaiyo-Utete [578]	2020	Zimbabwe	Study of depression and anxiety in prenatal and postnatal women at Port Moresby General Hospital	Structured Clinical Interview DSM-IV criteria	n/a	--	4
Nyamukoho et al. [579]	2019	Zimbabwe	Depression among HIV positive pregnant women in Zimbabwe: a primary health care based cross-sectional study	Edinburgh Postnatal Depression Scale	≥12	yes	4
January et al. [580]	2018	Zimbabwe	Prevalence and factors associated with postnatal depression among women in two rural districts of Manicaland, Zimbabwe	Structured Clinical Interview DSM-IV criteria	n/a	--	6
January et al. [581]	2017	Zimbabwe	Correlates of postnatal depression among women in Zimbabwean semi-urban and rural settings	Edinburgh Postnatal Depression Scale	≥12	yes	6
Shamu et al. [582]	2016	Zimbabwe	High-frequency intimate partner violence during pregnancy, postnatal depression and suicidal tendencies in Harare, Zimbabwe	Centre of Epidemiological Studies-Depression	≥8	no	6
January et al. [583]	2015	Zimbabwe	Prevalence and the correlates of postnatal depression in an urban high density suburb of Harare	Edinburgh Postnatal Depression Scale	≥12	yes	4
Chibanda et al. [584]	2010	Zimbabwe	Postnatal depression by HIV status among women in Zimbabwe	Structured Clinical Interview DSM-IV criteria	n/a	--	7
Multinational							
Wesselhoeft et al. [585]	2020	Multinational	Postnatal depressive symptoms display marked similarities across continents	Edinburgh Postnatal Depression Scale	>12	Vietnam- yes<br>Tanzania- no	5

© 2023 Mitchell AR et al. *JAMA Psychiatry*.

McCauley et al. [586]	2018	Multinational	Burden of physical, psychological and social ill-health during and after pregnancy among women in India, Pakistan, Kenya and Malawi	Edinburgh Postnatal Depression Scale	≥10	India- unclear<br>Pakistan- unclear<br>Kenya- unclear<br>Malawi- unclear	5
Bindt et al. [587]	2013	Multinational	No association between antenatal common mental disorders in low-obstetric risk women and adverse birth outcomes in their offspring: results from the CDS study in Ghana and Cote D'Ivoire	Patient Health Questionnaire	≥10	Ghana- no<br>Cote D'Ivoire- no	6
Guo et al. [588]	2013	Multinational	Association of antepartum and postpartum depression in Ghanaian and Ivorian women with febrile illness in their offspring: A prospective birth cohort study	Patient Health Questionnaire	≥10	Ghana- no<br>Cote D'Ivoire- no	5
Bindt et al. [589]	2012	Multinational	Antepartum Depression and Anxiety Associated with Disability in African Women: Cross-Sectional Results from the CDS Study in Ghana and Cote d'Ivoire	Patient Health Questionnaire	≥10	Ghana- no<br>Cote D'Ivoire- no	6

© 2023 Mitchell AR et al. *JAMA Psychiatry*.

eTable 3. Prevalence of perinatal depression by method of assessment, local validation, study design, setting and risk of bias score

	No. of studies	Total no. of participants	No. of participants with depression	Weight	Prevalence of depressive symptoms (95% CI)	Heterogeneity (I^2)
**Method of assessment**						
Self-reported screening	537	598 054	121 102	91.2	25.3 (24.3 – 26.3)	99.2
Diagnostic interview	51	18 123	3 219	8.7	17.8 (15.4 – 20.1)	96.0
Self-reported screening + diagnostic interview	1	531	116	0.2	21.9 (18.5 – 25.6)	NA
**Diagnostic/screening tool locally validated**						
Yes	387	469 026	91 255	66.7	23.7 (22.6 – 24.7)	99.2
No	202	147 682	33 182	33.3	26.7 (24.7 – 28.7)	99.2
**Study design**						
Cross sectional	392	349 620	75 972	61.8	26.4 (25.0 – 27.7)	99.3
Prospective cohort	182	231 346	43 091	35.7	21.6 (20.3 – 22.9)	98.8
Randomised Control Trial	8	31 016	4 017	1.2	26.1 (19.9 – 32.3)	99.7
Retrospective cohort	4	2 628	906	0.6	33.2 (23.1 – 43.3)	96.3
Case-control study	3	2 098	451	0.6	21.7 (18.1 – 25.4)	73.2
**Setting**						
Teaching hospital	106	72 836	20 45	17.5	26.4 (23.56 – 29.2)	98.9
Secondary hospital	139	160 353	26 297	24.3	22.5 (20.9 – 24.2)	99.1
Community/Primary health	256	192 711	41 733	42.0	25.7 (24.1 – 26.6)	99.1
Population-based	37	122 686	24 157	6.5	23.8 (21.0 – 26.6)	99.4
Combination	51	68 122	12 205	9.7	23.2 (20.0 – 26.5)	99.4
**Risk of bias score***						
1	3	850	217	0.5	43.2 (-25.4 – 111.8)	NA
2	11	7 882	2 051	2.1	26.7 (18.4 – 35.0)	98.9
3	20	24 280	4 625	3.6	24.9 (20.0 – 29.8)	98.2
4	126	97 199	24 908	22.2	25.83 (23.3 – 28.4)	99.1
5	249	249 613	51 476	42.4	25.0 (23.5 – 26.4)	99.1
6	166	230 773	40 171	26.9	23.5 (22.0 – 25.0)	99.2
7	14	5893	868	2.4	15.5 (11.5 – 19.5)	96.2
**Risk of bias category**						
Higher risk of bias (score 1,2,3,4)	160	130 211	31 801	28.3	26.1 (23.8 – 28.3)	99.2
Lower risk of bias (score 5,6,7)	429	486 497	92 636	71.7	24.1 (23.1 – 25.1)	99.1

*on adapted Newcastle-Ottawa Scale

© 2023 Mitchell AR et al. *JAMA Psychiatry.*

eTable 4. Prevalence of perinatal depression by the three most commonly used self-reported screening instruments

Self-Reported Screening Tool	No. of studies	Total no. of participants	No. of participants with depression	Weight	Prevalence of depressive symptoms (95% CI)	Heterogeneity (I^2)
Edinburgh Postnatal Depression Scale (symptoms previous 7 days)	384	328 984	68 780	65.44	24.3 (23.3 – 25.3)	98.5
Patient Health Questionnaire (symptoms previous 14 days)	59	201 665	31 395	10.26	23.0 (20.7 – 25.2)	99.6
Beck Depression Inventory (symptoms previous 7 days)	21	9 812	3800	3.17	29.8 (18.0 – 41.7)	99.6

eTable 5. Prevalence of perinatal depression with the three most commonly used diagnostic interviews

Diagnostic Interview	No. of studies	Total no. of participants	No. of participants with depression	Weight	Prevalence of depressive symptoms (95% CI)	Heterogeneity (I^2)
Mini International Neuropsychiatric Interview	21	7056	1074	3.3	15.7 (12.1 – 19.3)	96.2
Structured Clinical Interview DSM-IV criteria	16	5208	872	2.9	18.1 (14.3 – 21.9)	94.3
Interview with ICD-10 criteria	3	1143	203	0.61	17.6 (12.2 – 23.0)	83.5

© 2023 Mitchell AR et al. *JAMA Psychiatry.*

1.

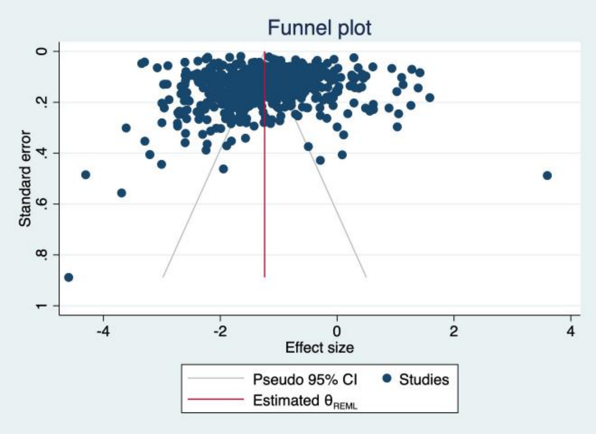

Egger's test: p-value <0.001

eFigures 1 – 13: Funnel Plots and Egger's test

1. All studies
2. Low-income countries
3. Lower-Middle Income Countries
4. Upper-Middle Income Countries
5. East Asia and the Pacific Region
6. Europe and Central Asia Region
7. Latin America and the Caribbean Region
8. Middle-East and North Africa Region
9. South Asia Region
10. Sub-Saharan Africa Region
11. Antenatal Period
12. Postnatal Period
13. Perinatal (Antenatal and Postnatal Combined) Period

2.

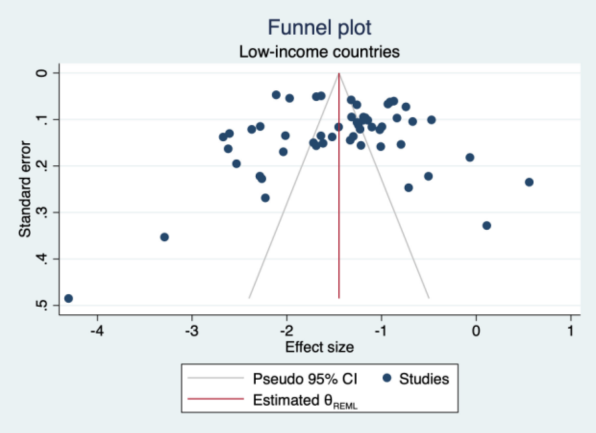

Egger's test: p-value 0.01

3.

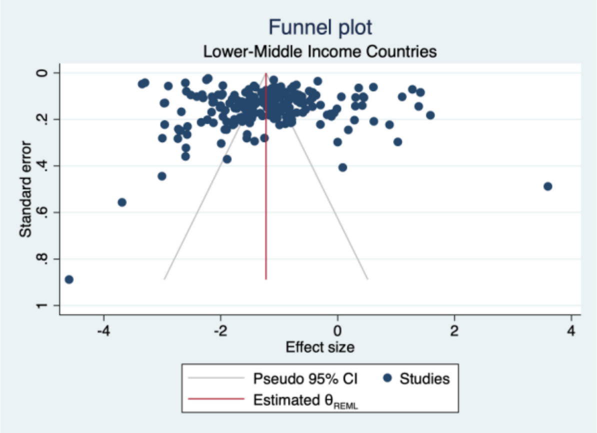

Egger's test: p-value 0.05

4.

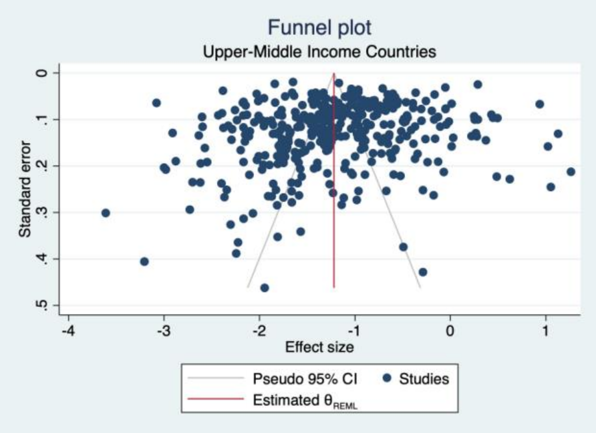

Egger's test: p-value <0.001

© 2023 Mitchell AR et al. *JAMA Psychiatry*.

5.

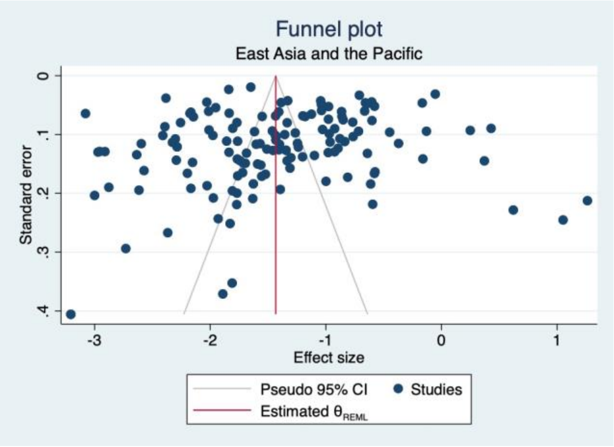

Egger's test: p-value <0.001

6.

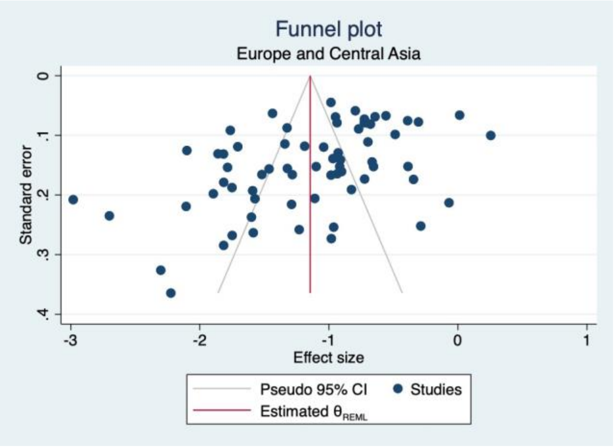

Egger's test: p-value <0.001

7.

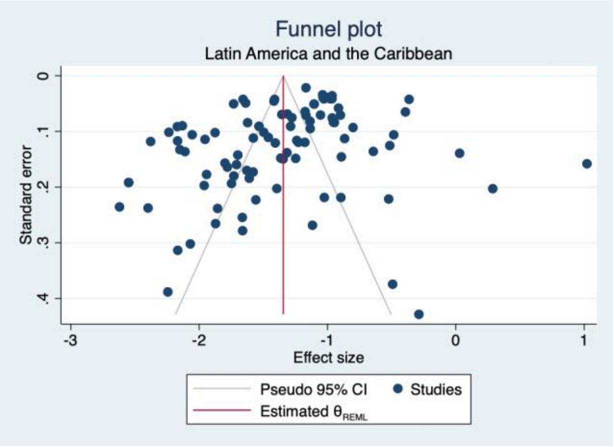

Egger's test: p-value 0.04

8.

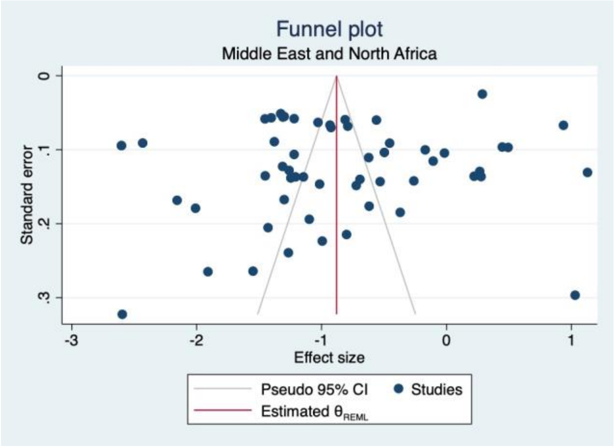

Egger's test: p-value 0.40

9.

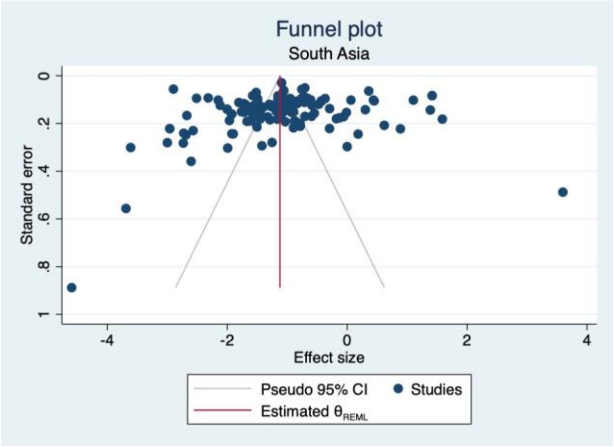

Egger's test: p-value 0.003

10.

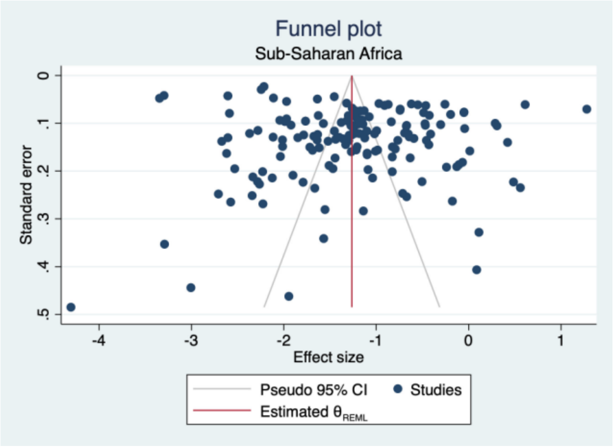

Egger's test: p-value 0.06

© 2023 Mitchell AR et al. *JAMA Psychiatry*.

11.

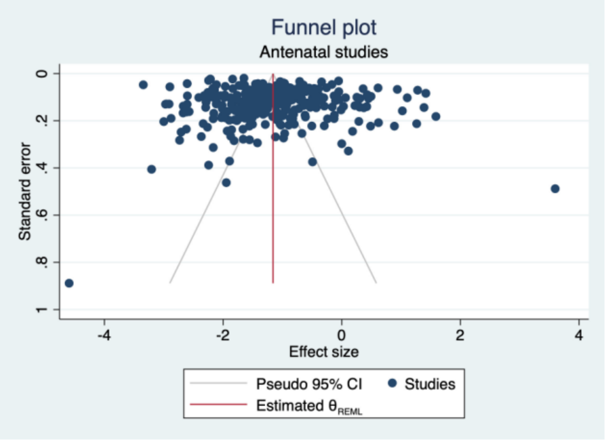

Egger's test: p-value 0.14

12.

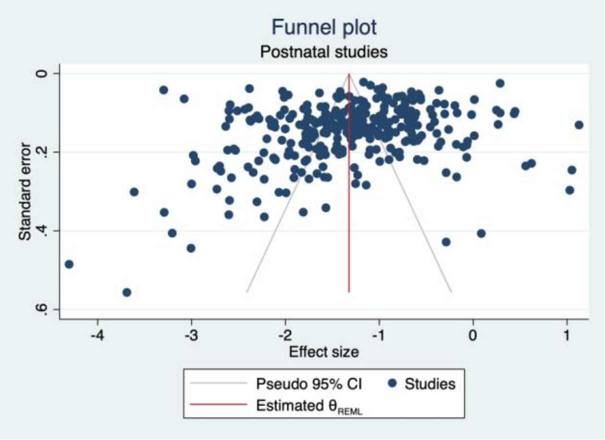

Egger's test: p-value <0.001

13.

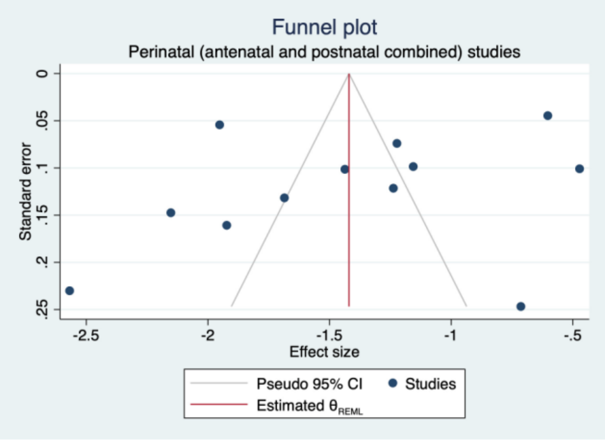

Egger's test: p-value 0.28

© 2023 Mitchell AR et al. *JAMA Psychiatry*.

eReferences

1. Cela M, Kola V. An evaluation of postpartum depression - a study in Albania for 2015-2017 period. International Journal of Ecosystems and Ecology Science-Ijees. 2019;9(1):183-8.
2. Meçe D. Relationship between postpartum depression, infant temperament and childcare stress in Tirana, Albania. International Journal of Child Health & Human Development. 2014;7(1):49-53.
3. Cormick G, Puppo S, Vazquez PF, Gibbons L, Rodriguez R, Bosio MV, et al. Factors associated with depression during pregnancy in women from a low socioeconomic level: A hierarchical model approach. Psychiatry Research. 2021;298:113798.
4. Pham D, Cormick G, Amyx MM, Gibbons L, Doty M, Brown A, et al. Factors associated with postpartum depression in women from low socioeconomic level in Argentina: A hierarchical model approach. Journal of Affective Disorders. 2018;227:731-8.
5. Mathisen SE, Glavin K, Lien L, Lagerlov P. Prevalence and risk factors for postpartum depressive symptoms in Argentina: a cross-sectional study. International Journal of Women's Health. 2013;5:787-93.
6. Tasnim F, Rahman M, Islam MM, Hasan M, Mostofa MG, Rahman MM. Exposure to domestic violence and the risk of developing depression within 6 months postpartum in Bangladesh. Social Psychiatry & Psychiatric Epidemiology. 2021;03:03.
7. Valdes V, Berens AE, Nelson CA. Socioeconomic and psychological correlates of postpartum depression at 6 months in Dhaka, Bangladesh. International journal of psychology : Journal international de psychologie. 2020;16.
8. Begum MR, Biswas SC. Changes in Depressive Symptoms During the Antenatal Period: A Cohort Study from Bangladesh. Indian Journal of Psychological Medicine. 2020;42(6):519-24.
9. Islam MJ, Broidy L, Mazerolle P, Baird K, Mazumder N, Zobair KM. Do Maternal Depression and Self-Esteem Moderate and Mediate the Association Between Intimate Partner Violence After Childbirth and Postpartum Suicidal Ideation? Archives of Suicide Research. 2020;24(4):609-32.
10. Azad R, Fahmi R, Shrestha S, Joshi H, Hasan M, Khan ANS, et al. Prevalence and risk factors of postpartum depression within one year after birth in urban slums of Dhaka, Bangladesh. PLoS ONE [Electronic Resource]. 2019;14(5):e0215735.
11. Sharmin KN, Sarwar N, Mumu SJ, Taleb DA, Flora MS. Postnatal depression and infant growth in an urban area of Bangladesh. Midwifery. 2019;74:57-67.
12. Nasreen HE, Kabir ZN, Forsell Y, Edhborg M. Impact of maternal depressive symptoms and infant temperament on early infant growth and motor development: Results from a population based study in Bangladesh. Journal of Affective Disorders. 2013;146(2):254-61.
13. Edhborg M, Nasreen HE, Kabir ZN. Impact of postpartum depressive and anxiety symptoms on mothers' emotional tie to their infants 2-3 months postpartum: a population-based study from rural Bangladesh. Archives of Women's Mental Health. 2011;14(4):307-16.
14. Nasreen HE, Kabir ZN, Forsell Y, Edhborg M. Prevalence and associated factors of depressive and anxiety symptoms during pregnancy: A population based study in rural Bangladesh. BMC Women's Health. 2011;11(1):22-.
15. Nasreen HE, Kabir ZN, Forsell Y, Edhborg M. Low birth weight in offspring of women with depressive and anxiety symptoms during pregnancy: results from a population based study in Bangladesh. BMC Public Health. 2010;10(1):515-.
16. Gausia K, Fisher C, Ali M, Oosthuizen J. Antenatal depression and suicidal ideation among rural Bangladeshi women: a community-based study. Archives of Women's Mental Health. 2009;12(5):351-8.
17. Gausia K, Fisher C, Ali M, Oosthuizen J. Magnitude and contributory factors of postnatal depression: a community-based cohort study from a rural subdistrict of Bangladesh. Psychological Medicine. 2009;39(6):999-1007.
18. Santos IS, Munhoz TN, Blumenberg C, Barcelos R, Bortolotto CC, Matijasevich A, et al. Post-partum depression: a cross-sectional study of women enrolled in a conditional cash transfer program in 30 Brazilian cities. Journal of Affective Disorders. 2021;281:510-6.
19. Soares MC, de Matos MB, da Cunha GK, Leite CF, Caruccio HS, Trettim JP, et al. Suicide risk and prematurity: A study with pregnant adolescents. Journal of Psychiatric Research. 2021;133:125-33.
20. de Mello DB, Trettim JP, da Cunha GK, Rubin BB, Scholl CC, Ardais AP, et al. Generalized Anxiety Disorder, Depressive Symptoms and the Occurrence of Stressors Events in a Probabilistic Sample of Pregnant Women. Psychiatric Quarterly. 2021;92(1):123-33.

© 2023 Mitchell AR et al. *JAMA Psychiatry.*

21. Ribeiro RAB, Rubin BB, Castelli RD, de Matos MB, Coelho FT, da Cunha Coelho FM, et al. Childhood trauma and depressive symptoms in pregnant adolescents in Southern Brazil. International Journal of Public Health. 2020;65(2):197-205.
22. Pabon S, Parpinelli MA, Narvaez MB, Charles CM, Guida JP, Escobar MF, et al. Overall Maternal Morbidity during Pregnancy Identified with the WHO-WOICE Instrument. Biomed Research International. 2020;2020.
23. Netsi E, Coll CVN, Stein A, Silveira MF, Bertoldi AD, Bassani DG, et al. Female infants are more susceptible to the effects of maternal antenatal depression; findings from the Pelotas (Brazil) Birth Cohort Study. Journal of Affective Disorders. 2020;267:315-24.
24. Avilla JC, Giugliani C, Bizon A, Martins ACM, Senna AFK, Giugliani ERJ. Association between maternal satisfaction with breastfeeding and postpartum depression symptoms. PLoS ONE [Electronic Resource]. 2020;15(11):e0242333.
25. Leite TH, Pereira APE, Leal MdC, da Silva AAM. Disrespect and abuse towards women during childbirth and postpartum depression: Findings from Birth in Brazil Study. Journal of Affective Disorders. 2020;273:391-401.
26. Farias-Antunez S, Santos IS, Matijasevich A, de Barros AJD. Maternal mood symptoms in pregnancy and postpartum depression: association with exclusive breastfeeding in a population-based birth cohort. Social Psychiatry & Psychiatric Epidemiology. 2020;55(5):635-43.
27. Silveira MF, Mesenburg MA, Bertoldi AD, De Mola CL, Bassani DG, Domingues MR, et al. The association between disrespect and abuse of women during childbirth and postpartum depression: Findings from the 2015 Pelotas birth cohort study. Journal of Affective Disorders. 2019;256:441-7.
28. Silva RS, Junior RA, Sampaio VS, Rodrigues KO, Fronza M. Postpartum depression: a case-control study. Journal of Maternal Fetal and Neonatal Medicine. 2019.
29. Callo-Quinte G, Del-Ponte B, Ruivo ACO, Moreira LR, Konsgen BI, Wehrmeister FC, et al. Maternal depression symptoms and use of child health-care services at The Pelotas 2004 Birth Cohort. Journal of Affective Disorders. 2019;253:303-7.
30. Araujo IS, Aquino KS, Fagundes LKA, Santos VC. Postpartum Depression: Epidemiological Clinical Profile of Patients Attended In a Reference Public Maternity in Salvador-BA. Revista Brasileira de Ginecologia e Obstetricia. 2019;41(3):155-63.
31. Costa DO, Souza FIS, Pedroso GC, Strufaldi MWL. Mental disorders in pregnancy and newborn conditions: longitudinal study with pregnant women attended in primary care. Ciencia & Saude Coletiva. 2018;23(3):691-700.
32. Pinto TJ, Vilela AA, Farias DR, Lepsch J, Cunha GM, Vaz JS, et al. Serum n-3 polyunsaturated fatty acids are inversely associated with longitudinal changes in depressive symptoms during pregnancy. Epidemiology & Psychiatric Science. 2017;26(2):157-68.
33. Paskulin JTA, Drehmer M, Olinto MT, Hoffmann JF, Pinheiro AP, Schmidt MI, et al. Association between dietary patterns and mental disorders in pregnant women in Southern Brazil. Revista Brasileira de Psiquiatria. 2017;39(3):208-15.
34. Coll CdVN, da Silveira MF, Bassani DG, Netsi E, Wehrmeister FC, Barros FC, et al. Antenatal depressive symptoms among pregnant women: Evidence from a Southern Brazilian population-based cohort study. Journal of Affective Disorders. 2017:140-6.
35. Faisal-Cury A, Menezes PR, Quayle J, Matijasevich A. Unplanned pregnancy and risk of maternal depression: secondary data analysis from a prospective pregnancy cohort. Psychology Health & Medicine. 2017;22(1):65-74.
36. Moraes EV, Campos RN, Avelino MM. Depressive Symptoms in Pregnancy: The Influence of Social, Psychological and Obstetric Aspects. Revista Brasileira de Ginecologia e Obstetricia. 2016;38(6):293-300.
37. Correa H, Castro ECT, Santos W, Romano-Silva MA, Santos LM. Postpartum depression symptoms among Amazonian and Northeast Brazilian women. Journal of Affective Disorders. 2016;204:214-8.
38. Hassan BK, Werneck GL, Hasselmann MH. Maternal mental health and nutritional status of six-month-old infants. Revista De Saude Publica. 2016;50.
39. de Jesus Silva MM, Peres Rocha Carvalho Leite E, Alves Nogueira D, Clapis MJ. Depression in pregnancy. Prevalence and associated factors. Investigacion y educacion en enfermeria. 2016;34(2):342-50.
40. Castro ECT, Cardoso MN, Brancaglion MYM, Faria GC, Garcia FD, Nicolato R, et al. Antenatal depression: Prevalence and risk factor patterns across the gestational period. Journal of Affective Disorders. 2016;192:70-5.
41. de Figueiredo FP, Parada AP, Cardoso VC, Batista RFL, da Silva AAM, Barbieri MA, et al. Postpartum depression screening by telephone: a good alternative for public health and research. Archives of Womens Mental Health. 2015;18(3):547-53.

© 2023 Mitchell AR et al. *JAMA Psychiatry.*

42. Ferreira CR, Orsini MC, Vieira CR, Paffaro AMD, Silva RR. Prevalence of anxiety symptoms and depression in the third gestational trimester. Archives of Gynecology and Obstetrics. 2015;291(5):999-1003.
43. Vaz JS, Kac G, Nardi AE, Hibbeln JR. Omega-6 fatty acids and greater likelihood of suicide risk and major depression in early pregnancy. Journal of Affective Disorders. 2014;152-154(1):76-82.
44. Teofilo MM, Farias DR, Pinto Tde J, Vilela AA, Vaz Jdos S, Nardi AE, et al. HDL-cholesterol concentrations are inversely associated with Edinburgh Postnatal Depression Scale scores during pregnancy: results from a Brazilian cohort study. Journal of Psychiatric Research. 2014;58:181-8.
45. Coelho FMD, Pinheiro RT, Silva RA, Quevedo LD, Souza LDD, de Matos MB, et al. Parental bonding and suicidality in pregnant teenagers: a population-based study in southern Brazil. Social Psychiatry and Psychiatric Epidemiology. 2014;49(8):1241-8.
46. Zaconeta AM, De Queiroz IFB, Amato AA, Da Mottal LDC, Casulari LA. Depression with postpartum onset: A prospective cohort study in women undergoing elective cesarean section in Brasilia, Brazil. Revista Brasileira de Ginecologia e Obstetricia. 2013;35(3):130-5.
47. Takahasi EHM, e Alves MTSSB, Alves GS, da Silva AAM, Batista RFL, Simoes VMF, et al. Mental health and physical inactivity during pregnancy: A cross-sectional study nested in the BRISA cohort study. Cadernos de Saude Publica. 2013;29(8):1583-94.
48. Farias DR, Pinto Tde J, Teofilo MM, Vilela AA, Vaz Jdos S, Nardi AE, et al. Prevalence of psychiatric disorders in the first trimester of pregnancy and factors associated with current suicide risk. Psychiatry Research. 2013;210(3):962-8.
49. Faisal-Cury A, Menezes PR, d'Oliveira AF, Schraiber LB, Lopes CS. Temporal relationship between intimate partner violence and postpartum depression in a sample of low income women. Maternal and child health journal. 2013;17(7):1297-303.
50. da Silva RA, da Costa Ores L, Jansen K, da Silva Moraes IG, de Mattos Souza LD, Magalhaes P, et al. Suicidality and associated factors in pregnant women in Brazil. Community Mental Health Journal. 2012;48(3):392-5.
51. Tavares D, Quevedo L, Jansen K, Souza L, Pinheiro R, Silva R. Prevalence of suicide risk and comorbidities in postpartum women in Pelotas. Revista Brasileira de Psiquiatria. 2012;34(3):270-6.
52. Silva R, Jansen K, Souza L, Quevedo L, Barbosa L, Moraes I, et al. Sociodemographic risk factors of perinatal depression: a cohort study in the public health care system. Revista Brasileira de Psiquiatria. 2012;34(2):143-8.
53. Pinheiro RT, Coelho FMD, da Silva RA, Quevedo LD, Souza LDD, Castelli RD, et al. Suicidal behavior in pregnant teenagers in southern Brazil: Social, obstetric and psychiatric correlates. Journal of Affective Disorders. 2012;136(3):520-5.
54. Manzolli P, Nunes MA, Schmidt MI, Ferri CP. Abuse against women, depression, and infant morbidity: a primary care cohort study in Brazil. American Journal of Preventive Medicine. 2012;43(2):188-95.
55. Faisal-Cury A, Savoia MG, Menezes PR. Coping style and depressive symptomatology during pregnancy in a private setting sample. Spanish Journal of Psychology. 2012;15(1):295-305.
56. da Rocha CMM, Kac G. High dietary ratio of omega-6 to omega-3 polyunsaturated acids during pregnancy and prevalence of post-partum depression. Maternal and Child Nutrition. 2012;8(1):36-48.
57. Bottino MN, Nadanovsky P, Moraes CL, Reichenheim ME, Lobato G. Reappraising the relationship between maternal age and postpartum depression according to the evolutionary theory: Empirical evidence from a survey in primary health services. Journal of Affective Disorders. 2012;142(1-3):219-24.
58. Melo EF, Jr., Cecatti JG, Pacagnella RC, Leite DF, Vulcani DE, Makuch MY. The prevalence of perinatal depression and its associated factors in two different settings in Brazil. Journal of Affective Disorders. 2012;136(3):1204-8.
59. Lobato G, Moraes CL, Dias AS, Reichenheim ME. Alcohol misuse among partners: a potential effect modifier in the relationship between physical intimate partner violence and postpartum depression. Social Psychiatry and Psychiatric Epidemiology. 2012;47(3):427-38.
60. Faisal-Cury A, Menezes PR. Antenatal depression strongly predicts postnatal depression in primary health care. Revista Brasileira De Psiquiatria. 2012;34(4):446-50.
61. Pinheiro KA, Pinheiro RT, Silva RA, Coelho FM, Quevedo Lde A, Godoy RV, et al. Chronicity and severity of maternal postpartum depression and infant sleep disorders: a population-based cohort study in southern Brazil. Infant Behavior & Development. 2011;34(2):371-3.
62. Dias FMV, Claudio Jr SD, Franco GC, Teixeira AL, Ribeiro AM. Pregnancy is associated with psychiatric symptoms in a low-income countryside community of Brazil. Neuropsychiatric Disease and Treatment. 2011;7(1):709-14.

© 2023 Mitchell AR et al. *JAMA Psychiatry.*

63. Silva CS, Ronzani TM, Furtado EF, Aliane PP, Moreira-Almeida A. Relationship between religious practice, alcohol use, and psychiatric disorders among pregnant women. [Portuguese, English]. Revista de Psiquiatria Clinica. 2010;37(4):152-6.
64. Manzolli P, Nunes MA, Schmidt MI, Pinheiro AP, Soares RM, Giacomello A, et al. Violence and depressive symptoms during pregnancy: a primary care study in Brazil. Social Psychiatry & Psychiatric Epidemiology. 2010;45(10):983-8.
65. Ludermir AB, Lewis G, Valongueiro SA, De Araujo TVB, Araya R. Violence against women by their intimate partner during pregnancy and postnatal depression: A prospective cohort study. The Lancet. 2010;376(9744):903-10.
66. da Silva RA, Jansen K, Souza LDD, Moraes IGD, Tomasi E, da Silva GD, et al. Depression during pregnancy in the Brazilian public health care system. Revista Brasileira De Psiquiatria. 2010;32(2):139-44.
67. Jansen K, Curra AR, Souza LDDM, Pinheiro RT, Moraes IGDS, Da Cunha MS, et al. Tobacco smoking and depression during pregnancy. Revista de Psiquiatria do Rio Grande do Sul. 2010;32(2):44-7.
68. Cantilino A, Zambaldi CF, De Albuquerque TLC, Paes JA, Montenegro ACP, Sougey EB. Postpartum depression in Recife-Brazil: Prevalence and association with bio-socio-demographic factors. Jornal Brasileiro de Psiquiatria. 2010;59(1):1-9.
69. Zambaldi CF, Cantilino A, Montenegro AC, Paes JA, de Albuquerque TL, Sougey EB. Postpartum obsessive-compulsive disorder: prevalence and clinical characteristics. Comprehensive Psychiatry. 2009;50(6):503-9.
70. Pereira PK, Lovisi GM, Pilowsky DL, Lima LA, Legay LF. Depression during pregnancy: prevalence and risk factors among women attending a public health clinic in Rio de Janeiro, Brazil. Cadernos De Saude Publica. 2009;25(12):2725-36.
71. Matijasevich A, Golding J, Smith GD, Santos IS, Barros AJ, Victora CG. Differentials and income-related inequalities in maternal depression during the first two years after childbirth: birth cohort studies from Brazil and the UK. Clinical Practice & Epidemiology in Mental Health [Electronic Resource]: CP & EMH. 2009;5:12.
72. Tannous L, Gigante LP, Fuchs SC, Busnello ED. Postnatal depression in Southern Brazil: prevalence and its demographic and socioeconomic determinants. BMC Psychiatry. 2008;8:1.
73. Hasselmann MH, Werneck GL, Da Silva CVC. Symptoms of postpartum depression and early interruption of exclusive breastfeeding in the first two months of life. Cadernos de Saude Publica. 2008;24(SUPPL. 2):S341-S52.
74. Cantilino A, Carvalho JA, Maia A, Albuquerque C, Cantilino G, Sougey EB. Translation, validation and cultural aspects of postpartum depression screening scale in Brazilian Portuguese. Transcultural Psychiatry. 2007;44(4):672-84.
75. Faisal-Cury A, Rossi Menezes P. Prevalence of anxiety and depression during pregnancy in a private setting sample. Archives of Women's Mental Health. 2007;10(1):25-32.
76. Pinheiro RT, Magalhaes PVS, Horta BL, Pinheiro KAT, Da Silva RA, Pinto RH. Is paternal postpartum depression associated with maternal postpartum depression? Population-based study in Brazil. Acta Psychiatrica Scandinavica. 2006;113(3):230-2.
77. Faisal-Cury A, Tedesco JJ, Kahhale S, Menezes PR, Zugaib M. Postpartum depression: in relation to life events and patterns of coping. Archives of Women's Mental Health. 2004;7(2):123-31.
78. Da-Silva VA, Moraes-Santos AR, Carvalho MS, Martins ML, Teixeira NA. Prenatal and postnatal depression among low income Brazilian women. Brazilian Journal of Medical & Biological Research. 1998;31(6):799-804.
79. Zhang CJP, Wu H, He Z, Chan NK, Huang J, Wang H, et al. Psychobehavioral Responses, Post-Traumatic Stress and Depression in Pregnancy During the Early Phase of COVID-19 Outbreak. Psychiatric Research and Clinical Practice. 2021;3(1):46-54.
80. Yang X, Song B, Wu A, Mo PKH, Di J, Wang Q, et al. Social, Cognitive, and eHealth Mechanisms of COVID-19-Related Lockdown and Mandatory Quarantine That Potentially Affect the Mental Health of Pregnant Women in China: Cross-Sectional Survey Study. Journal of Medical Internet Research. 2021;23(1):e24495.
81. Xie M, Wang X, Zhang J, Wang Y. Alteration in the psychologic status and family environment of pregnant women before and during the COVID-19 pandemic. International Journal of Gynaecology & Obstetrics. 2021;153(1):71-5.
82. Wang Q, Mo PKH, Song B, Di JL, Zhou FR, Zhao J, et al. Mental health and preventive behaviour of pregnant women in China during the early phase of the COVID-19 period. Infect Dis Poverty. 2021;10(1):37.

© 2023 Mitchell AR et al. *JAMA Psychiatry.*

83. Shi X, Ying Y, Yu Z, Xing M, Zhu J, Feng W, et al. Risk factors for postpartum depression in Chinese women: A cross-sectional study at 6 weeks postpartum. J Psychosom Res. 2021;140:110295.
84. Peng S, Lai X, Du Y, Meng L, Gan Y, Zhang X. Prevalence and risk factors of postpartum depression in China: A hospital-based cross-sectional study. Journal of Affective Disorders. 2021;282:1096-100.
85. Lin W, Wu B, Chen B, Lai G, Huang S, Li S, et al. Sleep Conditions Associate with Anxiety and Depression Symptoms among Pregnant Women during the Epidemic of COVID-19 in Shenzhen. Journal of Affective Disorders. 2021;281:567-73.
86. Jiang H, Jin L, Qian X, Xiong X, La X, Chen W, et al. The mental health status and approaches of accessing antenatal care information among pregnant women during COVID-19 epidemic：a cross-sectional study in China. Journal of Medical Internet Research. 2021;23(1):N.PAG-N.PAG.
87. Dong HX, Hu RN, Lu C, Huang DJ, Cui DD, Huang GY, et al. Investigation on the mental health status of pregnant women in China during the Pandemic of COVID-19. Archives of Gynecology and Obstetrics. 2021;303(2):463-9.
88. Mo PKH FV, Song B, Di J, Wang Q, Wang L. Association of Perceived Threat, Negative Emotions, and Self-Efficacy With Mental Health and Personal Protective Behavior Among Chinese Pregnant Women During the COVID-19 Pandemic: Cross-sectional Survey Study. J Med Internet Res 2021;23(4).
89. Li C, Huo L, Wang R, Qi L, Wang W, Zhou X, et al. The prevalence and risk factors of depression in prenatal and postnatal women in China with the outbreak of Corona Virus Disease 2019. Journal of Affective Disorders. 2021;282:1203-9.
90. Huang Y, Liu Y, Wang Y, Liu D. Family function fully mediates the relationship between social support and perinatal depression in rural Southwest China. BMC Psychiatry. 2021;21(1):151.
91. Zhou YJ, Shi H, Liu ZK, Peng SX, Wang RX, Qi L, et al. The prevalence of psychiatric symptoms of pregnant and non-pregnant women during the COVID-19 epidemic. Translational Psychiatry. 2020;10(1).
92. Zhang LJ, Yang XS, Zhao JF, Zhang WY, Cui C, Yang FZ, et al. Prevalence of Prenatal Depression Among Pregnant Women and the Importance of Resilience: A Multi-Site Questionnaire-Based Survey in Mainland China. Frontiers in Psychiatry. 2020;11.
93. Zhang WN, Liu L, Cheng QJ, Chen Y, Xu D, Gong WJ. The Relationship Between Images Posted by New Mothers on WeChat Moments and Postpartum Depression: Cohort Study. Journal of Medical Internet Research. 2020;22(11).
94. Zhang X, Sun JW, Wang J, Chen QY, Cao DF, Wang JW, et al. Suicide ideation among pregnant women: The role of different experiences of childhood abuse. Journal of Affective Disorders. 2020;266:182-6.
95. Zeng X, Li W, Sun H, Luo X, Garg S, Liu T, et al. Mental Health Outcomes in Perinatal Women During the Remission Phase of COVID-19 in China. Frontiers in psychiatry Frontiers Research Foundation. 2020;11:571876.
96. Zheng B, Yu Y, Zhu X, Hu Z, Zhou W, Yin S, et al. Association between family functions and antenatal depression symptoms: a cross-sectional study among pregnant women in urban communities of Hengyang city, China. BMJ Open. 2020;10(8):e036557.
97. Yu M, Gong W, Taylor B, Cai Y, Xu DR. Coping Styles in Pregnancy, Their Demographic and Psychological Influences, and Their Association with Postpartum Depression: A Longitudinal Study of Women in China. International Journal of Environmental Research & Public Health [Electronic Resource]. 2020;17(10):22.
98. Xiong R, Deng A. Prevalence and associated factors of postpartum depression among immigrant women in Guangzhou, China. BMC Pregnancy & Childbirth. 2020;20(1):247.
99. Wu YT, Zhang C, Liu H, Duan CC, Li C, Fan JX, et al. Perinatal depressive and anxiety symptoms of pregnant women during the coronavirus disease 2019 outbreak in China. American journal of obstetrics and gynecology. 2020;223(2).
100. Sun G, Wang Q, Lin Y, Li R, Yang L, Liu X, et al. Perinatal Depression of Exposed Maternal Women in the COVID-19 Pandemic in Wuhan, China. Frontiers in psychiatry Frontiers Research Foundation. 2020;11:551812.
101. Yu YH, Zhu XD, Xu HL, Hu Z, Zhou WS, Zheng BH, et al. Prevalence of depression symptoms and its influencing factors among pregnant women in late pregnancy in urban areas of Hengyang City, Hunan Province, China: a cross-sectional study. Bmj Open. 2020;10(9).
102. Minglu L, Fang F, Guanxi L, Yuxiang Z, Chaoqiong D, Xueqin Z. Influencing factors and correlation of anxiety, psychological stress sources, and psychological capital among women pregnant with a second child in Guangdong and Shandong Province. Journal of Affective Disorders. 2020;264:115-22.

© 2023 Mitchell AR et al. *JAMA Psychiatry.*

103. Min WB, Nie W, Song SY, Wang N, Nie WQ, Peng LX, et al. Associations between Maternal and Infant Illness and the Risk of Postpartum Depression in Rural China: A Cross-Sectional Observational Study. International Journal of Environmental Research and Public Health. 2020;17(24).
104. Liu Y, Guo NF, Li TT, Zhuang W, Jiang H. Prevalence and Associated Factors of Postpartum Anxiety and Depression Symptoms Among Women in Shanghai, China. Journal of Affective Disorders. 2020;274:848-56.
105. Liang PQ, Wang YD, Shi S, Liu Y, Xiong RB. Prevalence and factors associated with postpartum depression during the COVID-19 pandemic among women in Guangzhou, China: a cross-sectional study. Bmc Psychiatry. 2020;20(1).
106. Li X, Gao R, Dai X, Liu H, Zhang J, Liu X, et al. The association between symptoms of depression during pregnancy and low birth weight: a prospective study. BMC Pregnancy & Childbirth. 2020;20(1):147.
107. Li Q, Yang S, Xie M, Wu X, Huang L, Ruan W, et al. Impact of some social and clinical factors on the development of postpartum depression in Chinese women. BMC Pregnancy & Childbirth. 2020;20(1):226.
108. Gong M, Zhang S, Xi C, Luo M, Wang T, Wang Y, et al. Comprehensive intervention during pregnancy based on short message service to prevent or alleviate depression in pregnant women: A quasi-experimental study. Early Interv Psychiatry. 2021;15(2):352-9.
109. Guo XJ, Chen J, Ren JH, Deng X, Xu LZ. Comparisons on perinatal depression between the first-child women and the second-child women in West China under the universal 2-child policy: A STROBE compliant prospective cohort study. Medicine. 2020;99(23):e20641.
110. Bo HX, Yang Y, Chen J, Zhang M, Zhang DY, Li Y, et al. Prevalence of depressive symptoms among Chinese pregnant and postpartum women during the COVID-19 pandemic. Psychosomatic medicine Publish Ahead of Print. 2020;15.
111. Lu L, Duan Z, Wang Y, Wilson A, Yang Y, Zhu L, et al. Mental health outcomes among Chinese prenatal and postpartum women after the implementation of universal two-child policy. Journal of Affective Disorders. 2020;264:187-92.
112. Tang X, Lu Z, Hu D, Zhong X. Influencing factors for prenatal Stress, anxiety and depression in early pregnancy among women in Chongqing, China. Journal of Affective Disorders. 2019;253:292-302.
113. Sha T, Gao X, Chen C, Li L, Cheng G, Wu X, et al. A prospective study of maternal postnatal depressive symptoms with infant-feeding practices in a Chinese birth cohort. BMC Pregnancy & Childbirth. 2019;19(1):388.
114. Mak JKL, Lee AH, Pham NM, Tang L, Pan XF, Xu ZP, et al. Physical activity during early pregnancy and antenatal depression: A prospective cohort study. Mental Health and Physical Activity. 2019;16:54-9.
115. Ma X, Wang Y, Hu H, Tao XG, Zhang Y, Shi H. The impact of resilience on prenatal anxiety and depression among pregnant women in Shanghai. Journal of Affective Disorders. 2019;250:57-64.
116. Gao M, Hu J, Yang L, Ding N, Wei X, Li L, et al. Association of sleep quality during pregnancy with stress and depression: a prospective birth cohort study in China. BMC Pregnancy & Childbirth. 2019;19(1):444.
117. Hu Y, Wang Y, Wen S, Guo XJ, Xu LZ, Chen BH, et al. Association between social and family support and antenatal depression: a hospital-based study in Chengdu, China. Bmc Pregnancy and Childbirth. 2019;19(1).
118. Duan ZZ, Wang YY, Tao Y, Joanne LB, Yu RJ, Wang SL, et al. Relationship between trait neuroticism and suicidal ideation among postpartum women in China: Testing a mediation model. Journal of Affective Disorders. 2019;256:532-5.
119. Ding G, Niu L, Vinturache A, Zhang J, Lu M, Gao Y, et al. "Doing the month" and postpartum depression among Chinese women: A Shanghai prospective cohort study. Women & Birth: Journal of the Australian College of Midwives. 2020;33(2):e151-e8.
120. Zhou C, Zheng W, Yuan Q, Zhang B, Chen H, Wang W, et al. Associations between social capital and maternal depression: results from a follow-up study in China. BMC Pregnancy & Childbirth. 2018;18(1):45.
121. Zhang Y, Muyiduli X, Wang S, Jiang W, Wu J, Li M, et al. Prevalence and relevant factors of anxiety and depression among pregnant women in a cohort study from south-east China. Journal of Reproductive and Infant Psychology. 2018.
122. Zheng XJ, Morrell J, Watts K. Changes in maternal self-efficacy, postnatal depression symptoms and social support among Chinese primiparous women during the initial postpartum period: A longitudinal study. Midwifery. 2018;62:151-60.

© 2023 Mitchell AR et al. *JAMA Psychiatry.*

123. Yu HH, Jiang XY, Bao W, Xu GF, Yang R, Shen M. Association of intimate partner violence during pregnancy, prenatal depression, and adverse birth outcomes in Wuhan, China. Bmc Pregnancy and Childbirth. 2018;18.
124. Xiong RB, Deng AW, Wan B, Liu Y. Prevalence and factors associated with postpartum depression in women from single-child families. International Journal of Gynecology & Obstetrics. 2018;141(2):194-9.
125. Song CH, Leng JH, Wang LS, Li W, Zhang S, Wang W, et al. ABO blood types and postpartum depression among Chinese women: A prospective cohort study in Tianjin, China. Women & Health. 2018;58(6):685-98.
126. Shi P, Ren H, Li H, Dai Q. Maternal depression and suicide at immediate prenatal and early postpartum periods and psychosocial risk factors. Psychiatry Research. 2018;261:298-306.
127. Chen L, Ding L, Qi M, Jiang C, Mao XM, Cai WZ. Incidence of and social-demographic and obstetric factors associated with postpartum depression: differences among ethnic Han and Kazak women of Northwestern China. Peerj. 2018;6.
128. Zhou C, Ogihara A, Chen H, Wang WJ, Huang L, Zhang BD, et al. Social capital and antenatal depression among Chinese primiparas: A cross-sectional survey. Psychiatry Research. 2017;257:533-9.
129. Zeng YC, Li YT, Xia HA, Wang SL, Zhou JX, Chen DJ. Retinoids, anxiety and peripartum depressive symptoms among Chinese women: a prospective cohort study. Bmc Psychiatry. 2017;17.
130. Wang YY, Li H, Wang YJ, Wang H, Zhang YR, Gong L, et al. Living with parents or with parents-in-law and postpartum depression: A preliminary investigation in China. Journal of Affective Disorders. 2017;218:335-8.
131. Yu Y, Li M, Pu L, Wang S, Wu J, Ruan L, et al. Sleep was associated with depression and anxiety status during pregnancy: a prospective longitudinal study. Archives of Women's Mental Health. 2017;20(5):695-701.
132. Liu S, Yan Y, Gao X, Xiang S, Sha T, Zeng G, et al. Risk factors for postpartum depression among Chinese women: path model analysis. BMC Pregnancy & Childbirth. 2017;17(1):133.
133. Li Y, Long ZT, Cao DF, Cao FL. Maternal history of child maltreatment and maternal depression risk in the perinatal period: A longitudinal study. Child Abuse & Neglect. 2017;63:192-201.
134. Li Y, Long Z, Cao D, Cao F. Social support and depression across the perinatal period: A longitudinal study. Journal of Clinical Nursing. 2017;26(17-18):2776-83.
135. Huang JY, Wen GM, Yang WK, Yao ZJ, Wu CA, Ye XH. The association between second-hand smoke exposure and depressive symptoms among pregnant women. Psychiatry Research. 2017;256:469-74.
136. Wang Y, Wang XH, Liu FN, Jiang XN, Xiao Y, Dong XH, et al. Negative Life Events and Antenatal Depression among Pregnant Women in Rural China: The Role of Negative Automatic Thoughts. Plos One. 2016;11(12).
137. Li Y, Zeng Y, Zhu W, Cui Y, Li J. Path model of antenatal stress and depressive symptoms among Chinese primipara in late pregnancy. BMC Pregnancy & Childbirth. 2016;16(1):180.
138. Zeng Y, Cui Y, Li J. Prevalence and predictors of antenatal depressive symptoms among Chinese women in their third trimester: a cross-sectional survey. BMC Psychiatry. 2015;15:66.
139. Ren JH, Jiang XL, Yao JR, Li XR, Liu XH, Pang M, et al. Depression, Social Support, and Coping Styles among Pregnant Women after the Lushan Earthquake in Ya'an, China. Plos One. 2015;10(8).
140. Fu CW, Liu JT, Tu WJ, Yang JQ, Cao Y. Association between serum 25-hydroxyvitamin D levels measured 24 hours after delivery and postpartum depression. BJOG: An International Journal of Obstetrics & Gynaecology. 2015;122(12):1688-94.
141. Deng AW, Xiong RB, Jiang TT, Luo YP, Chen WZ. Prevalence and risk factors of postpartum depression in a population-based sample of women in Tangxia Community, Guangzhou. Asian Pacific Journal of Tropical Medicine. 2014;7(3):244-9.
142. Dong XH, Qu ZY, Liu FN, Jiang X, Wang Y, Chui CHK, et al. Depression and its risk factors among pregnant women in 2008 Sichuan earthquake area and non-earthquake struck area in China. Journal of Affective Disorders. 2013;151(2):566-72.
143. Lau Y. Risk factors associated with antenatal depressive symptomatology among Chengdu Chinese women. Alternative medicine yearbook, 2011. Hauppauge, NY: Nova Biomedical Books; US; 2013. p. 107-21.
144. Zhang Y, Zou SH, Cao YP, Zhang YL. Relationship between domestic violence and postnatal depression among pregnant Chinese women. International Journal of Gynecology & Obstetrics. 2012;116(1):26-30.
145. Qu ZY, Wang XH, Tian DH, Zhao Y, Zhang Q, He H, et al. Posttraumatic stress disorder and depression among new mothers at 8 months later of the 2008 Sichuan earthquake in China. Archives of Womens Mental Health. 2012;15(1):49-55.

© 2023 Mitchell AR et al. *JAMA Psychiatry.*

146. Qu ZY, Tian DH, Zhang Q, Wang XH, He H, Zhang XL, et al. The impact of the catastrophic earthquake in China's Sichuan province on the mental health of pregnant women. Journal of Affective Disorders. 2012;136(1-2):117-23.
147. Li J, Mao J, Du YK, Morris JL, Gong GL, Xiong XJ. Health-Related Quality of Life Among Pregnant Women With and Without Depression in Hubei, China. Maternal and Child Health Journal. 2012;16(7):1355-63.
148. Mao Q, Zhu LX, Su XY. A comparison of postnatal depression and related factors between Chinese new mothers and fathers. Journal of Clinical Nursing. 2011;20(5-6):645-52.
149. Lau Y, Yin L, Wang YQ. Severe Antenatal Depressive Symptoms Before and After the 2008 Wenchuan Earthquake in Chengdu, China. Jognn-Journal of Obstetric Gynecologic and Neonatal Nursing. 2011;40(1):62-74.
150. Xie RH, Yang J, Liao S, Xie H, Walker M, Wen SW. Prenatal family support, postnatal family support and postpartum depression. Australian & New Zealand Journal of Obstetrics & Gynaecology. 2010;50(4):340-5.
151. Lau Y, Wang Y, Yin L, Chan KS, Guo X. Validation of the Mainland Chinese version of the Edinburgh Postnatal Depression Scale in Chengdu mothers. International Journal of Nursing Studies. 2010;47(9):1139-51.
152. Xie RH, He GP, Koszycki D, Walker M, Wen SW. Fetal Sex, Social Support, and Postpartum Depression. Canadian Journal of Psychiatry-Revue Canadienne De Psychiatrie. 2009;54(11):750-6.
153. Wan EY, Moyer CA, Harlow SD, Fan ZT, Jie Y, Yang HX. Postpartum depression and traditional postpartum care in China: Role of Zuoyuezi. International Journal of Gynecology & Obstetrics. 2009;104(3):209-13.
154. Qiao YX, Wang J, Li J, Ablat A. The prevalence and related risk factors of anxiety and depression symptoms among Chinese pregnant women in Shanghai. Australian & New Zealand Journal of Obstetrics & Gynaecology. 2009;49(2):185-90.
155. Gao LL, Chan SWC, Mao Q. Depression, Perceived Stress, and Social Support Among First-Time Chinese Mothers and Fathers in the Postpartum Period. Research in Nursing & Health. 2009;32(1):50-8.
156. Xie RH, He G, Liu A, Bradwejn J, Walker M, Wen SW. Fetal gender and postpartum depression in a cohort of Chinese women. Social Science & Medicine. 2007;65(4):680-4.
157. Wang SY, Jiang XY, Jan WC, Chen CH. A comparative study of postnatal depression and its predictors in Taiwan and mainland China. American Journal of Obstetrics & Gynecology. 2003;189(5):1407-12.
158. Yotebieng KA, Fokong K, Yotebieng M. Depression, retention in care, and uptake of PMTCT service in Kinshasa, the Democratic Republic of Congo: a prospective cohort. AIDS Care. 2017;29(3):285-9.
159. Goweda R, Metwally T. Prevalence and associated risk factors of postpartum depression: A cross sectional study. Revista de Psiquiatria Clinica. 2020;47(4):106-9.
160. Wassif OM, Abdo AS, Elawady MA, Abd Elmaksoud AE, Eldesouky RS. Assessment of Postpartum Depression and Anxiety among Females Attending Primary Health Care Facilities in Qaliubeya Governorate, Egypt. Journal of Environmental & Public Health. 2019:1-10.
161. Abdelhai R, Mosleh H. Screening for antepartum anxiety and depression and their association with domestic violence among Egyptian pregnant women. Journal of the Egyptian Public Health Association. 2015;90(3):101-8.
162. Mohamed HA, Spencer SL, Al Swasy AH, Swidan SE, Abouelenien MS. A social and Biological Approach for Postpartum Depression in Egypt. Woman - Psychosomatic Gynaecology and Obstetrics. 2014;1(C):30-9.
163. Gebregziabher NK, Netsereab TB, Fessaha YG, Alaza FA, Ghebrehiwet NK, Sium AH. Prevalence and associated factors of postpartum depression among postpartum mothers in central region, Eritrea: a health facility based survey. Bmc Public Health. 2020;20(1).
164. Dlamini LP, Mahanya S, Dlamini SD, Shongwe MC. Prevalence and factors associated with postpartum depression at a primary healthcare facility in Eswatini. South African Journal of Psychiatry. 2019;25 (no pagination).
165. Malqvist M, Clarke K, Matsebula T, Bergman M, Tomlinson M. Screening for Antepartum Depression Through Community Health Outreach in Swaziland. Journal of Community Health. 2016;41(5):946-52.
166. Zewdu LB, Reta MM, Yigzaw N, Tamirat KS. Prevalence of suicidal ideation and associated factors among HIV positive perinatal women on follow-up at Gondar town health institutions, Northwest Ethiopia: a cross-sectional study. BMC Pregnancy & Childbirth. 2021;21(1):42.
167. Zelalem ED, Asaye MM, Mihret MS. Antenatal depression and its correlates on northwestern Ethiopian women: community-based cross-sectional study. Pan African Medical Journal. 2020;36.

© 2023 Mitchell AR et al. *JAMA Psychiatry.*

168. Tesfaye Y, Agenagnew L. Antenatal Depression and Associated Factors among Pregnant Women Attending Antenatal Care Service in Kochi Health Center, Jimma Town, Ethiopia. Journal of Pregnancy. 2021;2021.
169. Dadi AF, Miller ER, Woodman RJ, Azale T, Mwanri L. Effect of antenatal depression on adverse birth outcomes in Gondar town, Ethiopia: A community-based cohort study. PLoS ONE. 2020;15(6).
170. Bante A, Mersha A, Zerdo Z, Wassihun B, Yeheyis T. Comorbid anxiety and depression: Prevalence and associated factors among pregnant women in Arba Minch zuria district, Gamo zone, southern Ethiopia. PLoS ONE [Electronic Resource]. 2021;16(3):e0248331.
171. Ashenafi W, Mengistie B, Egata G, Berhane Y. The role of intimate partner violence victimization during pregnancy on maternal postpartum depression in Eastern Ethiopia. Sage Open Medicine. 2021;9.
172. Wubetu AD, Engidaw NA, Gizachew KD. Prevalence of postpartum depression and associated factors among postnatal care attendees in Debre Berhan, Ethiopia, 2018. BMC Pregnancy & Childbirth. 2020;20(1):189.
173. Tiki T, Taye K, Duko B. Prevalence and factors associated with depression among pregnant mothers in the West Shoa zone, Ethiopia: a community-based cross-sectional study. Annals of General Psychiatry. 2020;19:24.
174. Necho M, Belete A, Zenebe Y. The association of intimate partner violence with postpartum depression in women during their first month period of giving delivery in health centers at Dessie town, 2019. Annals of General Psychiatry. 2020;19:59.
175. Dadi AF, Miller ER, Woodman R, Bisetegn TA, Mwanri L. Antenatal depression and its potential causal mechanisms among pregnant mothers in Gondar town: application of structural equation model. BMC Pregnancy & Childbirth. 2020;20(1):168.
176. Chuma BT, Sagaro GG, Astawesegn FH. Magnitude and Predictors of Antenatal Depression among Pregnant Women Attending Antenatal Care in Sodo Town, Southern Ethiopia: Facility-Based Cross-Sectional Study. Depression Research and Treatment. 2020;2020:6718342.
177. Ayen SS, Alemayehu S, Tamene F. Antepartum Depression and Associated Factors Among Pregnant Women Attending ANC Clinics in Gurage Zone Public Health Institutions, SNNPR, Ethiopia, 2019. Psychology Research and Behavior Management. 2020;13:1365-72.
178. Anato A, Baye K, Tafese Z, Stoecker BJ. Maternal depression is associated with child undernutrition: A cross-sectional study in Ethiopia. Maternal & Child Nutrition. 2020;16(3):1-9.
179. Lodebo M, Birhanu D, Abdu S, Yohannes T. Magnitude of Antenatal Depression and Associated Factors among Pregnant Women in West Badewacho Woreda, Hadiyya Zone, South Ethiopia: Community Based Cross Sectional Study. Depression Research and Treatment. 2020;2020:2950536.
180. Dadi AF, Mwanri L, Woodman RJ, Azale T, Miller ER. Causal mechanisms of postnatal depression among women in Gondar town, Ethiopia: application of a stress-process model with generalized structural equation modeling. Reproductive Health. 2020;17(1):63.
181. Shitu S, Geda B, Dheresa M. Postpartum depression and associated factors among mothers who gave birth in the last twelve months in Ankesha district, Awi zone, North West Ethiopia. Bmc Pregnancy and Childbirth. 2019;19(1).
182. Habtamu Belete A, Alemayehu Assega M, Alemu Abajobir A, Abebe Belay Y, Kassahun Tariku M. Prevalence of antenatal depression and associated factors among pregnant women in Aneded woreda, North West Ethiopia: a community based cross-sectional study. BMC Research Notes. 2019;12(1):713.
183. Bitew T, Hanlon C, Medhin G, Fekadu A. Antenatal predictors of incident and persistent postnatal depressive symptoms in rural Ethiopia: a population-based prospective study. Reproductive Health. 2019;16.
184. Belay S, Astatkie A, Emmelin M, Hinderaker SG. Intimate partner violence and maternal depression during pregnancy: A community-based cross-sectional study in Ethiopia. PLoS ONE [Electronic Resource]. 2019;14(7):e0220003.
185. Abebe A, Tesfaw G, Mulat H, Hibdye G, Yohannes k. Postpartum depression and associated factors among mothers in Bahir Dar Town, Northwest Ethiopia. Annals of General Psychiatry. 2019;18(1):N.PAG-N.PAG.
186. Abadiga M. Magnitude and associated factors of postpartum depression among women in Nekemte town, East Wollega zone, west Ethiopia, 2019: A community-based study. PLoS ONE [Electronic Resource]. 2019;14(11):e0224792.
187. Duko B, Ayano G, Bedaso A. Depression among pregnant women and associated factors in Hawassa city, Ethiopia: An institution-based cross-sectional study. Reproductive Health. 2019;16(1).
188. Woldetensay YK, Belachew T, Biesalski HK, Ghosh S, Lacruz ME, Scherbaum V, et al. The role of nutrition, intimate partner violence and social support in prenatal depressive symptoms in rural

Ethiopia: Community based birth cohort study 11 Medical and Health Sciences 1117 Public Health and Health Services. BMC Pregnancy and Childbirth. 2018;18(1).
189. Toru T, Chemir F, Anand S. Magnitude of postpartum depression and associated factors among women in Mizan Aman town, Bench Maji zone, Southwest Ethiopia 11 Medical and Health Sciences 1117 Public Health and Health Services. BMC Pregnancy and Childbirth. 2018;18(1).
190. Kerie S, Menberu M, Niguse W. Prevalence and associated factors of postpartum depression in Southwest, Ethiopia, 2017: a cross-sectional study. BMC Research Notes. 2018;11(1):623.
191. Azale T, Fekadu A, Medhin G, Hanlon C. Coping strategies of women with postpartum depression symptoms in rural Ethiopia: A cross-sectional community study. BMC Psychiatry. 2018;18(1).
192. Adamu AF, Adinew YM. Domestic Violence as a Risk Factor for Postpartum Depression Among Ethiopian Women: Facility Based Study. Clinical Practice & Epidemiology in Mental Health [Electronic Resource]: CP & EMH. 2018;14:109-19.
193. Fantahun A, Cherie A, Deribe L. Prevalence and Factors Associated with Postpartum Depression Among Mothers Attending Public Health Centers of Addis Ababa, Ethiopia, 2016. Clinical Practice & Epidemiology in Mental Health [Electronic Resource]: CP & EMH. 2018;14:196-206.
194. Belay YA, Moges NA, Hiksa FF, Arado KK, Liben ML. Prevalence of Antenatal Depression and Associated Factors among Pregnant Women Attending Antenatal Care at Dubti Hospital: A Case of Pastoralist Region in Northeast Ethiopia. Depression Research and Treatment. 2018;2018:1659089.
195. Mossie TB, Sibhatu AK, Dargie A, Ayele AD. Prevalence of Antenatal Depressive Symptoms and Associated Factors among Pregnant Women in Maichew, North Ethiopia: An Institution Based Study. Ethiopian Journal of Health Sciences. 2017;27(1):59-66.
196. Bitew T, Hanlon C, Kebede E, Honikman S, Onah MN, Fekadu A. Antenatal depressive symptoms and utilisation of delivery and postnatal care: a prospective study in rural Ethiopia. Bmc Pregnancy and Childbirth. 2017;17.
197. Bisetegn TA, Mihretie G, Muche T. Prevalence and Predictors of Depression among Pregnant Women in Debretabor Town, Northwest Ethiopia. PLoS ONE [Electronic Resource]. 2016;11(9):e0161108.
198. Ayele TA, Azale T, Alemu K, Abdissa Z, Mulat H, Fekadu A. Prevalence and Associated Factors of Antenatal Depression among Women Attending Antenatal Care Service at Gondar University Hospital, Northwest Ethiopia. PLoS ONE [Electronic Resource]. 2016;11(5):e0155125.
199. Biratu A, Haile D. Prevalence of antenatal depression and associated factors among pregnant women in Addis Ababa, Ethiopia: A cross-sectional study. Reproductive Health. 2015;12(1).
200. Sefogah PE, Samba A, Mumuni K, Kudzi W. Prevalence and key predictors of perinatal depression among postpartum women in Ghana. International Journal of Gynecology & Obstetrics. 2020;149(2):203-10.
201. Lillie M, Gallis JA, Hembling J, Owusu RK, Ali M, Abubakr-Bibilazu S, et al. Prevalence and Correlates of Depression Among Pregnant Women Enrolled in a Maternal and Newborn Health Program in Rural Northern Ghana: a Cross-sectional Survey. Global Social Welfare. 2020;7(2):131-40.
202. Anokye R, Acheampong E, Budu-Ainooson A, Obeng EI, Akwasi AG. Prevalence of postpartum depression and interventions utilized for its management. Annals of General Psychiatry. 2018;17:18.
203. Weobong B, Ten Asbroek AH, Soremekun S, Danso S, Owusu-Agyei S, Prince M, et al. Determinants of postnatal depression in rural ghana: findings from the don population based cohort study. Depression & Anxiety. 2015;32(2):108-19.
204. Weobong B, Ten Asbroek AHA, Soremekun S, Gram L, Amenga-Etego SD, Danso S, et al. Association between probable postnatal depression and increased infant mortality and morbidity: Findings from the DON population-based cohort study in rural Ghana. BMJ Open. 2015;5(8).
205. Weobong B, Ten Asbroek AHA, Soremekun S, Manu AA, Owusu-Agyei S, Prince M, et al. Association of antenatal depression with adverse consequences for the mother and newborn in rural Ghana: Findings from the DON population-based cohort study. PLoS ONE. 2014;9(12).
206. Weobong B, Soremekun S, ten Asbroek AHA, Amenga-Etego S, Danso S, Owusu-Agyei S, et al. Prevalence and determinants of antenatal depression among pregnant women in a predominantly rural population in Ghana: The DON population-based study. Journal of Affective Disorders. 2014;165:1-7.
207. Raghavan V, Khan HA, Seshu U, Rai SP, Durairaj J, Aarthi G, et al. Prevalence and risk factors of perinatal depression among women in rural Bihar: A community-based cross-sectional study. Asian Journal of Psychiatry. 2021;56.
208. Neelakanthi A, Venkatesh, S., Babu,, B., Nachiketha, S., & Gopalakrishnan, U. Prevalence and Risk Factors of Depressive Symptoms. Perinatology. 2021;21.
209. Lanjewar S, Nimkar S, Jungari S. Depressed Motherhood: Prevalence and Covariates of Maternal Postpartum Depression among Urban Mothers in India. Asian Journal of Psychiatry. 2021;57:102567.

210. Basu S, Budh N, Garg S, Singh MM, Sharma A. Postpartum depression burden and associated factors in mothers of infants at an urban primary health center in Delhi, India. Tzu Chi Medical Journal. 2021;33(1):70-3.
211. Ana Y, Lewis MG, van Schayck OCP, Babu GR. Is physical activity in pregnancy associated with prenatal and postnatal depressive symptoms?: Results from MAASTHI cohort study in South India. Journal of Psychosomatic Research. 2021;144:110390.
212. Badiya PK, Siddabattuni S, Dey D, Javvaji SK, Nayak SP, Hiremath AC, et al. Identification of clinical and psychosocial characteristics associated with perinatal depression in the south Indian population. General Hospital Psychiatry. 2020;66:161-70.
213. Murry LL, Devi YS, Joshi P, Dabas S, Kumari V, Singh KJ. Postpartum Depression and its Risk Factors among Indian Women. Nursing Journal of India. 2020;111(4):186-90.
214. Dahiya N, Aggarwal K, Kumar R. Prevalence and correlates of antenatal depression among women registered at antenatal clinic in North India. Tzu Chi Medical Journal. 2020;32(3):267-71.
215. Amipara T, Baria H, Nayak S. A study on postpartum depression and its association with infant feeding practices and infant nutritional status among mothers attending the anganwadi centers of Valsad district, Gujarat, India. Indian Journal of Community Medicine. 2020;45(3):299-302.
216. Khatri S, Murthy AK, Hashim U, Kuruthukulangara S, Kumari A, Lele PR. Psychological status of pregnant women during COVID-19 pandemic: A cross-sectional study from Mumbai. Journal of Marine Medical Society. 2020;22(3):113-7.
217. Goyal S, Gupta B, Sharma E, Dalal PK, Pradeep Y. Psychiatric Morbidity, Cultural Factors, and Health-Seeking Behaviour in Perinatal Women: A Cross-Sectional Study from a Tertiary Care Centre of North India. Indian Journal of Psychological Medicine. 2020;42(1):52-60.
218. Kantipudi SJ, Kannan GK, Viswanathan S, Ranganathan S, Menon J, Ramanathan S. Antenatal Depression and Generalized Anxiety Disorder in a Tertiary Hospital in South India. Indian Journal of Psychological Medicine. 2020;42(6):513-8.
219. Sheeba B, Nath A, Metgud CS, Krishna M, Venkatesh S, Vindhya J, et al. Prenatal Depression and Its Associated Risk Factors Among Pregnant Women in Bangalore: A Hospital Based Prevalence Study. Frontiers in Public Health. 2019;7.
220. Agarwala A, Rao PA, Narayanan P. Prevalence and predictors of postpartum depression among mothers in the rural areas of Udupi Taluk, Karnataka, India: A cross-sectional study. Clinical Epidemiology and Global Health. 2019;7(3):342-5.
221. Joshi MN, Raut AV. Maternal depression and its association with responsive feeding and nutritional status of infants: A cross-sectional study from a rural medical college in central India. Journal of Postgraduate Medicine. 2019;65(4):212-8.
222. JayaSalengia B, Rajeswari S, Nalini S. The Relationship between Maternal Confidence, Infant Temperament, and Postpartum Depression. Iranian Journal of Nursing and Midwifery Research. 2019;24(6):437-43.
223. Fuhr DC, Weobong B, Lazarus A, Vanobberghen F, Weiss HA, Singla DR, et al. Delivering the Thinking Healthy Programme for perinatal depression through peers: an individually randomised controlled trial in India. Lancet Psychiatry. 2019;6(2):115-27.
224. Kale DP, Tambawala ZY, Rajput NM. Postpartum depression prevalence in a tertiary care hospital in Mumbai, Maharashtra, India. Journal of SAFOG. 2019;11(4):239-42.
225. Jha P, Larsson M, Christensson K, Svanberg AS. Fear of childbirth and depressive symptoms among postnatal women: A cross-sectional survey from Chhattisgarh, India. Women and Birth. 2018;31(2):E122-E33.
226. Rathod SD, Honikman S, Hanlon C, Shidhaye R. Characteristics of perinatal depression in rural central, India: A cross-sectional study 11 Medical and Health Sciences 1117 Public Health and Health Services. International Journal of Mental Health Systems. 2018;12(1).
227. Gonzalez-Mesa E, Kabukcuoglu K, Korukcu O, Blasco M, Ibrahim N, Kavas T. Cultural factors influencing antenatal depression: A cross-sectional study in a cohort of Turkish and Spanish women at the beginning of the pregnancy. Journal of Affective Disorders. 2018;238:256-60.
228. Kishore MT, Satyanarayana V, Ananthanpillai ST, Desai G, Bhaskarapillai B, Thippeswamy H, et al. Life events and depressive symptoms among pregnant women in India: Moderating role of resilience and social support. International Journal of Social Psychiatry. 2018;64(6):570-7.
229. Zaidi F, Nigam A, Anjum R, Agarwalla R. Postpartum Depression in Women: A Risk Factor Analysis. Journal of Clinical and Diagnostic Research. 2017;11(8):QC13-QC6.
230. Shidhaye P, Shidhaye R, Phalke V. Association of gender disadvantage factors and gender preference with antenatal depression in women: a cross-sectional study from rural Maharashtra. Social Psychiatry and Psychiatric Epidemiology. 2017;52(6):737-48.

© 2023 Mitchell AR et al. *JAMA Psychiatry.*

231. Goyal K, Purbiya P, Lal SN, Kaur J, Anthwal P, Puliyel JM. Correlation of Infant Gender with Postpartum Maternal and Paternal Depression and Exclusive Breastfeeding Rates. Breastfeeding Medicine. 2017;12(5):279-82.
232. Boggaram SA, Singh H, Manikanta TS, Maheswari E. An exploratory study of identification of psychiatric disorders during pregnancy. Minerva Psichiatrica. 2017;58(4):203-8.
233. George C, Lalitha ARN, Antony A, Kumar AV, Jacob KS. Antenatal depression in coastal South India: Prevalence and risk factors in the community. International Journal of Social Psychiatry. 2016;62(2):141-7.
234. Sheela CN, Venkatesh S. Screening for Postnatal Depression in a Tertiary Care Hospital. Journal of Obstetrics and Gynecology of India. 2016;66:72-6.
235. Lakshmi Bhuvana G, Sripada R, Devi Priya S, Raja Sanjay BBP, Satya Rajeswari K, Rama Chandran S. Prevalence of postpartum depression at an Indian tertiary care teaching hospital. International Journal of Pharmaceutical and Clinical Research. 2016;8(6):616-8.
236. Srinivasan N, Murthy S, Singh AK, Upadhyay V, Mohan SK, Joshi A. Assessment of burden of depression during pregnancy among pregnant women residing in rural setting of chennai. Journal of Clinical and Diagnostic Research JCDR. 2015;9(4):LC08-12.
237. Shivalli S, Gururaj N. Postnatal depression among rural women in South India: do socio-demographic, obstetric and pregnancy outcome have a role to play? PLoS ONE [Electronic Resource]. 2015;10(4):e0122079.
238. Patel HL, Ganjiwale JD, Nimbalkar AS, Vani SN, Vasa R, Nimbalkar SM. Characteristics of Postpartum Depression in Anand District, Gujarat, India. Journal of Tropical Pediatrics. 2015;61(5):364-9.
239. Johnson AR, Edwin S, Joachim N, Mathew G, Ajay S, Joseph B. Postnatal depression among women availing maternal health services in a rural hospital in South India. Pakistan Journal of Medical Sciences. 2015;31(2):408-13.
240. Bodhare TN, Sethi P, Bele SD, Gayatri D, Vivekanand A. Postnatal quality of life, depressive symptoms, and social support among women in southern India. Women & Health. 2015;55(3):353-65.
241. Gupta S, Kishore J, Mala YM, Ramji S, Aggarwal R. Postpartum depression in North Indian women: Prevalence and risk factors. Journal of Obstetrics and Gynecology of India. 2013;63(4):223-9.
242. Dubey C, Gupta N, Bhasin S, Muthal RA, Arora R. Prevalence and associated risk factors for postpartum depression in women attending a tertiary hospital, Delhi, India. International Journal of Social Psychiatry. 2012;58(6):577-80.
243. Ghosh A, Goswami S. Evaluation of post partum depression in a tertiary hospital. Journal of Obstetrics and Gynecology of India. 2011;61(5):528-30.
244. Savarimuthu RJS, Ezhilarasu P, Charles H, Antonisamy B, Kurian S, Jacob KS. Post-partum depression in the community: a qualitative study from rural South India. International Journal of Social Psychiatry. 2010;56(1):94-102.
245. Mariam KA, Srinivasan K. Antenatal psychological distress and postnatal depression: A prospective study from an urban clinic. Asian Journal of Psychiatry. 2009;2(2):71-3.
246. Nagpal J, Dhar RSG, Sinha S, Bhargava V, Sachdeva A, Bhartia A. An exploratory study to evaluate the utility of an adapted Mother Generated Index (MGI) in assessment of postpartum quality of life in India. Health and Quality of Life Outcomes. 2008;6.
247. Patel V, DeSouza N, Rodrigues M, Patel V, DeSouza N, Rodrigues M. Postnatal depression and infant growth and development in low income countries: a cohort study from Goa, India. Archives of Disease in Childhood. 2003;88(1):34-7.
248. Patel V, Rodrigues M, DeSouza N. Gender, poverty, and postnatal depression: a study of mothers in Goa, India. American Journal of Psychiatry. 2002;159(1):43-7.
249. Chandran M, Tharyan P, Muliyil J, Abraham S. Post-partum depression in a cohort of women from a rural area of Tamil Nadu, India - Incidence and risk factors. British Journal of Psychiatry. 2002;181:499-504.
250. Misrawati, Afiyanti Y. Antenatal depression and its associated factors among pregnant women in Jakarta, Indonesia. Enfermeria Clinica. 2020;30:96-101.
251. Rahmadhani W, Laohasiriwong W. Gender of baby and postpartum depression among adolescent mothers in central Java, Indonesia. International Journal of Child & Adolescent Health. 2020;13(1):43-9.
252. Nurbaeti I, Deoisres W, Hengudomsub P. Association between psychosocial factors and postpartum depression in South Jakarta, Indonesia. Sexual & Reproductive Healthcare. 2019;20:72-6.
253. Nurbaeti I, Wannee D, Pornpat H. Postpartum Depression in Indonesian Mothers: Its Changes and Predicting Factors. Pacific Rim International Journal of Nursing Research. 2018;22(2):93-105.

© 2023 Mitchell AR et al. *JAMA Psychiatry.*

254. Idaiani S, Kusumawardani N, Isfandari S. The determinants of perinatal depression (PND) in Tebet Merdeka, Jakarta and Sindangbarang, Bogor Indonesia. Asean Journal of Psychiatry. 2018;19(1):54-62.
255. Edwards GD, Shinfuku N, Gittelman M, Ghozali EW, Haniman F, Wibisono S, et al. Postnatal Depression in Surabaya, Indonesia. International Journal of Mental Health. 2006;35(1):62-74.
256. Najafi-Sharjabad F, Davani N, Rayani S, Mohammadi S. Evaluation of Sociocultural, Obstetric, and Child Related Factors Associated with Postpartum Depression in Bushehr, Southwest of Iran. International Journal of Pediatrics-Mashhad. 2021;9(1):12865-76.
257. Moradi F, Azami H, Hemmatpour B, Jamasbi MM, Farahmand N, Kermani SK, et al. Factors Related to Postpartum Depression in Mothers Referred to Kermanshah Health Centers, Iran. Journal of Clinical and Diagnostic Research. 2020;14(5):VC01-VC3.
258. Khoshgoo M, Eslami O, Khadem Al-Hosseini M, Shidfar F. The Relationship between Household Food Insecurity and Depressive Symptoms among Pregnant Women: A Cross Sectional Study. Iranian Journal of Psychiatry. 2020;15(2):126-33.
259. Effati-Daryani F, Zarei S, Mohammadi A, Hemmati E, Ghasemi Yngyknd S, Mirghafourvand M. Depression, stress, anxiety and their predictors in Iranian pregnant women during the outbreak of COVID-19. BMC psychology. 2020;8(1):99.
260. Afshari P, Tadayon M, Abedi P, Yazdizadeh S. Prevalence and related factors of postpartum depression among reproductive aged women in Ahvaz, Iran. Health Care for Women International. 2020;41(3):255-65.
261. Vaezi A, Soojoodi F, Banihashemi AT, Nojomi M. The association between social support and postpartum depression in women: A cross sectional study. Women and Birth. 2019;32(2):E238-E42.
262. Parsa B, Parsa P, Boojarzadeh B, Mohammadi Y. Prediction of postpartum depression based on women's quality of life. Family Medicine and Primary Care Review. 2019;21(4):343-8.
263. Matinnia N, Ghaleiha A, Jahangard L, Ghaleiha A, Farahmand E. Psychological Risk Factors for Postnatal Depression: A Prospective Study of Iranian Low Income Primigravidae at Health Care Centres. Pertanika Journal of Social Science and Humanities. 2018;26(4):2555-69.
264. Alipour Z, Kheirabadi GR, Eslami AA, Kazemi A. Psychological profiles of risk for antenatal depression and anxiety in Iranian sociocultural context. Journal of Education & Health Promotion. 2018;7:160.
265. Abdollahi F, Zarghami M, Sazlina SG, Lye MS. Stability of depressive symptoms over 3 months post-partum. Early Intervention in Psychiatry. 2017;11(1):57-62.
266. Rouhi M, Rouhi N, Vizheh M, Salehi K. Male child preference: Is it a risk factor for antenatal depression among Iranian women? British Journal of Midwifery. 2017;25(9):572-8.
267. Mahmoodi H, Golboni F, Nadrian H, Zareipour M, Shirzadi S, Gheshlagh RG. Mother-Father Differences in Postnatal Psychological Distress and Its Determinants in Iran. Open Access Macedonian Journal of Medical Sciences. 2017;5(1):91-6.
268. Jamali S, Jahromi AR, Javadpour S, Haghbeen M. The relationship between intimate partner violence and antenatal depression: a cross-sectional study in Iran. Journal of Fundamental and Applied Sciences. 2017;9(2):1183-93.
269. Iranpour S, Kheirabadi GR, Heidari-Beni M, Maracy MR. Association between Caffeine Consumption during Pregnancy and Postpartum Depression: A Population-Based Study. Journal of Caffeine Research. 2017;7(1):1-6.
270. Moshki M, Cheravi K. Relationships among depression during pregnancy, social support and health locus of control among Iranian pregnant women. International Journal of Social Psychiatry. 2016;62(2):148-55.
271. Abdollahi F, Zarghami M, Sazlina SG, Zain AM, Mohammad AJ, Lye MS. Prediction of incidence and bio-psycho-socio-cultural risk factors of post-partum depression immediately after birth in an Iranian population. Archives of Medical Science. 2016;12(5):1043-51.
272. Abdollahi F, Etemadinezhad S, Lye MS. Postpartum mental health in relation to sociocultural practices. Taiwanese Journal of Obstetrics & Gynecology. 2016;55(1):76-80.
273. Habibzadeh A, Habibzadeh Z. Evaluation of Effective Factors and its Prevalence on Postpartum Depression Among Women in the City of Qom, Iran. International Journal of Womens Health and Reproduction Sciences. 2016;4(1):23-8.
274. Jarahi L, Zavar A, Shahi MN. Evaluation of Depression and the Contributing Factors in Pregnant Women Referring to Urban and Rural Health Care Centers of Sarakhs City, Iran. Journal of Midwifery & Reproductive Health. 2015;3(2):343-8.
275. Sadat Z, Kafaei Atrian M, Masoudi Alavi N, Abbaszadeh F, Karimian Z, Taherian A. Effect of mode of delivery on postpartum depression in Iranian women. Journal of Obstetrics & Gynaecology Research. 2014;40(1):172-7.

© 2023 Mitchell AR et al. *JAMA Psychiatry.*

276. Abdollahi F, Zarghami M, Azhar MZ, Sazlina SG, Lye MS. Predictors and incidence of post-partum depression: A longitudinal cohort study. Journal of Obstetrics and Gynaecology Research. 2014;40(12):2191-200.
277. Abdollahi F, Sazlina SG, Zain AM, Zarghami M, Asghari Jafarabadi M, Lye MS. Postpartum depression and psycho-socio-demographic predictors. Asia-Pacific psychiatry : Official Journal of the Pacific Rim College of Psychiatrists. 2014;6(4):425-34.
278. Rouhi M, Usefi H, Hasan M, Vizheh M. Ethnicity as a risk factor for postpartum depression. British Journal of Midwifery. 2012;20(6):419-26.
279. Alipour Z, Lamyian M, Hajizadeh E. Anxiety and fear of childbirth as predictors of postnatal depression in nulliparous women. Women and Birth. 2012;25(3):E37-E43.
280. Rouhi M, Mohammed-alizadeh S, Usefi H, Rouhi N. Postpartum morbidity and help-seeking behaviours in Iran. British Journal of Midwifery. 2011;19(3):178-84.
281. Abbaszadeh A, Kermani FP, Safizadeh H, Nakhee N. Violence during pregnancy and postpartum depression. Pakistan Journal of Medical Sciences. 2011;27(1):177-81.
282. Kheirabadi GR, Maracy MR. Perinatal depression in a cohort study on Iranian women. Journal of Research in Medical Sciences. 2010;15(1):41-9.
283. Tashakori A, Shanesaz A, Rezapour A. Assessment of some potential risk factors of postpartum depression. Pakistan Journal of Medical Sciences. 2009;25(2):261-4.
284. Kheirabadi GR, Maracy MR, Barekatain M, Salehi M, Sadri GH, Kelishadi M, et al. Risk factors of postpartum depression in rural areas of Isfahan Province, Iran. Archives of Iranian Medicine. 2009;12(5):461-7.
285. Montazeri A, Torkan B, Omidvari S. The Edinburgh Postnatal Depression Scale (EPDS): translation and validation study of the Iranian version. Bmc Psychiatry. 2007;7.
286. Al-Hashimi FJG, Kareem Alalaf S, Al Tawil NG. Screening for depression during pregnancy using the Kurdish version of the Edinburgh Postnatal Depression Scale in Erbil city. Health Care for Women International. 2020;41(3):240-54.
287. Ahmed HM, Alalaf SK, Al-Tawil NG. Screening for postpartum depression using Kurdish version of Edinburgh postnatal depression scale. Archives of Gynecology and Obstetrics. 2012;285(5):1249-55.
288. Bernard O, Gibson RC, McCaw-Binns A, Reece J, Coore-Desai C, Shakespeare-Pellington S, et al. Antenatal depressive symptoms in Jamaica associated with limited perceived partner and other social support: A cross-sectional study. Plos One. 2018;13(3).
289. Wissart J, Parshad O, Kulkarni S. Prevalence of pre- and postpartum depression in Jamaican women. BMC Pregnancy & Childbirth. 2005;5:15.
290. Mohammad KI, Abu Awad D, Creedy DK, Gamble J. Postpartum depression symptoms among Syrian refugee women living in Jordan. Research in Nursing & Health. 2018;41(6):519-24.
291. Safadi RR, Abushaikha LA, Ahmad MM. Demographic, maternal, and infant health correlates of post-partum depression in Jordan. Nursing & Health Sciences. 2016;18(3):306-13.
292. Abujilban SK, Abuidhail J, Al-Modallal H, Hamaideh S, Mosemli O. Predictors of Antenatal Depression Among Jordanian Pregnant Women in Their Third Trimester. Health Care for Women International. 2014;35(2):200-15.
293. Abuidhail J, Abujilban S. Characteristics of Jordanian depressed pregnant women: a comparison study. Journal of Psychiatric and Mental Health Nursing. 2014;21(7):573-9.
294. Mohammad KI, Gamble J, Creedy DK. Prevalence and factors associated with the development of antenatal and postnatal depression among Jordanian women. Midwifery. 2011;27(6):e238-45.
295. Tuthill EL, Maltby A, Conteh J, Sheira LA, Miller JD, Onono M, et al. Persistent Food Insecurity, but not HIV, is Associated with Depressive Symptoms Among Perinatal Women in Kenya: A Longitudinal Perspective. Aids and Behavior. 2021;25(3):847-55.
296. Samia P, Premji S, Tavangar F, Yim IS, Wanyonyi S, Merali M, et al. Adverse Childhood Experiences and Changing Levels of Psychosocial Distress Scores Across Pregnancy in Kenyan Women. International Journal of Environmental Research & Public Health [Electronic Resource]. 2020;17(10):13.
297. Kimbui E, Kuria M, Yator O, Kumar M. A cross-sectional study of depression with comorbid substance use dependency in pregnant adolescents from an informal settlement of Nairobi: drawing implications for treatment and prevention work. Annals of General Psychiatry. 2018;17:53.
298. Ongeri L, Wanga V, Otieno P, Mbui J, Juma E, Stoep AV, et al. Demographic, psychosocial and clinical factors associated with postpartum depression in Kenyan women. BMC Psychiatry. 2018;18(1):318.
299. Madeghe BA, Kimani VN, Vander Stoep A, Nicodimos S, Kumar M. Postpartum depression and infant feeding practices in a low income urban settlement in Nairobi-Kenya. BMC Research Notes. 2016;9(1):506.

© 2023 Mitchell AR et al. *JAMA Psychiatry.*

300. Turan B, Stringer KL, Onono M, Bukusi EA, Weiser SD, Cohen CR, et al. Linkage to HIV care, postpartum depression, and HIV-related stigma in newly diagnosed pregnant women living with HIV in Kenya: a longitudinal observational study. Bmc Pregnancy and Childbirth. 2014;14.
301. Zejnullahu VA, Ukella-Lleshi D, Zejnullahu VA, Miftari E, Govori V. Prevalence of postpartum depression at the clinic for obstetrics and gynecology in Kosovo teaching hospital: Demographic, obstetric and psychosocial risk factors. European Journal of Obstetrics & Gynecology and Reproductive Biology. 2021;256:215-20.
302. Inthaphatha S, Yamamoto E, Louangpradith V, Takahashi Y, Phengsavanh A, Kariya T, et al. Factors associated with postpartum depression among women in Vientiane Capital, Lao People's Democratic Republic: A cross-sectional study. Plos One. 2020;15(12).
303. Badr LK, Ayvazian N, Lameh S, Charafeddine L. Is the Effect of Postpartum Depression on Mother-Infant Bonding Universal? Infant Behavior & Development. 2018;51:15-23.
304. El-Hachem C, Rohayem J, Bou Khalil R, Richa S, Kesrouani A, Gemayel R, et al. Early identification of women at risk of postpartum depression using the Edinburgh Postnatal Depression Scale (EPDS) in a sample of Lebanese women. BMC Psychiatry. 2014;14:242.
305. Chaaya M, Campbell OMR, El Kak F, Shaar D, Harb H, Kaddour A. Postpartum depression: Prevalence and determinants in Lebanon. Archives of Women's Mental Health. 2002;5(2):65-72.
306. Pop-Jordanova N, Markovska-Simoska S, Filev G, Pop-Jordanov J. The need for regular screening of postpartum depression. Prilozi Makedonska Akademija Na Naukite I Umetnostite Oddelenie Za Medicinski Nauki. 2013;34(1):121-9.
307. LeMasters K, Dussault J, Barrington C, Bengtson A, Gaynes B, Go V, et al. "Pain in my heart": Understanding perinatal depression among women living with HIV in Malawi. PLoS ONE. 2020;15(6 June).
308. Chorwe-Sungani G, Chipps J. A cross-sectional study of depression among women attending antenatal clinics in Blantyre district, Malawi. The South African Journal Of Psychiatry : SAJP : The Journal Of The Society Of Psychiatrists Of South Africa. 2018;24:1181.
309. Dow A, Dube Q, Pence BW, Van Rie A. Postpartum depression and HIV infection among women in Malawi. Journal of Acquired Immune Deficiency Syndromes: JAIDS. 2014;65(3):359-65.
310. Stewart RC, Umar E, Tomenson B, Creed F. A cross-sectional study of antenatal depression and associated factors in Malawi. Archives of Women's Mental Health. 2014;17(2):145-54.
311. Elias N, & Sulaiman, S. Antenatal Depression; its Prevalence of Positive Screen and the Associating Risk Factors Including Labor and Neonatal Outcome. Current Women's Health Reviews. 2020;16:107 - 15.
312. Nasreen HE, Rahman JA, Rus RM, Kartiwi M, Sutan R, Edhborg M. Prevalence and determinants of antepartum depressive and anxiety symptoms in expectant mothers and fathers: results from a perinatal psychiatric morbidity cohort study in the east and west coasts of Malaysia. BMC Psychiatry. 2018;18(1):195.
313. Ahmed NA, Silim UA, Rosman A, Mohamed M, Chan YY, Kasim NM, et al. Postnatal depression and intimate partner violence: a nationwide clinic-based cross-sectional study in Malaysia. Bmj Open. 2018;8(5).
314. Rashid A, Mohd R. Poor social support as a risk factor for antenatal depressive symptoms among women attending public antennal clinics in Penang, Malaysia. Reproductive Health. 2017;14.
315. Mohamad Yusuff AS, Tang L, Binns CW, Lee AH. Prevalence of antenatal depressive symptoms among women in Sabah, Malaysia. Journal of Maternal-Fetal and Neonatal Medicine. 2016;29(7):1170-4.
316. Mohamad Yusuff AS, Tang L, Binns CW, Lee AH. Prevalence and risk factors for postnatal depression in Sabah, Malaysia: a cohort study. Women & Birth: Journal of the Australian College of Midwives. 2015;28(1):25-9.
317. Fadzil A, Balakrishnan K, Razali R, Sidi H, Malapan T, Japaraj RP, et al. Risk factors for depression and anxiety among pregnant women in Hospital Tuanku Bainun, Ipoh, Malaysia. Asia-Pacific Psychiatry. 2013;5(SUPPL. 1):7-13.
318. Zainal NZ, Kaka AS, Ng CG, Jawan R, Singh Gill J. Prevalence of postpartum depression in a hospital setting among Malaysian mothers. Asia-Pacific psychiatry : Official Journal of the Pacific Rim College of Psychiatrists. 2012;4(2):144-9.
319. Kadir AA, Daud MNM, Yaacob MJ, Hussain NHN. Relationship between Obstetric Risk Factors and Postnatal Depression in Malaysian Women. International Medical Journal. 2009;16(2):101-6.
320. Azidah AK, Shaiful BI, Rusli N, Jamil MY. Postnatal depression and socio-cultural practices among postnatal mothers in Kota Bahru, Kelantan, Malaysia. Medical Journal of Malaysia. 2006;61(1):76-83.
321. Kadir AA, Nordin R, Ismail SB, Yaacob MJ, Mustapha WMRW. Postnatal depression in mothers attending primary care clinics in Kelantan, Malaysia. International Medical Journal. 2005;12(2):105-9.

© 2023 Mitchell AR et al. *JAMA Psychiatry.*

322. Mahmud WMRW, Awang A, Mohamed MN. Revalidation of the Malay version of the Edinburgh postnatal depression scale (EPDS) among Malay postnatal women attending the Bakar Bata Health Center in Alor Setar, Kedah, North West of Peninsular Malaysia. Malaysian Journal of Medical Sciences. 2003;10(2):71-5.
323. Grace J, Lee KK, Ballard C, Herbert M. The relationship between post-natal depression, somatization and behaviour in Malaysian women. Transcultural Psychiatry. 2001;38(1):27-34.
324. Kit LK, Janet G, Jegasothy R. Incidence of postnatal depression in Malaysian women. Journal of Obstetrics & Gynaecology Research. 1997;23(1):85-9.
325. Raheem RA, Chih HJ, Binns CW. Maternal Depression and Breastfeeding Practices in the Maldives. Asia-Pacific Journal of Public Health. 2019;31(2):113-20.
326. Abdul Raheem R, Chih HJ, Binns CW. Factors Associated With Maternal Depression in the Maldives: A Prospective Cohort Study. Asia-Pacific Journal of Public Health. 2018;30(3):244-51.
327. Marcos-Najera R, Rodriguez-Munoz MF, Lara MA, Navarrete L, Le HN. A Cross-Cultural Analysis of the Prevalence and Risk Factors for Prenatal Depression in Spain and Mexico. Culture, Medicine & Psychiatry. 2020;24:24.
328. McRae N, Bello G, Svensson K, Solano-Gonzalez M, Wright RJ, Niedzwiecki MM, et al. Blood manganese levels during pregnancy and postpartum depression: A cohort study among women in Mexico. Neurotoxicology. 2020;76:183-90.
329. Alvarado-Esquivel C, Sifuentes-Alvarez A, Salas-Martinez C. Unhappiness with the Fetal Gender is associated with Depression in Adult Pregnant Women Attending Prenatal Care in a Public Hospital in Durango, Mexico. International Journal of Biomedical Science. 2016;12(1):36-41.
330. Lara MA, Navarrete L, Nieto L. Prenatal predictors of postpartum depression and postpartum depressive symptoms in Mexican mothers: a longitudinal study. Archives of Women's Mental Health. 2016;19(5):825-34.
331. de Castro F, Place JMS, Billings DL, Rivera L, Frongillo EA. Risk profiles associated with postnatal depressive symptoms among women in a public sector hospital in Mexico: the role of sociodemographic and psychosocial factors. Archives of Women's Mental Health. 2015;18(3):463-71.
332. Lara MA, Navarrete L, Nieto L, Martin JP, Navarro JL, Lara-Tapia H. Prevalence and incidence of perinatal depression and depressive symptoms among Mexican women. Journal of Affective Disorders. 2015;175:18-24.
333. Lara MA, Natera-Rey G, Berenzon S, Juarez-Garcia F, Villatoro-Velazquez JA, Nieto L, et al. Intimate partner violence and depressive symptoms in pregnant Mexican women: national survey results. Revista de Investigacion Clinica. 2014;66(5):431-8.
334. deCastro F, Hinojosa-Ayala N, Hernandez-Prado B. Risk and protective factors associated with postnatal depression in Mexican adolescents. Journal of Psychosomatic Obstetrics & Gynecology. 2011;32(4):210-7.
335. Pollock JI, Manaseki-Holland S, Patel V. Depression in Mongolian women over the first 2 months after childbirth: prevalence and risk factors. Journal of Affective Disorders. 2009;116(1-2):126-33.
336. Alami KM, Kadri N, Berrada S. Prevalence and psychosocial correlates of depressed mood during pregnancy and after childbirth in a Moroccan sample. Archives of Women's Mental Health. 2006;9(6):343-6.
337. Agoub M, Moussaoui D, Battas O. Prevalence of postpartum depression in a Moroccan sample. Archives of Women's Mental Health. 2005;8(1):37-43.
338. Singh DR, Sunuwar DR, Adhikari S, Singh S, Karki K. Determining factors for the prevalence of depressive symptoms among postpartum mothers in lowland region in southern Nepal. Plos One. 2021;16(1).
339. Pradhananga P, Mali P, Poudel L, Gurung M. Prevalence of Postpartum Depression in a Tertiary Health Care. Journal of Nepal Medical Association. 2020;58(223):137-40.
340. Maharjan PL, Lamichhane S, Shrestha PD, Mathias JKRSSK. Prevalence and Factors Associated with Depressive Symptoms Among Post-Partum Mothers in Dhanusha District of Nepal. Sleep & Hypnosis. 2019;21(1):60-8.
341. Khadka R, Hong SA, Chang YS. Prevalence and determinants of poor sleep quality and depression among postpartum women: a community-based study in Ramechhap district, Nepal. International Health. 2020;12(2):125-31.
342. Chalise A, Bhandari TR. Postpartum Depression and its Associated Factors: A Community-based Study in Nepal. Journal of Nepal Health Research Council. 2019;17(2):200-5.
343. Joshi D, Shrestha S, Shrestha N. Understanding the antepartum depressive symptoms and its risk factors among the pregnant women visiting public health facilities of Nepal. PLoS ONE [Electronic Resource]. 2019;14(4):e0214992.

344. Bhusal BR, Bhandari N. Identifying the factors associated with depressive symptoms among postpartum mothers in Kathmandu, Nepal. International Journal of Nursing Sciences. 2018;5(3):268-74.
345. Aryal KK, Alvik A, Thapa N, Mehata S, Roka T, Thapa P, et al. Anxiety and Depression among Pregnant Women and Mothers of Children Under one Year in Sindupalchowk District. Journal of Nepal Health Research Council. 2018;16(2):195-204.
346. Aihara Y, Shrestha S, Sharma J. Household water insecurity, depression and quality of life among postnatal women living in urban Nepal. Journal of Water and Health. 2016;14(2):317-24.
347. Kunwar D, Corey EK, Sharma P, Risal A. Screening for postpartum depression and associated factors among women who deliver at a university hospital, Nepal. Kathmandu University Medical Journal. 2015;13(49):44-8.
348. Giri RK, Khatri RB, Mishra SR, Khanal V, Sharma VD, Gartoula RP. Prevalence and factors associated with depressive symptoms among post-partum mothers in Nepal. BMC Research Notes. 2015;8:111.
349. Budhathoki N, Dahal M, Bhusal S, Ojha H, Pandey S, Basnet S. Violence against women by their husband and postpartum depression. Journal of Nepal Health Research Council. 2012;10(22):176-80.
350. Shakya R, Situala S, Shyangwa PM. Depression during Pregnancy in a Tertiary Care Center of Eastern Nepal. Journal of Nepal Medical Association. 2008;47(3):128-31.
351. Ho-Yen SD, Bondevik GT, Eberhard-Gran M, Bjorvatn B. Factors associated with depressive symptoms among postnatal women in Nepal. Acta Obstetricia et Gynecologica Scandinavica. 2007;86(3):291-7.
352. Ho-Yen SD, Bondevik GT, Eberhard-Gran M, Bjorvatn B. The prevalence of depressive symptoms in the postnatal period in Lalitpur district, Nepal. Acta Obstetricia et Gynecologica Scandinavica. 2006;85(10):1186-92.
353. Regmi S, Sligl W, Carter D, Grut W, Seear M. A controlled study of postpartum depression among Nepalese women: validation of the Edinburgh Postpartum Depression Scale in Kathmandu. Tropical Medicine & International Health. 2002;7(4):378-82.
354. Verbeek T, Arjadi R, Vendrik JJ, Burger H, Berger MY. Anxiety and depression during pregnancy in Central America: a cross-sectional study among pregnant women in the developing country Nicaragua. BMC Psychiatry. 2015;15:292.
355. Okunola TO, Awoleke JO, Olofinbiyi B, Rosiji B, Olubiyi AO, Omoya S. Predictors of postpartum depression among an obstetric population in South-Western Nigeria. Journal of Reproductive & Infant Psychology. 2021:1-13.
356. Adeyemo EO, Oluwole EO, Kanma-Okafor OJ, Izuka OM, Odeyemi KA. Prevalence and predictors of postpartum depression among postnatal women in lagos, nigeria. African Health Sciences. 2020;20(4):1943-54.
357. Oladeji BD, Bello T, Kola L, Araya R, Zelkowitz P, Gureje O. Exploring Differences Between Adolescents and Adults With Perinatal Depression-Data From the Expanding Care for Perinatal Women With Depression Trial in Nigeria. Frontiers in psychiatry Frontiers Research Foundation. 2019;10:761.
358. Oladeji BO, Bello T, Kola L, Araya R, Zelkowitz P, Gureje O. Exploring Differences Between Adolescents and Adults With Depression-Data From the Expanding Care for Perinatal Women With Depression Trial in Nigeria. Frontiers in Psychiatry. 2019;10.
359. Odinka P, Odinka J, Ezeme M, Ndukuba A, Amadi K, Muomah R, et al. Socio-demographic correlates of postpartum psychological distress among apparently healthy mothers in two tertiary hospitals in Enugu, South-East Nigeria. African Health Sciences. 2019;19(3):2515-25.
360. Agbaje OS, Anyanwu JI, Umoke PIC, Iwuagwu TE, Iweama CN, Ozoemena EL, et al. Depressive and anxiety symptoms and associated factors among postnatal women in Enugu-North Senatorial District, South-East Nigeria: a cross-sectional study. Archives of Public Health. 2019;77.
361. Tungchama F, Piwuna C, Armiya'u A, Maigari Y, Davou F, Goar S, et al. Independent socio-demographic and clinical correlates associated with the perception of quality of life of women with postpartum depressionin North-central, Nigeria. International Journal of Psychiatry in Clinical Practice. 2017;21(4):292-301.
362. Thompson O, Ajayi I. Prevalence of Antenatal Depression and Associated Risk Factors among Pregnant Women Attending Antenatal Clinics in Abeokuta North Local Government Area, Nigeria. Depression Research and Treatment. 2016;2016:4518979.
363. Sulyman D, Ayanda KA, Dattijo LM, Aminu BM. Postnatal depression and its associated factors among Northeastern Nigerian women. Annals of Tropical Medicine and Public Health. 2016;9(3):184-90.

© 2023 Mitchell AR et al. *JAMA Psychiatry.*

364. Ebeigbe PN, Akhigbe KO. Incidence and associated risk factors of postpartum depression in a tertiary hospital in Nigeria. Nigerian Postgraduate Medical Journal. 2008;15(1):15-8.
365. Adewuya AO, Ola BA, Dada AO, Fasoto OO. Validation of the Edinburgh Postnatal Depression Scale as a screening tool for depression in late pregnancy among Nigerian women. Journal of Psychosomatic Obstetrics & Gynecology. 2006;27(4):267-72.
366. Owoeye AO, Aina OF, Morakinyo O. Risk factors of postpartum depression and EPDS scores in a group of Nigerian women. Tropical Doctor. 2006;36(2):100-3.
367. Adewuya AO. Early postpartum mood as a risk factor for postnatal depression in Nigerian women. American Journal of Psychiatry. 2006;163(8):1435-7.
368. Abiodun OA. Postnatal depression in primary care populations in Nigeria. General Hospital Psychiatry. 2006;28(2):133-6.
369. Adewuya AO, Eegunranti AB, Lawal AM. Prevalence of postnatal depression in Western Nigerian women: a controlled study. International Journal of Psychiatry in Clinical Practice. 2005;9(1):60-4.
370. Adewuya AO, Fatoye FO, Ola BA, Ijaodola OR, Ibigbami SMO. Sociodemographic and obstetric risk factors for postpartum depressive symptoms in Nigerian women. Journal of Psychiatric Practice. 2005;11(5):353-8.
371. Uwakwe R, Okonkwo JE. Affective (depressive) morbidity in puerperal Nigerian women: validation of the Edinburgh Postnatal Depression Scale. Acta Psychiatrica Scandinavica. 2003;107(4):251-9.
372. Aderibigbe YA, Gureje O, Omigbodun O. Postnatal emotional disorders in Nigerian women. A study of antecedents and associations. British Journal of Psychiatry. 1993;163:645-50.
373. Aderibigbe YA, Gureje O. The validity of the 28-item General Health Questionnaire in a Nigerian antenatal clinic. Social Psychiatry & Psychiatric Epidemiology. 1992;27(6):280-3.
374. Premji SS, Lalani S, Shaikh K, Mian A, Forcheh N, Dosani A, et al. Comorbid Anxiety and Depression among Pregnant Pakistani Women: Higher Rates, Different Vulnerability Characteristics, and the Role of Perceived Stress. International Journal of Environmental Research and Public Health. 2020;17(19).
375. Yadav T, Shams R, Khan AF, Azam H, Anwar M, Anwar T, et al. Postpartum Depression: Prevalence and Associated Risk Factors Among Women in Sindh, Pakistan. Cureus. 2020;12(12).
376. Shahid A, Javed A, Rehman S, Tariq R, Ikram M, Suhail M. Evaluation of psychological impact, depression, and anxiety among pregnant women during the COVID-19 pandemic in Lahore, Pakistan. International Journal of Gynecology & Obstetrics. 2020;151(3):462-5.
377. Ishtiaque S, Sultana S, Malik U, Yaqoob U, Hussain S. Prevalence of antenatal depression and associated risk factors among pregnant women attending antenatal clinics in Karachi, Pakistan. Rawal Medical Journal. 2020;45(2):434-8.
378. Khan MJ, Hamza MA, Sarwar I, Rashid MA. Major Depressive Disorder: An Alarming Stigma Of Pregnant Women. Journal of Ayub Medical College, Abbottabad: JAMC. 2020;32(2):244-9.
379. Habiba U, Rana MS, Hanif A, Gilani SA, Asif M, Ayaz S. Prevalence and risk factors associated with prenatal depression among pregnant women in Faisalabad, Pakistan. Pakistan Journal of Pharmaceutical Sciences. 2020;33(5):2355-60.
380. Shehroz M, Kazmi A, Manzoor S. Depression and anxiety during pregnancy period. Indo American Journal of Pharmaceutical Sciences. 2019;6(5):9121-4.
381. Sikander S, Ahmad I, Atif N, Zaidi A, Vanobberghen F, Weiss HA, et al. Delivering the Thinking Healthy Programme for perinatal depression through volunteer peers: a cluster randomised controlled trial in Pakistan. Lancet Psychiatry. 2019;6(2):128-39.
382. Shagufta S, Shams S. Prevalence, Differences, and Predictors of Anxiety and Depression among Pregnant and Non-Pregnant Women in Peshawar Khyber Pakhtunkhwa Pakistan. Fwu Journal of Social Sciences. 2019;13(1):167-76.
383. Sabir M, Nagi MLF, Kazmi TH. Prevalence of antenatal depression among women receiving antenatal care during last trimester of pregnancy in a tertiary care private institute of Lahore. Pakistan Journal of Medical Sciences. 2019;35(2):527-31.
384. Naseer MH, Hussain J, Yaqub HMH. Occurrence of psychiatric Disorders Among Pregnant Females. Indo American Journal of Pharmaceutical Sciences. 2019;6(6):11522-5.
385. Ayaz I, Hanif MU, Yousaf G. A descriptive analysis of factors of depression in pregnant women of Pakistan. Indo American Journal of Pharmaceutical Sciences. 2019;6(4):7513-6.
386. Anjum F, Batool Z. An analytical study of contributory factors of postpartum depression among women in Punjab, Pakistan. Rawal Medical Journal. 2019;44(1):130-3.
387. Maselko J, Hagaman AK, Bates LM, Bhalotra S, Biroli P, Gallis JA, et al. Father involvement in the first year of life: Associations with maternal mental health and child development outcomes in rural Pakistan. Social Science and Medicine. 2019;237 (no pagination).

© 2023 Mitchell AR et al. *JAMA Psychiatry.*

388. Gul E, Muneeb PM, Azeemi MU, Khan MA, Shah S. Antenatal anxiety and depression among pregnant women attending tertiary care hospital, Mardan, Pakistan. Khyber Medical University Journal-Kmuj. 2019;11(3):160-4.
389. Zia B, Marith AM, Shahid S. Psychosocial factors causing depression during antenatal period and their association with progression of pregnancy. Indo American Journal of Pharmaceutical Sciences. 2018;5(5):4258-61.
390. Sadiq G, Shahzad Z, Sadiq S. Prospective study on prevalence and risk factors of post natal depression in Rawalpindi/Islamabad Pakistan. Rawal Medical Journal. 2018.
391. Ayyub H, Sarfraz M, Mir K, Salam FT. Association Of Antenatal Depression And Household Food Insecurity Among Pregnant Women: A Crosssectional Study From Slums Of Lahore. Journal of Ayub Medical College, Abbottabad: JAMC. 2018;30(3):366-71.
392. Jamal BA, Dastgir G, Khan MS, Iqbal N, Benish R, Khan AM, et al. Antenatal Depression: Prevalence Predictors and Frequently Employed Coping Strategies. Pakistan Journal of Medical & Health Sciences. 2018;12(2):432-6.
393. Shah S, Lonergan B. Frequency of postpartum depression and its association with breastfeeding: A cross-sectional survey at immunization clinics in Islamabad, Pakistan. Journal of the Pakistan Medical Association. 2017;67(8):1151-6.
394. Saeed A, Raana T, Saeed AM, Humayun A. Effect of antenatal depression on maternal dietary intake and neonatal outcome: a prospective cohort. Nutrition Journal. 2016;15.
395. Waqas A, Raza N, Lodhi HW, Muhammad Z, Jamal M, Rehman A. Psychosocial factors of antenatal anxiety and depression in Pakistan: is social support a mediator? PLoS ONE [Electronic Resource]. 2015;10(1):e0116510.
396. Afridi F, Batool I, Jabbar S, Hassan L, Shinwari K. Frequency of postnatal depression at a Tertiary care hospital. Journal of Medical Sciences (Peshawar). 2014;22(1):35-8.
397. Husain N, Munshi T, Jafri F, Husain M, Parveen A, Saeed Q, et al. Antenatal depression is not associated with low birth weight: A study from urban Pakistan. Frontiers in Psychiatry. 2014;5(NOV).
398. Safi FN, Khanum F, Tariq H, Mehrunisa. Antenatal depression: Prevalence and risk factors for depression among pregnant women in Peshawar. Journal of Medical Sciences (Peshawar). 2013;21(4):206-11.
399. Humayun A, Haider II, Imran N, Iqbal H, Humayun N. Antenatal depression and its predictors in Lahore, Pakistan. Eastern Mediterranean Health Journal. 2013;19(4):327-32.
400. Ali NS, Azam IS, Ali BS, Tabbusum G, Moin SS. Frequency and associated factors for anxiety and depression in pregnant women: a hospital-based cross-sectional study. Thescientificworldjournal. 2012;2012:653098.
401. Zahidie A, Kazi A, Fatmi Z, Bhatti MT, Dureshahwar S. Social environment and depression among pregnant women in rural areas of Sind, Pakistan. JPMA - Journal of the Pakistan Medical Association. 2011;61(12):1183-9.
402. Shah SMA, Bowen A, Afridi I, Nowshad G, Muhajarine N. Prevalence of Antenatal Depression: Comparison between Pakistani and Canadian women. Journal of the Pakistan Medical Association. 2011;61(3):242-6.
403. Husain N, Parveen A, Husain M, Saeed Q, Jafri F, Rahman R, et al. Prevalence and psychosocial correlates of perinatal depression: A cohort study from urban Pakistan. Archives of Women's Mental Health. 2011;14(5):395-403.
404. Imran N, Haider II. Screening of antenatal depression in Pakistan: Risk factors and effects on obstetric and neonatal outcomes. Asia-Pacific Psychiatry. 2010;2(1):26-32.
405. Muneer A, Minhas FA, Nizami ATUD, Mujeeb F, Usmani AT. Frequency and associated factors for postnatal depression. Journal of the College of Physicians and Surgeons Pakistan. 2009;19(4):236-9.
406. Karmaliani R, Asad N, Bann CM, Moss N, McClure EM, Pasha O, et al. Prevalence of anxiety, depression and associated factors among pregnant women of Hyderabad, Pakistan. International Journal of Social Psychiatry. 2009;55(5):414-24.
407. Rahman A, Creed F. Outcome of prenatal depression and risk factors associated with persistence in the first postnatal year: prospective study from Rawalpindi, Pakistan. Journal of Affective Disorders. 2007;100(1-3):115-21.
408. Kazi A, Fatmi Z, Hatcher J, Kadir MM, Niaz U, Wasserman GA. Social environment and depression among pregnant women in urban areas of Pakistan: importance of social relations. Social Science & Medicine. 2006;63(6):1466-76.
409. Husain N, Bevc I, Husain M, Chaudhry IB, Atif N, Rahman A. Prevalence and social correlates of postnatal depression in a low income country. Archives of Women's Mental Health. 2006;9(4):197-202.

© 2023 Mitchell AR et al. *JAMA Psychiatry.*

410. Niaz S, Izhar N, Bhatti MR. Anxiety and depression in pregnant women presenting in the OPD of a teaching hospital. Pakistan Journal of Medical Sciences. 2004;20(2):117-9.
411. Rahman A, Iqbal Z, Harrington R. Life events, social support and depression in childbirth: Perspectives from a rural community in the developing world. Psychological Medicine. 2003;33(7):1161-7.
412. Carroll H, Rondon MB, Sanchez SE, Fricchione GL, Williams MA, Gelaye B. Resilience mediates the relationship between household dysfunction in childhood and postpartum depression in adolescent mothers in Peru. Comprehensive Psychiatry. 2021;104.
413. Sanchez SE, Friedman LE, Rondon MB, Drake CL, Williams MA, Gelaye B. Association of stress-related sleep disturbance with psychiatric symptoms among pregnant women. Sleep Medicine. 2020;70:27-32.
414. Mitro SD, Larrabure-Torrealva GT, Sanchez SE, Molsberry SA, Williams MA, Clish C, et al. Metabolomic markers of antepartum depression and suicidal ideation. Journal of Affective Disorders. 2020;262:422-8.
415. Gelaye B, Sanchez SE, Andrade A, Gomez O, Coker AL, Dole N, et al. Association of antepartum depression, generalized anxiety, and posttraumatic stress disorder with infant birth weight and gestational age at delivery. Journal of Affective Disorders. 2020;262:310-6.
416. Friedman LE, Gelaye B, Sanchez SE, Williams MA. Association of social support and antepartum depression among pregnant women. Journal of Affective Disorders. 2020;264:201-5.
417. Gelaye B, Domingue A, Rebelo F, Friedman LE, Qiu C, Sanchez SE, et al. Association of antepartum suicidal ideation during the third trimester with infant birth weight and gestational age at delivery. Psychology Health & Medicine. 2019;24(2):127-36.
418. Larrabure-Torrealva GT, Martinez S, Luque-Fernandez MA, Sanchez SE, Mascaro PA, Ingar H, et al. Prevalence and risk factors of gestational diabetes mellitus: findings from a universal screening feasibility program in Lima, Peru. Bmc Pregnancy and Childbirth. 2018;18.
419. Friedman LE, Aponte C, Perez Hernandez R, Velez JC, Gelaye B, Sanchez SE, et al. Migraine and the risk of post-traumatic stress disorder among a cohort of pregnant women. Journal of Headache & Pain. 2017;18(1):67.
420. Gelaye B, Zhong QY, Basu A, Levey EJ, Rondon MB, Sanchez S, et al. Trauma and traumatic stress in a sample of pregnant women. Psychiatry Research. 2017;257:506-13.
421. Gelaye B, Addae G, Neway B, Larrabure-Torrealva GT, Qiu C, Stoner L, et al. Poor sleep quality, antepartum depression and suicidal ideation among pregnant women. Journal of Affective Disorders. 2017;209:195-200.
422. Friedman LE, Gelaye B, Rondon MB, Sanchez SE, Peterlin BL, Williams MA. Association of Migraine Headaches With Suicidal Ideation Among Pregnant Women in Lima, Peru. Headache. 2016;56(4):741-9.
423. Zhong QY, Wells A, Rondon MB, Williams MA, Barrios YV, Sanchez SE, et al. Childhood abuse and suicidal ideation in a cohort of pregnant Peruvian women. American journal of obstetrics and gynecology. 2016;215(4).
424. Yang N, Gelaye B, Zhong QY, Rondon MB, Sanchez SE, Williams MA. Serum brain-derived neurotrophic factor (BDNF) concentrations in pregnant women with post-traumatic stress disorder and comorbid depression. Archives of Womens Mental Health. 2016;19(6):979-86.
425. Fung J, Gelaye B, Zhong QY, Rondon MB, Sanchez SE, Barrios YV, et al. Association of decreased serum brain-derived neurotrophic factor (BDNF) concentrations in early pregnancy with antepartum depression. Bmc Psychiatry. 2015;15.
426. Cripe SM, Sanchez S, Lam N, Sanchez E, Ojeda N, Tacuri S, et al. Depressive symptoms and migraine comorbidity among pregnant Peruvian women. Journal of Affective Disorders. 2010;122(1/2):149-53.
427. Labrague LJ, McEnroe-Petitte D, Tsaras K, Yboa BC, Rosales RA, Tizon MM, et al. Predictors of postpartum depression and the utilization of postpartum depression services in rural areas in the Philippines. Perspectives in Psychiatric Care. 2020;56(2):308-15.
428. Yakupova VAS, A. Postpartum Depression and Birth Experience in Russia. Psychology in Russia: State of the Art. 2021;14(1).
429. Umuziga MP, Adejumo O, Hynie M. A cross-sectional study of the prevalence and factors associated with symptoms of perinatal depression and anxiety in Rwanda. BMC Pregnancy & Childbirth. 2020;20(1):68.
430. Niyonsenga J, Mutabaruka J. Factors of postpartum depression among teen mothers in Rwanda: a cross- sectional study. Journal of Psychosomatic Obstetrics & Gynecology. 2020:1-5.
431. Stojanov J, Stankovic M, Zikic O, Stankovic M, Stojanov A. The risk for nonpsychotic postpartum mood and anxiety disorders during the COVID-19 pandemic. International Journal of Psychiatry in Medicine. 2020:91217420981533.

© 2023 Mitchell AR et al. *JAMA Psychiatry.*

432. Odalovic M, Tadic I, Lakic D, Nordeng H, Lupattelli A, Tasic L. Translation and factor analysis of structural models of Edinburgh Postnatal Depression Scale in Serbian pregnant and postpartum women - Web-based study. Women & Birth: Journal of the Australian College of Midwives. 2015;28(3):e31-5.
433. Dmitrovic BK, Dugalic MG, Balkoski GN, Dmitrovic A, Soldatovic I. Frequency of perinatal depression in Serbia and associated risk factors. International Journal of Social Psychiatry. 2014;60(6):528-32.
434. Mare K, Pellowski, J., Koopwitz, S., Hoffman, N., van der Westhuizen, C., Wokman, L., & Stien, D. Perinatal suicidality: prevalence and correlates in a South African birth cohort. Archives of Women's Mental Health. 2021;24:737–48.
435. Mal-Sarkar T, Keyes K, Koen N, Barnett W, Myer L, Rutherford C, et al. The relationship between childhood trauma, socioeconomic status, and maternal depression among pregnant women in a South African birth cohort study. SSM Popul Health. 2021;14:100770.
436. Mokwena KE, Mbatha NL. Social and Demographic Factors Associated with Postnatal Depression Symptoms among HIV-Positive Women in Primary Healthcare Facilities, South Africa. Healthcare. 2021;9(1).
437. Phukuta N, Omole O. Prevalence and risk factors associated with postnatal depression in a South African primary care facility. African Journal of Primary Health Care & Family Medicine. 2020:2071-928.
438. Redinger S, Pearson RM, Houle B, Norris SA, Rochat TJ. Antenatal depression and anxiety across pregnancy in urban South Africa. Journal of Affective Disorders. 2020;277:296-305.
439. Modjadji P, Mokwena K. Postnatal depression screening among postpartum women attending postnatal care at selected community health centres situated in the Nkangala district of south africa. Open Public Health Journal. 2020;13(1):696-704.
440. Mbatha NL, Mokwena KE, Madiba S. Clinical and Obstetric Risk Factors for Postnatal Depression in HIV Positive Women: A Cross Sectional Study in Health Facilities in Rural KwaZulu-Natal. International Journal of Environmental Research and Public Health. 2020;17(22).
441. Mokwena K, Masike I. The Need for Universal Screening for Postnatal Depression in South Africa: Confirmation from a Sub-District in Pretoria, South Africa. International Journal of Environmental Research and Public Health. 2020;17(19).
442. Govender D, Naidoo S, Taylor M. Antenatal and Postpartum Depression: Prevalence and Associated Risk Factors among Adolescents' in KwaZulu-Natal, South Africa. Depression Research and Treatment. 2020;2020:5364521.
443. Duma N, Madiba T. The prevalence of peripartum depression and its relationship to mode of delivery and other factors among mothers in Ixopo, Kwazulu-Natal, South Africa. South African Journal of Psychology. 2020;50(4):530-9.
444. Pellowski JA, Bengtson AM, Barnett W, DiClemente K, Koen N, Zar HJ, et al. Perinatal depression among mothers in a South African birth cohort study: Trajectories from pregnancy to 18 months postpartum. Journal of Affective Disorders. 2019;259:279-87.
445. Christodoulou J, Le Roux K, Tomlinson M, Le Roux IM, Katzen LS, Rotheram-Borus MJ. Perinatal maternal depression in rural South Africa: Child outcomes over the first two years (vol 247, pg 168, 2019). Journal of Affective Disorders. 2020;274:1223-.
446. Barnett W, Pellowski J, Kuo C, Koen N, Donald KA, Zar HJ, et al. Food-insecure pregnant women in South Africa: a cross-sectional exploration of maternal depression as a mediator of violence and trauma risk factors. Bmj Open. 2019;9(3).
447. Abrahams Z, Schneider M, Field S, Honikman S. Validation of a brief mental health screening tool for pregnant women in a low socio-economic setting. BMC psychology. 2019;7(1):77.
448. Mokhele I, Nattey C, Jinga N, Mongwenyana C, Fox MP, Onoya D. Prevalence and predictors of postpartum depression by HIV status and timing of HIV diagnosis in Gauteng, South Africa. PLoS ONE [Electronic Resource]. 2019;14(4):e0214849.
449. Peltzer K, Rodriguez VJ, Lee TK, Jones D. Prevalence of prenatal and postpartum depression and associated factors among HIV-infected women in public primary care in rural South Africa: a longitudinal study. Aids Care-Psychological and Socio-Medical Aspects of Aids/Hiv. 2018;30(11):1372-9.
450. Abrahams Z, Lund C, Field S, Honikman S. Factors associated with household food insecurity and depression in pregnant South African women from a low socio-economic setting: a cross-sectional study. Social Psychiatry and Psychiatric Epidemiology. 2018;53(4):363-72.
451. MacGinty RP, Lesosky M, Barnett W, Stein DJ, Zar HJ. Associations between maternal mental health and early child wheezing in a South African birth cohort. Pediatric Pulmonology. 2018;53(6):741-54.

© 2023 Mitchell AR et al. *JAMA Psychiatry.*

452. van Heyningen T, Honikman S, Myer L, Onah MN, Field S, Tomlinson M. Prevalence and predictors of anxiety disorders amongst low-income pregnant women in urban South Africa: a cross-sectional study. Archives of Womens Mental Health. 2017;20(6):765-75.
453. Tuthill EL, Pellowski JA, Young SL, Butler LM. Perinatal Depression Among HIV-Infected Women in KwaZulu-Natal South Africa: Prenatal Depression Predicts Lower Rates of Exclusive Breastfeeding. Aids and Behavior. 2017;21(6):1691-8.
454. Pingo J, van den Heuvel LL, Vythylingum B, Seedat S. Probable postpartum hypomania and depression in a South African cohort. Archives of Womens Mental Health. 2017;20(3):427-37.
455. Nydoo P, Naicker T, Moodley J. Depressive scores in newly diagnosed HIV-infected and HIV-uninfected pregnant women. South African Journal of Psychiatry. 2017;23.
456. Brittain K, Mellins CA, Phillips T, Zerbe A, Abrams EJ, Myer L, et al. Social Support, Stigma and Antenatal Depression Among HIV-Infected Pregnant Women in South Africa. Aids and Behavior. 2017;21(1):274-82.
457. Koen N, Brittain K, Donald KA, Barnett W, Koopowitz S, Mare K, et al. Maternal Posttraumatic Stress Disorder and Infant Developmental Outcomes in a South African Birth Cohort Study. Psychological Trauma-Theory Research Practice and Policy. 2017;9(3):292-300.
458. Tsai AC, Tomlinson M, Comulada WS, Rotheram-Borus MJ. Intimate Partner Violence and Depression Symptom Severity among South African Women during Pregnancy and Postpartum: Population-Based Prospective Cohort Study. PLoS Medicine / Public Library of Science. 2016;13(1):e1001943.
459. Peltzer K, Rodriguez VJ, Jones D. Prevalence of prenatal depression and associated factors among HIV-positive women in primary care in Mpumalanga province, South Africa. SAHARA J: Journal of Social Aspects of HIV/AIDS Research Alliance. 2016;13(1):60-7.
460. Koen N, Brittain K, Donald KA, Barnett W, Koopowitz S, Mare K, et al. Psychological trauma and posttraumatic stress disorder: risk factors and associations with birth outcomes in the Drakenstein Child Health Study. European Journal of Psychotraumatology. 2016;7:28720.
461. Heyningen TV, Myer L, Onah M, Tomlinson M, Field S, Honikman S. Antenatal depression and adversity in urban South Africa. Journal of Affective Disorders. 2016;203:121-9.
462. Tomlinson M, Rotheram-Borus MJ, Harwood J, le Roux IM, O'Connor M, Worthman C. Community health workers can improve child growth of antenatally-depressed, South African mothers: a cluster randomized controlled trial. BMC Psychiatry. 2015;15:225.
463. Stellenberg EL, Abrahams JM. Prevalence of and factors influencing postnatal depression in a rural community in South Africa. African Journal of Primary Health Care & Family Medicine. 2015;7(1).
464. Stein DJ, Koen N, Donald KA, Adnams CM, Koopowitz S, Lund C, et al. Investigating the psychosocial determinants of child health in Africa: The Drakenstein Child Health Study. Journal of Neuroscience Methods. 2015;252:27-35.
465. Choi KW, Sikkema KJ, Velloza J, Marais A, Jose C, Stein DJ, et al. Maladaptive coping mediates the influence of childhood trauma on depression and PTSD among pregnant women in South Africa. Archives of Women's Mental Health. 2015;18(5):731-8.
466. Brittain K, Myer L, Koen N, Koopowitz S, Donald KA, Barnett W, et al. Risk factors for antenatal depression and associations with infant birth outcomes: Results from a south african birth cohort study. Paediatric and Perinatal Epidemiology. 2015;29(6):504-14.
467. Baron E, Field S, Kafaar Z, Honikman S. Patterns of use of a maternal mental health service in a low-resource antenatal setting in South Africa. Health & Social Care in the Community. 2015;23(5):502-12.
468. Tomlinson M, O'Connor MJ, le Roux IM, Stewart J, Mbewu N, Harwood J, et al. Multiple risk factors during pregnancy in South Africa: the need for a horizontal approach to perinatal care. Prevention Science. 2014;15(3):277-82.
469. Rochat TJ, Tomlinson M, Newell ML, Stein A. Detection of antenatal depression in rural HIV-affected populations with short and ultrashort versions of the Edinburgh Postnatal Depression Scale (EPDS). Archives of Women's Mental Health. 2013;16(5):401-10.
470. Dewing S, Tomlinson M, le Roux IM, Chopra M, Tsai AC. Food insecurity and its association with co-occurring postnatal depression, hazardous drinking, and suicidality among women in peri-urban South Africa. Journal of Affective Disorders. 2013;150(2):460-5.
471. Vythilingum B, Roos A, Faure SC, Geerts L, Stein DJ. Risk factors for substance use in pregnant women in South Africa. Samj South African Medical Journal. 2012;102(11):851-4.
472. Manikkam L, Burns JK. Antenatal depression and its risk factors: an urban prevalence study in KwaZulu-Natal. South African Medical Journal Suid-Afrikaanse Tydskrif Vir Geneeskunde. 2012;102(12):940-4.

© 2023 Mitchell AR et al. *JAMA Psychiatry.*

473. Rochat TJ, Tomlinson M, Bärnighausen T, Newell ML, Stein A, Rochat TJ, et al. The prevalence and clinical presentation of antenatal depression in rural South Africa. Journal of Affective Disorders. 2011;135(1-3):362-73.
474. Peltzer K, Shikwane ME. Prevalence of postnatal depression and associated factors among HIV-positive women in primary care in Nkangala District, South Africa. Southern African Journal of HIV Medicine. 2011(42):24-8.
475. Hartley M, Tomlinson M, Greco E, Comulada WS, Stewart J, le Roux I, et al. Depressed mood in pregnancy: Prevalence and correlates in two Cape Town peri-urban settlements. Reproductive Health. 2011;8.
476. Ramchandani PG, Richter LM, Stein A, Norris SA. Predictors of postnatal depression in an urban South African cohort. Journal of Affective Disorders. 2009;113(3):279-84.
477. Cooper PJ, Tomlinson M, Swartz L, Woolgar M, Murray L, Molteno C. Post-partum depression and the mother-infant relationship in a South African peri-urban settlement. British Journal of Psychiatry. 1999;175:554-8.
478. Patabendige M, Gamage MM, Weerasinghe M, Jayawardane A. Psychological impact of the COVID-19 pandemic among pregnant women in Sri Lanka. International Journal of Gynecology & Obstetrics. 2020;151(1):150-3.
479. Fan QP, Long Q, De Silva V, Gunarathna N, Jayathilaka U, Dabrera T, et al. Prevalence and risk factors for postpartum depression in Sri Lanka: A population-based study. Asian Journal of Psychiatry. 2020;47.
480. Arachchi NSM, Ganegama R, Husna AWF, Chandima DL, Hettigama N, Premadasa J, et al. Suicidal ideation and intentional self-harm in pregnancy as a neglected agenda in maternal health; an experience from rural Sri Lanka. Reproductive Health. 2019;16(1):166.
481. Herath INS, Balasuriya A, Siyayogan S. Physical and psychological morbidities among selected antenatal females in Kegalle district of Sri Lanka: A cross sectional study. Journal of Obstetrics and Gynaecology. 2017;37(7):849-54.
482. Agampodi SB, Agampodi TC. Antenatal depression in Anuradhapura, Sri Lanka and the factor structure of the Sinhalese version of Edinburgh post partum depression scale among pregnant women. PLoS ONE [Electronic Resource]. 2013;8(7):e69708.
483. Khalifa DS, Glavin K, Bjertness E, Lien L. Course of depression symptoms between 3 and 8 months after delivery using two screening tools (EPDS and HSCL-10) on a sample of Sudanese women in Khartoum state. Bmc Pregnancy and Childbirth. 2018;18.
484. Khalifa DS, Glavin K, Bjertness E, Lien L. Determinants of postnatal depression in Sudanese women at 3 months postpartum: a cross-sectional study. BMJ Open. 2016;6(3):e009443.
485. Khalifa DS, Glavin K, Bjertness E, Lien L. Postnatal depression among Sudanese women: prevalence and validation of the Edinburgh Postnatal Depression Scale at 3 months postpartum. International Journal of Women's Health. 2015;7:677-84.
486. Roumieh M, Bashour H, Kharouf M, Chaikha S. Prevalence and risk factors for postpartum depression among women seen at Primary Health Care Centres in Damascus. BMC Pregnancy & Childbirth. 2019;19(1):1-5.
487. Manongi R, Rogathi J, Sigalla G, Mushi D, Rasch V, Gammeltoft T, et al. The Association Between Intimate Partner Violence and Signs of Depression During Pregnancy in Kilimanjaro Region, Northern Tanzania. Journal of Interpersonal Violence. 2020;35(23-24):5797-811.
488. Herlosky KN, Benyshek DC, Mabulla IA, Pollom TR, Crittenden AN. Postpartum Maternal Mood Among Hadza Foragers of Tanzania: A Mixed Methods Approach. Culture Medicine and Psychiatry. 2020;44(3):305-32.
489. Ngocho JS, Watt MH, Minja L, Knettel BA, Mmbaga BT, Williams PP, et al. Depression and anxiety among pregnant women living with HIV in Kilimanjaro region, Tanzania. PLoS ONE [Electronic Resource]. 2019;14(10):e0224515.
490. Holm-Larsen CE, Madsen FK, Rogathi JJ, Manongi R, Mushi D, Meyrowitsch DW, et al. Postpartum depression and child growth in Tanzania: a cohort study. BJOG: An International Journal of Obstetrics & Gynaecology. 2019;126(5):590-8.
491. Mahenge B, Stockl H, Mizinduko M, Mazalale J, Jahn A. Adverse childhood experiences and intimate partner violence during pregnancy and their association to postpartum depression. Journal of Affective Disorders. 2018;229:159-63.
492. Rogathi JJ, Manongi R, Mushi D, Rasch V, Sigalla GN, Garnmeltoft T, et al. Postpartum depression among women who have experienced intimate partner violence: A prospective cohort study at Moshi, Tanzania. Journal of Affective Disorders. 2017;218:238-45.

© 2023 Mitchell AR et al. *JAMA Psychiatry.*

493. Kaaya S, Garcia ME, Li N, Lienert J, Twayigize W, Spiegelman D, et al. Association of maternal depression and infant nutritional status among women living with HIV in Tanzania. Maternal & Child Nutrition. 2016;12(3):603-13.
494. Rwakarema M, Premji SS, Nyanza EC, Riziki P, Palacios-Derflingher L. Antenatal depression is associated with pregnancy-related anxiety, partner relations, and wealth in women in Northern Tanzania: a cross-sectional study. Bmc Womens Health. 2015;15.
495. Mahenge B, Stockl H, Likindikoki S, Kaaya S, Mbwambo J. The prevalence of mental health morbidity and its associated factors among women attending a prenatal clinic in Tanzania. International Journal of Gynaecology & Obstetrics. 2015;130(3):261-5.
496. Mahenge B, Likindikoki S, Stockl H, Mbwambo J. Intimate partner violence during pregnancy and associated mental health symptoms among pregnant women in Tanzania: a cross-sectional study. Bjog-an International Journal of Obstetrics and Gynaecology. 2013;120(8):940-7.
497. Kaaya SF, Mbwambo JK, Kilonzo GP, Van Den Borne H, Leshabari MT, Smith Fawzi MC, et al. Socio-economic and partner relationship factors associated with antenatal depressive morbidity among pregnant women in Dar es Salaam, Tanzania. Tanzania Journal of Health Research. 2010;12(1):3.
498. Tuksanawes P, Kaewkiattikun K, Kerdcharoen N. Prevalence and Associated Factors of Antenatal Depressive Symptoms in Pregnant Women Living in an Urban Area of Thailand. International Journal of Womens Health. 2020;12:849-58.
499. Fellmeth G, Plugge E, Fazel M, Oo MM, Pimanpanarak M, Phichitpadungtham Y, et al. Prevalence and determinants of perinatal depression among labour migrant and refugee women on the Thai-Myanmar border: a cohort study. BMC Psychiatry. 2020;20(1):168.
500. Phoosuwan N, Eriksson L, Lundberg PC. Antenatal depressive symptoms during late pregnancy among women in a north-eastern province of Thailand: Prevalence and associated factors. Asian Journal of Psychiatry. 2018;36:102-7.
501. Hassert S, Sharon SR, Payakkakom A, Kodysova E. Postpartum Depressive Symptoms: Risks for Czech and Thai Mothers. Journal of Perinatal Education. 2018;27(1):38-49.
502. Roomruangwong C, Withayavanitchai S, Maes M. Antenatal and postnatal risk factors of postpartum depression symptoms in Thai women: A case-control study. Sexual & reproductive healthcare : official journal of the Swedish Association of Midwives. 2016;10:25-31.
503. Uthaipaisanwong A, Rungruxsirivorn T, Roomruangwong C, Taechakraichana N, Chaithongwongwatthana S. Associated factors of prenatal depression among teenage pregnant women at King Chulalongkorn Memorial Hospital. Journal of the Medical Association of Thailand. 2015;98(5):437-43.
504. Panyayong B. Postpartum depression among Thai women: a national survey. Journal of the Medical Association of Thailand. 2013;96(7):761-7.
505. Ross R, Sawatphanit W, Mizuno M, Takeo K. Depressive symptoms among HIV-positive postpartum women in Thailand. Archives of Psychiatric Nursing. 2011;25(1):36-42.
506. Ross R, Sawatphanit W, Zeller R. Depressive symptoms among HIV-positive pregnant women in Thailand. Journal of Nursing Scholarship. 2009;41(4):344-50.
507. Liabsuetrakul T, Vittayanont A, Pitanupong J. Clinical applications of anxiety, social support, stressors, and self-esteem measured during pregnancy and postpartum for screening postpartum depression in Thai women. Journal of Obstetrics and Gynaecology Research. 2007;33(3):333-40.
508. Limlomwongse N, Liabsuetrakul T. Cohort study of depressive moods in Thai women during late pregnancy and 6-8 weeks of postpartum using the Edinburgh Postnatal Depression Scale (EPDS). Archives of Women's Mental Health. 2006;9(3):131-8.
509. Rees SJ, Tol W, Mohsin M, Tay AK, Tam N, dos Reis N, et al. A high-risk group of pregnant women with elevated levels of conflict-related trauma, intimate partner violence, symptoms of depression and other forms of mental distress in post-conflict Timor-Leste (vol 6, e725, 2016). Translational Psychiatry. 2016;6.
510. Silove D, Rees S, Tay AK, da Costa ZM, Savio ES, Soares C, et al. Pathways to perinatal depressive symptoms after mass conflict in Timor-Leste: a modelling analysis using cross-sectional data. The Lancet Psychiatry. 2015;2(2):161-7.
511. Guvenc G, Yesilcinar I, Ozkececi F, Oksuz E, Ozkececi CF, Konukbay D, et al. Anxiety, depression, and knowledge level in postpartum women during the COVID-19 pandemic. Perspectives in Psychiatric Care. 2020;18:18.
512. Boran P, Waqas A, Askan OO, Topcu I, Dogan T, Rahman A. Screening of postpartum depression among new mothers in Istanbul: a psychometric evaluation of the Turkish Edinburgh Postnatal Depression Scale. Bmc Research Notes. 2020;13(1).
513. Sut HK, Kucukkaya B. Anxiety, depression, and related factors in pregnant women during the COVID-19 pandemic in Turkey: A web-based cross-sectional study. Perspectives in Psychiatric Care. 2020.

© 2023 Mitchell AR et al. *JAMA Psychiatry.*

514. Oskovi-Kaplan ZA, Buyuk GN, Ozgu-Erdinc AS, Keskin HL, Ozbas A, Moraloglu Tekin O. The Effect of COVID-19 Pandemic and Social Restrictions on Depression Rates and Maternal Attachment in Immediate Postpartum Women: a Preliminary Study. Psychiatric Quarterly. 2020;04:04.
515. Kizilirmak A, Calpbinici P, Tabakan G, Kartal B. Correlation between postpartum depression and spousal support and factors affecting postpartum depression. Health Care for Women International.
516. Cankaya S. The effect of psychosocial risk factors on postpartum depression in antenatal period: A prospective study. Archives of Psychiatric Nursing. 2020;34(3):176-83.
517. Aydemir S, Onan N. The Relationship Between Maternal Self-confidence and Postpartum Depression in Primipara Mothers: A Follow-Up Study. Community Mental Health Journal. 2020;56(8):1449-56.
518. Anık Y, Ege E. The Relationship between psychosocial health status and risk of depression among pregnant women in Turkey. Journal of Midwifery & Reproductive Health. 2020;8(2):2190-9.
519. Topatan S, Demirci N. Frequency of Depression and Risk Factors among Adolescent Mothers in Turkey within the First Year of the Postnatal Period. Journal of Pediatric and Adolescent Gynecology. 2019;32(5):514-9.
520. Sahin E, Seven M. Depressive symptoms during pregnancy and postpartum: a prospective cohort study. Perspectives in Psychiatric Care. 2019;55(3):430-7.
521. Oztora S, Arslan A, Caylan A, Dagdeviren HN. Postpartum depression and affecting factors in primary care. Nigerian Journal of Clinical Practice. 2019;22(1):85-91.
522. Nacar G, Tashan ST. Relationship between sleep characteristics and depressive symptoms in last trimester of pregnancy. Afr Health Sci. 2019;19(4):2934-44.
523. Unsal Atan Ş, Ozturk R, Gulec Satir D, Ildan Çalim S, Karaoz Weller B, Amanak K, et al. Relation between mothers' types of labor, birth interventions, birth experiences and postpartum depression: A multicentre follow-up study. Sexual & Reproductive HealthCare. 2018;18:13-8.
524. Duman B, Cankorur VS, Taylor C, Stewart R. Prospective associations between recalled parental bonding and perinatal depression: a cohort study in urban and rural Turkey (vol 53, pg 385, 2018). Social Psychiatry and Psychiatric Epidemiology. 2018;53(7):763-.
525. Capik A, Durmaz H. Fear of Childbirth, Postpartum Depression, and Birth-Related Variables as Predictors of Posttraumatic Stress Disorder After Childbirth. Worldviews on Evidence-Based Nursing. 2018;15(6):455-63.
526. Yildiz G, Senturk MB, Yildiz P, Cakmak Y, Budak MS, Cakar E. Serum serotonin, leptin, and adiponectin changes in women with postpartum depression: controlled study. Arch Gynecol Obstet. 2017;295(4):853-8.
527. Dikmen-Yildiz P, Ayers S, Phillips L. Depression, anxiety, PTSD and comorbidity in perinatal women in Turkey: A longitudinal population-based study. Midwifery. 2017;55:29-37.
528. Cankorur VS, Duman B, Taylor C, Stewart R. Gender preference and perinatal depression in Turkey: A cohort study. Plos One. 2017;12(3).
529. Celik SB, Bucaktepe GE, Uludag A, Bulut IU, Erdem O, Altinbas K. Screening mixed depression and bipolarity in the postpartum period at a primary health care center. Comprehensive Psychiatry. 2016;71:57-62.
530. Bolak Boratav H, Toker O, Kuey L. Postpartum depression and its psychosocial correlates: A longitudinal study among a group of women in Turkey. Women & Health. 2016;56(5):502-21.
531. Turkcapar AF, Kadioglu N, Aslan E, Tunc S, Zayifoglu M, Mollamahmutoglu L. Sociodemographic and clinical features of postpartum depression among Turkish women: a prospective study. Bmc Pregnancy and Childbirth. 2015;15.
532. Aktas S, Yesilcicek Calik K. Factors Affecting Depression During Pregnancy and the Correlation Between Social Support and Pregnancy Depression. Iranian Red Crescent Medical Journal. 2015;17(9):e16640.
533. Kirkan TS, Aydin N, Yazici E, Aslan PA, Acemoglu H, Daloglu AG. The depression in women in pregnancy and postpartum period: A follow-up study. International Journal of Social Psychiatry. 2015;61(4):343-9.
534. Akcali Aslan P, Aydin N, Yazici E, Aksoy AN, Kirkan TS, Daloglu GA. Prevalence of depressive disorders and related factors in women in the first trimester of their pregnancies in Erzurum, Turkey. International Journal of Social Psychiatry. 2014;60(8):809-17.
535. Pocan AG, Aki OE, Parlakgumus AH, Gereklioglu C, Dolgun AB. The incidence of and risk factors for postpartum depression at an urban maternity clinic in Turkey. International Journal of Psychiatry in Medicine. 2013;46(2):179-94.
536. Orun E, Yalcin SS, Mutlu B. Relations of maternal psychopathologies, social-obstetrical factors and mother-infant bonding at 2-month postpartum: a sample of Turkish mothers. World Journal of Pediatrics. 2013;9(4):350-5.

© 2023 Mitchell AR et al. *JAMA Psychiatry.*

537. Yanikkerem E, Ay S, Mutlu S, Goker A. Antenatal depression: prevalence and risk factors in a hospital based Turkish sample. JPMA - Journal of the Pakistan Medical Association. 2013;63(4):472-7.
538. Serhan N, Ege E, Ayranci U, Kosgeroglu N. Prevalence of postpartum depression in mothers and fathers and its correlates. Journal of Clinical Nursing. 2013;22(1-2):279-84.
539. Senturk V, Abas M, Berksun O, Stewart R. Social support and antenatal depression in extended and nuclear family environments in Turkey: a cross-sectional survey. BMC Psychiatry. 2011;11:48.
540. Ozbasaran F, Coban A, Kucuk M. Prevalence and risk factors concerning postpartum depression among women within early postnatal periods in Turkey. Archives of Gynecology & Obstetrics. 2011;283(3):483-90.
541. Bodur S, Ozdemir S, Ayvaz K. Risk factors for postpartum depression in a group of teenage mothers. Turkiye Klinikleri Jinekoloji Obstetrik. 2010;20(1):22-8.
542. Yagmur Y, Ulukoca N, Yağmur Y, Ulukoca N. Social support and postpartum depression in low-socioeconomic level postpartum women in Eastern Turkey. International Journal of Public Health. 2010;55(6):543-9.
543. Kirpinar I, Gozum S, Pasinlioglu T. Prospective study of postpartum depression in eastern Turkey prevalence, socio-demographic and obstetric correlates, prenatal anxiety and early awareness. Journal of Clinical Nursing. 2010;19(3-4):422-31.
544. Golbasi Z, Kelleci M, Kisacik G, Cetin A. Prevalence and correlates of depression in pregnancy among Turkish women. Maternal & Child Health Journal. 2010;14(4):485-91.
545. Karacam Z, Ancel G. Depression, anxiety and influencing factors in pregnancy: a study in a Turkish population. Midwifery. 2009;25(4):344-56.
546. Ege E, Timur S, Zincir H, Geckil E, Sunar-Reeder B. Social support and symptoms of postpartum depression among new mothers in Eastern Turkey. Journal of Obstetrics & Gynaecology Research. 2008;34(4):585-93.
547. Kara B, Unalan P, Cifcili S, Cebeci DS, Sarper N. Is there a role for the family and close community to help reduce the risk of postpartum depression in new mothers? A cross-sectional study of Turkish women. Maternal & Child Health Journal. 2008;12(2):155-61.
548. Caliskan D, Oncu B, Kose K, Ocaktan ME, Ozdemir O. Depression scores and associated factors in pregnant and non-pregnant women: A community-based study in Turkey. Journal of Psychosomatic Obstetrics & Gynecology. 2007;28(4):195-200.
549. Akman C, Uguz F, Kaya N. Postpartum-onset major depression is associated with personality disorders. Comprehensive Psychiatry. 2007;48(4):343-7.
550. Dindar I, Erdogan S. Screening of Turkish women for postpartum depression within the first postpartum year: The risk profile of a community sample. Public Health Nursing. 2007;24(2):176-83.
551. Gulseren L, Erol A, Gulseren S, Kuey L, Kilic B, Ergor G. From antepartum to postpartum: a prospective study on the prevalence of peripartum depression in a semiurban Turkish community. Journal of Reproductive Medicine. 2006;51(12):955-60.
552. Ozdemir H, Ergin N, Selimoglu K, Bilgel N. Postnatal depressive mood in Turkish women. Psychology, Health and Medicine. 2005;10(1):96-107.
553. Aydin N, Inandi T, Karabulut N. Depression and associated factors among women within their first postnatal year in Erzurum Province in Eastern Turkey. Women & Health. 2005;41(2):1-12.
554. Inandi T, Bugdayci R, Dundar P, Sumer H, Sasmaz T. Risk factors for depression in the first postnatal year: a Turkish study. Social Psychiatry & Psychiatric Epidemiology. 2005;40(9):725-30.
555. Bugdayci R, Sasmaz CT, Tezcan H, Kurt AO, Oner S. A cross-sectional prevalence study of depression at various times after delivery in Mersin province in Turkey. Journal of Women's Health. 2004;13(1):63-8.
556. Aydin N, Inandi T, Yigit A, Nalan Sahin Hodoglugil N. Validation of the Turkish version of the Edinburgh Postnatal Depression Scale among women within their first postpartum year. Social Psychiatry and Psychiatric Epidemiology. 2004;39(6):483-6.
557. Ekuklu G, Tokuc B, Eskiocak M, Berberoglu U, Saltik A. Prevalence of postpartum depression in Edirne, Turkey, and related factors. Journal of Reproductive Medicine. 2004;49(11):908-14.
558. Inandi T, Elci OC, Ozturk A, Egri M, Polat A, Sahin TK. Risk factors for depression in postnatal first year, in eastern Turkey. International Journal of Epidemiology. 2002;31(6):1201-7.
559. Danaci AE, Dinc G, Deveci A, Sen FS, Icelli I. Postnatal depression in turkey: epidemiological and cultural aspects. Social Psychiatry & Psychiatric Epidemiology. 2002;37(3):125-9.
560. Arach AAO, Nakasujja N, Nankabirwa V, Ndeezi G, Kiguli J, Mukunya D, et al. Perinatal death triples the prevalence of postpartum depression among women in Northern Uganda: A community-based cross-sectional study. Plos One. 2020;15(10).
561. Nampijja M, Natamba B, Mpango R, Kinyanda E. The burden and risk factors for postnatal depression and depressive symptomatology among women in Kampala. Tropical Doctor. 2019;49(3):170-7.

© 2023 Mitchell AR et al. *JAMA Psychiatry.*

562. Natamba BK, Achan J, Arbach A, Oyok TO, Ghosh S, Mehta S, et al. Reliability and validity of the center for epidemiologic studies-depression scale in screening for depression among HIV-infected and -uninfected pregnant women attending antenatal services in northern Uganda: a cross-sectional study. BMC Psychiatry. 2014;14:303.
563. Kakyo TA, Muliira JK, Mbalinda SN, Kizza IB, Muliira RS. Factors associated with depressive symptoms among postpartum mothers in a rural district in Uganda. Midwifery. 2012;28(3):374-9.
564. Bailey H, Malyuta R, Semenenko I, Townsend CL, Cortina-Borja M, Thorne C, et al. Prevalence of depressive symptoms in pregnant and postnatal HIV-positive women in Ukraine: a cross-sectional survey. Reproductive Health. 2016;13:27.
565. Luong-Thanh BY, Nguyen LH, Murray L, Eisner M, Valdebenito S, Hoang TD, et al. Depression and its associated factors among pregnant women in central Vietnam. Health Psychology Open. 2021;8(1):2055102920988445.
566. Hue MT, Van NHN, Nha PP, Vu NT, Duc PM, Trang NTV, et al. Factors associated with antenatal depression among pregnant women in Vietnam: A multisite cross-sectional survey. Health Psychology Open. 2020;7(1).
567. Tho Nhi T, Hanh NTT, Hinh ND, Toan NV, Gammeltoft T, Rasch V, et al. Intimate Partner Violence among Pregnant Women and Postpartum Depression in Vietnam: A Longitudinal Study. BioMed Research International. 2019;2019:4717485.
568. Van Ngo T, Gammeltoft T, Nguyen HTT, Meyrowitsch DW, Rasch V. Antenatal depressive symptoms and adverse birth outcomes in Hanoi, Vietnam. PLoS ONE [Electronic Resource]. 2018;13(11):e0206650.
569. Tran NT, Nguyen HTT, Nguyen HD, Ngo TV, Gammeltoft T, Rasch V, et al. Emotional violence exerted by intimate partners and postnatal depressive symptoms among women in Vietnam: A prospective cohort study. Plos One. 2018;13(11).
570. Vo TV, Hoa TKD, Hoang TD. Postpartum Depressive Symptoms and Associated Factors in Married Women: A Cross-sectional Study in Danang City, Vietnam. Frontiers in Public Health. 2017;5.
571. Murray L, Dunne MP, Vo TV, Anh PNT, Khawaja NG, Cao TN. Postnatal depressive symptoms amongst women in Central Vietnam: a cross-sectional study investigating prevalence and associations with social, cultural and infant factors. Bmc Pregnancy and Childbirth. 2015;15.
572. Suzuki Y, Goto A, Vinh NQ, Van NTT, Minh PN, Thuy CTM, et al. Postnatal depression and associated parenting indicators among Vietnamese women. Asia-Pacific Psychiatry. 2011;3(4):219-27.
573. Fisher J, Tran T, La BT, Kriitmaa K, Rosenthal D, Tran T. Common perinatal mental disorders in northern Viet Nam: community prevalence and health care use. Bulletin of the World Health Organization. 2010;88(10):737-45.
574. Fisher JRW, Tran HTT, Tran T. Relative socioeconomic advantage and mood during advanced pregnancy in women in Vietnam. International Journal of Mental Health Systems. 2007;1 (no pagination).
575. Fisher JRW, Morrow MM, Ngoc NTN, Anh LTH, Fisher JRW, Morrow MM, et al. Prevalence, nature, severity and correlates of postpartum depressive symptoms in Vietnam. BJOG: An International Journal of Obstetrics & Gynaecology. 2004;111(12):1353-60.
576. Qandil S, Jabr S, Wagler S, Collin SM. Postpartum depression in the Occupied Palestinian Territory: a longitudinal study in Bethlehem. Bmc Pregnancy and Childbirth. 2016;16.
577. Ndokera R, MacArthur C. The relationship between maternal depression and adverse infant health outcomes in Zambia: a cross-sectional feasibility study. Child: Care, Health & Development. 2011;37(1):74-81.
578. Kakirau-Hagali M. Study of depression and anxiety in prenatal and postnatal women at Port Moresby General Hospital. Papua New Guinea Medical Journal. 2010;53(1-2):30-6.
579. Nyamukoho E, Mangezi W, Marimbe B, Verhey R, Chibanda D. Depression among HIV positive pregnant women in Zimbabwe: a primary health care based cross-sectional study. Bmc Pregnancy and Childbirth. 2019;19.
580. January J, Chimbari MJ. Prevalence and factors associated with postnatal depression among women in two rural districts of Manicaland, Zimbabwe. South African Journal of Psychiatry. 2018;24.
581. January J, Mutamba N, Maradzika J. Correlates of postnatal depression among women in Zimbabwean semi-urban and rural settings. Journal of Psychology in Africa. 2017;27(1):93-6.
582. Shamu S, Zarowsky C, Roelens K, Temmerman M, Abrahams N. High-frequency intimate partner violence during pregnancy, postnatal depression and suicidal tendencies in Harare, Zimbabwe. General Hospital Psychiatry. 2016;38(1):109-14.
583. January J, Chivanhu H, Chiwara J, Denga T, Dera K, Dube T, et al. Prevalence and the correlates of postnatal depression in an urban high density suburb of Harare. Central African Journal of Medicine. 2015;61(1-4):1-4.

© 2023 Mitchell AR et al. *JAMA Psychiatry.*

584. Chibanda D, Mangezi W, Tshimanga M, Woelk G, Rusakaniko S, Stranix-Chibanda L, et al. Postnatal depression by HIV status among women in Zimbabwe. Journal of Women's Health. 2010;19(11):2071-7.
585. Wesselhoeft R, Madsen FK, Lichtenstein MB, Sibbersen C, Manongi R, Mushi DL, et al. Postnatal depressive symptoms display marked similarities across continents. Journal of Affective Disorders. 2020;261:58-66.
586. McCauley M, Madaj B, White SA, Dickinson F, Bar-Zev S, Aminu M, et al. Burden of physical, psychological and social ill-health during and after pregnancy among women in India, Pakistan, Kenya and Malawi. Bmj Global Health. 2018;3(3).
587. Bindt C, Guo N, Te Bonle M, Appiah-Poku J, Hinz R, Barthel D, et al. No association between antenatal common mental disorders in low-obstetric risk women and adverse birth outcomes in their offspring: Results from the CDS study in Ghana and Cote D'Ivoire. PLoS ONE. 2013;8(11).
588. Guo N, Bindt C, Te Bonle M, Appiah-Poku J, Hinz R, Barthel D, et al. Association of Antepartum and Postpartum Depression in Ghanaian and Ivorian Women With Febrile Illness in Their Offspring: A Prospective Birth Cohort Study. American Journal of Epidemiology. 2013;178(9):1394-402.
589. Bindt C, Appiah-Poku J, Te Bonle M, Schoppen S, Feldt T, Barkmann C, et al. Antepartum depression and anxiety associated with disability in African women: cross-sectional results from the CDS study in Ghana and Cote d'Ivoire. PLoS ONE [Electronic Resource]. 2012;7(10):e48396.

© 2023 Mitchell AR et al. *JAMA Psychiatry.*
