# Supplementary material for: Prevalence of Perinatal Depression in Low- and Middle-Income Countries: A Systematic Review and Meta-analysis
Source: JAMA Psychiatry. 2023 Mar 8;80(5):425–31. doi: 10.1001/jamapsychiatry.2023.0069 (PMC9996459; doi:10.1001/jamapsychiatry.2023.0069)
Supplement: Supplement 2. — Data sharing statement [file jamapsychiatry-e230069-s002.pdf]

Data Sharing Statement

Roddy Mitchell. Prevalence of Perinatal Depression in Low- and Middle-Income Countries. *JAMA Psychiatry*. Published March 08, 2023. doi:10.1001/jamapsychiatry.2023.0069

Data
Data available: No

Additional Information
Explanation for why data not available: Data will be made available upon reasonable request
